# Synthesis, biological evaluation and molecular docking of new triphenylamine-linked pyridine, thiazole and pyrazole analogues as anticancer agents

**DOI:** 10.1186/s13065-022-00879-x

**Published:** 2022-11-07

**Authors:** Mohamed R. Elmorsy, Samar E. Mahmoud, Ahmed A. Fadda, Ehab Abdel-Latif, Miral A. Abdelmoaz

**Affiliations:** 1 Department of Chemistry, Faculty of Science, Mansoura University, Mansoura, 35516 Egypt; 2 Department of Pharmaceutical Chemistry, Faculty of Pharmacy, Sinai University, Kantra, Egypt

**Keywords:** 2-Pyridone, Thiazolidin-5-one, Molecular docking, Lung cancer, Breast cancer

## Abstract

A new series of pyridine, thiazole, and pyrazole analogues were synthesized. The pyridone analogues **4a-e** were synthesized by treating *N*-aryl-2-cyano-3-(4-(diphenylamino)phenyl)acrylamides **3a-e** with malononitrile. Many 4-arylidene-thiazolidin-5-one analogues **6a-d** were obtained by Knoevenagel reactions of 4-(diphenylamino)benzaldehyde (**1**) with their corresponding thiazolidin-5-one derivatives **5a-d**. The structural elucidation of the products was proven by the collections of spectroscopic methods such as IR, ^1^H NMR, ^13^C NMR, and MS data. Their anti-cancer activity was examined against two cell lines, **MDA-MB-231** (mammary carcinomas) and **A-549** (lung cancer). Compared with cisplatin as a reference standard drug, 6-amino-4-(4-(diphenylamino)phenyl)-2-oxo-1-(*p*-tolyl)-1,2-dihydropyridine-3,5-dicarbonitrile (**4b**) and 6-amino-4-(4-(diphenylamino)phenyl)-1-(4-nitrophenyl)-2-oxo-1,2-dihydropyridine-3,5-dicarbonitrile (**4e**) exhibited better efficiency against the **A-549** cell line, with IC_50_ = 0.00803 and 0.0095 μM, respectively. Also, these compounds **4b** and **4e** showed the most potency among the examined compounds against **MDA-MB-231** with IC_50_ = 0.0103 and 0.0147 μM, respectively. The newly synthesized compounds were docked inside the active sites of the selected proteins and were found to demonstrate proper binding. 2-Cyano-2-(4,4-(diphenylamino)benzylidene)-5-oxo-3-phenylthiazolidin-2-ylidene)-*N*-(*p*-tolyl)acetamide (**6c**) offered the highest binding affinity (− 8.1868 kcal/mol) when docked into (PDB ID:2ITO), in addition to 2-cyano-*N*-(4-(diethylamino)phenyl)-2-(4-(4-(diphenylamino)benzylidene)-5-oxo-3-phenylthiazolidin-2-ylidene)acetamide (**6a**) gave the highest energy score (− 9.3507 kcal/mol) with (PDB ID:2A4L).

**Supplementary Information:**

The online version contains supplementary material available at 10.1186/s13065-022-00879-x.

## Introduction

Carcinoma is described as the abnormal growth of cells and can penetrate tissues and destroy them [1, 2]. Though, tumor is a fatal disease and a major medical, psychological, and financial strain on individuals around the world [3]. However, it represents a public health problem and is called the world’s second leading cause of death after heart disease [4, 5]. The most commonly diagnosed types of cancer in 2018 are prostate, lung, breast, and melanoma cancer. In 2018, the number of new cases of cancer increased to 18.1 million, and deaths occurred at 9.6 million. Breast cancer is a serious disease consisting of heterogeneous tumors associated with a distinctive histological pattern and diverse clinical characteristics. The percentage of women sick with breast cancer in 2018 (11.6%) and in 2020 (26%) will increase from current levels [6, 7]. Lung carcinoma is also the most common type of cancer, leading to the death of men and women (11.6%). There are two main types of lung cancer: non-small and small-cell lung cancer [8]. It is proposed that, up to 1,898,160 cancer cases and 608,570 cancer deaths were reached to in 2021, in addition to 1,918,030 new cancer cases and 609,360 cancer deaths will be expected to occur, including nearly 350 deaths per day by lung cancer in 2022. [9, 10]. As well as, 327,000 men and 293,000 women will die in 2030 [11]. Over and above, new cancer cases are expected to increase to 29.5 million per year and cancer cases related deaths are projected to increase to 16.4 million in 2040 [12]. Meanwhile, the global encumbrance of lung cancer will be projected to reach 3.8 million new cases and 3.2 million deaths each year in 2050 [13]. Presently, there are more than 100 types of cancer, and each cancer type needs a diagnosis, so it has drawn the attention of scientists and chemists to develop new anticancer drugs with low toxicity, improved potency, and multiple mechanisms of action [14].

**Fig. 1:**
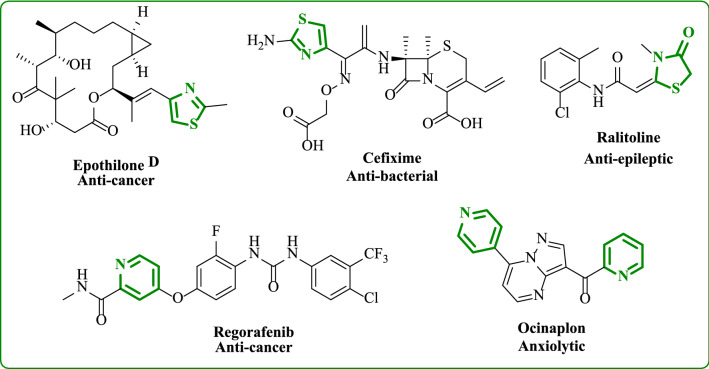
Drugs containing thiazole and/or pyridine ring systems

Heterocyclic compounds, which contain oxygen, sulfur, and nitrogen, play an important role in medicine, chemistry, and biological fields. Heterocycles are present in more than 85–95 percent of new medications, according to the literature. Among these compounds are pyridine, pyrazole, thiazole, and oxazole [15, 16]. Thiazole-containing heterocycles are fundamental scaffolds present in various natural and synthetic compounds. Thiazole substituted has a lot of interest due to its vast range of biological activity, such as anti-cancer [17–19], anti-bacterial [20], anti-inflammatory [21], anti-oxidant [22], and anti-epileptic [23]. On the other hand, the pyridine moiety also shows wide biological activities as anti-bacterial, anti-oxidant [24], and anti-cancer [25, 26]. Pyridine represents the skeletal backbone of many therapeutic agents [27] (Fig. 1). The aim of this research is to synthesize novel pyridine and thiazole compounds which are substituted with triphenylamine to allow comparison between them and study the biological effects of these compounds as anti-cancer agents against two cancer cell lines, breast and lung cancer cell lines. Docking studies were used to measure the effectiveness of the compounds to inhibit the anti-cancer activity.

## Experimental

### Materials and methods

The chemicals were purchased from Sigma-Aldrich and used without purification. A Nicolet iS10 spectrometer from Thermo Scientific was used to record IR spectra (KBr). The ^1^H NMR and ^13^C NMR spectra were recorded by JEOL's (500 MHz) and Bruker NMR spectrometers (400 MHz) in DMSO-*d*_*6*_ and CDCl_3_. Mass spectra was measured through Thermo Scientific GC/MS model ISQ. The Perkin-Elmer 2400 analyzer was used to obtain the elemental analysis of C, H, and N.

### Synthesis of *N*-aryl-2-cyano-3-(4-(diphenylamino)phenyl)acrylamide compounds 3a-e

In a dry 50 mL RB flask, each cyanoacetanilide derivative **2a-e** [namely; 2-cyano-*N*-phenylacetamide (0.96 g, 6 mmol), 2-cyano-*N*-(4-tolyl acetamide (1.04 g, 6 mmol), 2-cyano-*N*-(4-anisyl)acetamide (1.14 g, 6 mmol), 2-cyano-*N*-(4-(dimethylamino)phenyl)acetamide (1.22 g, 6 mmol), or 2-cyano-*N*-(4-nitrophenyl) acetamide (1.23 g, 6 mmol)] was added to a solution of 4-(diphenylamino)benzaldehyde (**1**) (1.64 g, 6 mmol) in 25 mL ethanol and 0.2 mL piperidine. The mixture was refluxed for 2 h and the solid that formed was collected and purified by recrystallization from EtOH to furnish the acrylamide compounds **3a-e**.

### 2-Cyano-3-(4-(diphenylamino)phenyl)-*N*-phenylacrylamide (3a)

Yellow crystals (83% yield); m.p. = 210–214 °C. IR (ῡ, cm^−1^): 3328 (N–H), 2210 (C≡N), 1679 cm^−1^ (C = O); ^1^H NMR (DMSO-*d*_6_): *δ* (ppm): 6.90 (d, *J* = 9.00 Hz, 2H, Ar–H), 7.11 (t, *J* = 7.50 Hz, 1H, Ar–H), 7.19–7.24 (m, 6H, Ar–H), 7.34 (t, *J* = 8.00 Hz, 2H, Ar–H), 7.42 (t, *J* = 8.00 Hz, 4H, Ar–H), 7.63 (d, *J* = 7.50 Hz, 2H, Ar–H), 7.88 (d, *J* = 8.50 Hz, 2H, Ar- H), 8.11 (s, 1H, CH = C), 10.19 (s, 1H, N–H); ^13^C NMR (DMSO-*d*_6_): *δ* (ppm): 101.90, 117.51, 118.54 (2C), 120.59 (2C), 123.35, 124.14, 125.53 (2C), 126.36 (4C), 128.71 (2C), 130.07 (4C), 132.32 (2C), 138.44, 145.42 (2C), 150.04, 151.27, 161.12; Mass analysis (m/z, %): 415 (M^+^, 100.00), 323 (43.27), 295 (11.28); Analysis for C_28_H_21_N_3_O (415.17): Calculated: C, 80.94; H, 5.09; N, 10.11%. Found: C, 80.80; H, 5.05; N, 10.19%.

### 2-Cyano-3-(4-(diphenylamino)phenyl)-*N*-(*p*-tolyl)acrylamide (3b)

Yellow crystals (60% yield); m.p. = 200–202 °C. IR (ῡ, cm^−1^): 3311 (N–H), 2221 (C≡N), 1670 cm^−1^ (C = O). ^1^H NMR (DMSO-*d*_6_): *δ* (ppm): 2.29 (s, 3H, CH_3_), 6.93 (d, *J* = 8.00 Hz, 2H, Ar–H), 7.17 (d, *J* = 8.00 Hz, 2H, Ar–H), 7.21–7.27 (m, 6H, Ar–H), 7.44 (t, *J* = 8.00 Hz, 4H, Ar–H), 7.56 (d, *J* = 8.00 Hz, 2H, Ar- H), 7.90 (d, *J* = 8.00 Hz, 2H, Ar–H), 8.15 (s, 1H, CH = C), 10.17 (s, 1H, N–H); ^13^C NMR (DMSO-*d*_6_): *δ* (ppm): 22.66, 102.50, 117.64, 119.07 (2C), 121.10 (2C), 123.90, 125.95 (2C), 126.77 (4C), 129.55 (2C), 130.52 (4C), 132.72 (2C), 133.63, 136.39, 145.91 (2C), 150.30, 151.67, 161.39; Mass analysis (m/z, %): 429 (M^+^, 17.93), 415 (48.51), 394 (20.07), 369 (19.93), 363 (20.76), 362 (21.72), 345 (23.63), 315 (24.84), 263 (23.38), 205 (100.00), 126 (25.96), 64 (22.74); Analysis for C_29_H_23_N_3_O (429.18): Calculated: C, 81.09; H, 5.40; N, 9.78%. Found: C, 81.25; H, 5.47; N, 9.69%.

### 2-Cyano-3-(4-(diphenylamino)phenyl)-*N*-(4-methoxyphenyl)acrylamide (3c)

Pale brown crystals (59% yield); m.p. = 180–182 °C. IR (ῡ, cm^−1^): 3321 (N–H), 2208 (C≡N), 1676 cm^−1^ (C = O); ^1^H NMR (DMSO-*d*_6_): *δ* (ppm): 3.75 (s, 3H, OCH_3_), 6.93 (t, *J* = 8.00 Hz, 4H, Ar–H), 7.21–7.26 (m, 6H, Ar–H), 7.44 (t,* J* = 8.00 Hz, 4H, Ar–H), 7.59 (d, *J* = 8.00 Hz, 2H, Ar–H), 7.90 (d, *J* = 8.00 Hz, 2H, Ar–H), 8.15 (s, 1H, CH = C), 10.13 (s, 1H, N–H); ^13^C NMR (DMSO-*d*_6_): *δ* (ppm): 55.65, 102.52, 114.24 (2C), 117.67, 119.11 (2C), 122.76 (2C), 123.98, 125.91 (2C), 126.74 (4C), 130.51 (4C), 131.94, 132.69 (2C), 145.93 (2C), 150.17, 151.61, 156.31, 161.23; Mass analysis (m/z, %): 445 (M^+^, 35.06), 443 (62.33), 426 (82.47), 398 (85.36), 339 (72.49), 308 (80.28), 294 (87.04), 277 (77.25), 275 (100.00), 241 (78.09), 156 (51.84), 51 (81.82); Analysis for C_29_H_23_N_3_O_2_ (445.18): Calculated: C, 78.18; H, 5.20; N, 9.43%. Found: C, 78.07; H, 5.25; N, 9.51%.

### 2-Cyano-*N*-(4-(dimethylamino)phenyl)-3-(4-(diphenylamino)phenyl)acrylamide (3d)

Red crystals (69% yield); m.p. = 220–222 °C. IR (ῡ, cm^−1^): 3324 (N–H), 2206 (C≡N), 1661 cm^−1^ (C = O); ^1^H NMR (DMSO-*d*_6_): *δ* (ppm): 2.88 (s, 6H, -N(CH_3_)_2_), 6.73 (d, *J* = 8.00 Hz, 2H, Ar–H), 6.93 (d, *J* = 8.00 Hz, 2H, Ar–H), 7.21–2.26 (m, 6H, Ar–H), 7.42–7.50 (m, 6H, Ar–H), 7.89 (d, *J* = 12.00 Hz, 2H, Ar–H), 8.11 (s, 1H, CH = C), 9.95 (s, 1H, N–H); ^13^C NMR (DMSO-*d*_6_): *δ* (ppm): 40.84 (2C), 102.76, 112.81 (2C), 117.75, 119.19 (2C), 122.54 (2C), 124.11, 125.87 (2C), 126.70 (4C), 128.40, 130.50 (4C), 132.60 (2C), 145.97 (2C), 148.02, 149.81, 151.51, 160.80; Mass analysis (m/z, %): 458 (M^+^, 16.91), 439 (85.53), 413 (31.68), 411 (33.49), 394 (62.28), 384 (35.46), 300 (47.98), 293 (39.32), 288 (40.80), 254 (57.49), 246 (100.00), 167 (35.94), 121 (55.43), 42 (32.64); Analysis for C_30_H_26_N_4_O (458.21): Calculated: C, 78.58; H, 5.72; N, 12.22%. Found: C, 78.48; H, 5.75; N, 12.29%.

### 2-Cyano-3-(4-(diphenylamino)phenyl)-*N*-(4-nitrophenyl)acrylamide (3e)

Orange crystals (70% yield); m.p. = 248–250 °C. IR (ῡ, cm^−1^): 3314 (N–H), 2215 (C≡N), 1683 cm^−1^ (C = O); ^1^H NMR (DMSO-*d*_6_): *δ* (ppm): 6.90 (d, *J* = 9.00 Hz, 2H, Ar–H), 7.21–7.26 (m, 6H, Ar–H), 7.41–7.44 (m, 4H, Ar–H), 7.89–7.93 (m, 4H, Ar–H), 8.17 (s, 1H, CH = C), 8.25 (d, *J* = 9.00 Hz, 2H, Ar–H), 10.75 (s, 1H, N–H); ^13^C NMR (DMSO-*d*_6_): *δ* (ppm): 101.23, 116.97, 118.32 (2C), 120.15 (2C), 123.05, 124.81 (2C), 125.69, 126.48 (4C), 130.11 (4C), 132.63 (2C), 142.65, 145.06 (2C), 145.29 (2C), 150.97, 151.58, 162.08; Mass analysis (m/z, %): 460 (M^+^, 100.00), 323 (83.91), 295(89.71), 77 (35.70); Analysis for C_28_H_20_N_4_O_3_ (460.15): Calculated: C, 73.03; H, 4.38; N, 12.17%. Found: C, 73.15; H, 4.31; N, 12.07%.

### General synthesis of 4-(4-(diphenylamino)phenyl)-2-pyridone 4a-e

In dry 50 mL RB flask, malononitrile (0.33 g, 5 mmol) was added to a solution of acrylamide derivatives **(3a-e)**, 5 mmol of each 2-cyano-3-(4-(diphenylamino)phenyl)-*N*-phenylacrylamide (2.00 g, 5 mmol), (2-cyano-3-(4-(diphenylamino)phenyl)-*N*-(4-nitrophenyl)acrylamide (2.30 g, 5 mmol), 2-cyano-3-(4-(diphenylamino)phenyl)-*N*-(p-tolyl)acrylamide (2.14 g, 5 mmol), 2-cyano-3-(4-(diphenylamino)phenyl)-*N*-(4-methoxyphenyl)acrylamide (2.22 g, 5 mmol), and 2-cyano-*N*-(4-(dimethylamino)phenyl)-3-(4-(diphenylamino)phenyl)acrylamide (2.29 g, 5 mmol), respectively in 40 mL dry ethanol and 0.3 mL piperidine. Reflux the reaction mixture for 6 h. The solid so formed was collected and purified by recrystallization from EtOH to produce pyridone compounds **4a-e**.

### 6-Amino-4-(4-(diphenylamino)phenyl)-2-oxo-1-phenyl-1,2-dihydropyridine-3,5-dicarbonitrile (4a)

Yellow crystals (35% yield); m.p. above 300 °C. IR (ῡ, cm^−1^): 3303, 3202 (NH_2_), 2216 (C≡N), 1667 (C = O); ^1^H NMR (DMSO-*d*_6_): *δ* (ppm): 6.95 (d, *J* = 8.00 Hz, 2H, Ar–H), 7.15–7.18 (m, 6H, Ar–H), 7.34 (d, *J* = 8.00 Hz, 2H, Ar–H), 7.38–7.42 (m, 6H, Ar–H), 7.51 (d, *J* = 7.00 Hz, 1H, Ar–H), 7.55 (d, *J* = 8.00 Hz, 2H, Ar–H); ^13^C NMR (DMSO-*d*_6_): *δ* (ppm): 75.02, 116.17, 116.86, 119.25 (2C), 124.68 (2C), 125.76 (5C), 126.32, 128.64 (2C), 129.63 (2C), 129.98 (5C), 130.36 (2C), 133.98, 146.21 (2C), 149.25, 157.21, 159.81, 160.71; Mass analysis (m/z, %): 479 (M^+^, 14.88), 452 (44.84), 449 (48.59), 446 (51.78), 435 (52.92), 415 (70.57), 392 (80.01), 389 (40.71), 363 (54.21), 358 (72.86), 252 (69.21), 216 (40.90), 212 (82.76), 190 (44.14), 163 (40.42), 149 (60.53), 110 (41.12), 66 (100.00); Analysis for C_31_H_21_N_5_O (479.17): Calculated: C, 77.64; H, 4.41; N, 14.60%. Found: C, 77.53; H, 4.38; N, 14.66%.

### 6-Amino-4-(4-(diphenylamino)phenyl)-2-oxo-1-(*p*-tolyl)-1,2-dihydropyridine-3,5-dicarbonitrile (4b)

Pale orange crystals (49% yield); m.p. above 300 °C. IR (ῡ, cm^−1^): 3293, 3210 (NH_2_), 2216 (C≡N), 1663 (C = O); ^1^H NMR (DMSO-*d*_6_): *δ* (ppm): 2.37 (s, 3H, CH_3_), 6.95 (d, *J* = 9.00 Hz, 2H, Ar–H), 7.15–7.18 (m, 6H, Ar–H), 7.22 (d, *J* = 9.00 Hz, 2H, Ar–H), 7.35–7.43 (m, 8H, Ar–H); ^13^C NMR (DMSO-*d*_6_): *δ* (ppm): 20.89, 74.96, 116.12, 116.84, 119.31 (2C), 124.62 (2C), 125.67 (4C), 126.39, 128.27 (2C), 129.58 (2C), 129.93 (4C), 130.84 (2C), 131.27, 139.34, 146.20 (2C), 149.21, 157.27, 158.59, 159.80, 160.58; Mass analysis (m/z, %): 493 (M^+^, 15.10), 461 (16.18), 447 (16.22), 415 (16.79), 334 (29.61), 231 (33.82), 196 (78.53), 195 (100.00), 161 (32.51), 119 (19.90), 95 (20.23); Analysis for C_32_H_23_N_5_O (493.19): Calculated: C, 77.87; H, 4.70; N, 14.19%. Found: C, 77.75; H, 4.74; N, 14.27%.

### 6-Amino-1-(*p*-anisyl)-4-(4-(diphenylamino)phenyl)-2-oxo-1,2-dihydropyridine-3,5-dicarbonitrile (4c)

Greenish yellow crystals (38% yield); m.p. above 300 °C. IR (ῡ, cm^−1^): 3295, 3205 (NH_2_), 2217 (C≡N), 1665 (C = O); ^1^H NMR (DMSO-*d*_6_): *δ* (ppm): 3.81 (s, 3H, OCH_3_), 6.95 (d, *J* = 8.50 Hz, 2H, Ar–H), 7.09 (d, *J* = 8.50 Hz, 2H, Ar–H), 7.15–7.18 (m, 6H, Ar–H), 7.26 (d, *J* = 8.50 Hz, 2H, Ar–H), 7.38–7.42 (m, 6H, Ar–H); ^13^C NMR (DMSO-*d*_6_): *δ* (ppm): 56.01, 74.98, 115.54 (2C), 116.21, 116.93, 119.34 (2C), 124.62 (2C), 125.67 (4C), 126.29, 126.47, 129.57 (2C), 129.78 (2C), 129.93 (4C), 146.22 (2C), 149.19, 157.56, 158.76, 160.00, 160.05, 160.51; Mass analysis (m/z, %): 509 (M^+^, 30.08), 462 (38.83), 349 (32.89), 343 (31.80), 328 (32.71), 307 (41.16), 257 (57.56), 236 (37.56), 225 (40.40), 171 (64.85), 167 (53.50), 117 (78.23), 109 (39.67), 95 (100.00); Analysis for C_32_H_23_N_5_O_2_ (509.19): Calculated: C, 75.43; H, 4.55; N, 13.74%. Found: C, 75.63; H, 4.46; N, 13.63%.

### 6-Amino-1-(4-(dimethylamino)phenyl)-4-(4-(diphenylamino)phenyl)-2-oxo-1,2-dihydropyridine-3,5-dicarbonitrile (4d)

Green crystals (32% yield); m.p. = above 300 °C. IR (ῡ, cm^−1^): 3296, 3210 (NH_2_), 2216 (C≡N), 1663 (C = O; ^1^H NMR (DMSO-*d*_6_): *δ* (ppm): 2.96 (s, 6H, -N(CH_3_)_2_), 6.82 (d, *J* = 9.00 Hz, 2H, Ar–H), 6.95 (d, *J* = 8.00 Hz, 2H, Ar–H), 7.09 (d, *J* = 8.50 Hz, 2H, Ar–H), 7.14–7.18 (m, 6H, Ar–H), 7.38–7.42 (m, 6H, Ar–H); ^13^C NMR (DMSO-*d*_6_): *δ* (ppm): 39.80 (2C), 74.73, 87.43, 113.17 (2C), 116.21, 116.99, 119.36 (2C), 121.50, 124.64 (2C), 125.70 (4C), 126.46, 128.78 (2C), 129.62 (2C), 129.97 (4C), 146.24 (2C), 149.21, 150.92, 157.78, 160.15, 160.37; Mass analysis (m/z, %): 522 (M^+^, 18.71), 492 (40. 92), 405 (66.96), 355 (29.47), 266 (76.63), 265 (94.28), 249 (57.20), 146 (66.79), 117 (64.50), 111 (62.47), 110 (100.00), 75 (52.12), 65 (51.85), 64 (72.14), 51 (69.29); Analysis for C_33_H_26_N_6_O (522.22): Calculated: C, 75.84; H, 5.01; N, 16.08%. Found: C, 75.97; H, 5.05; N, 16.17%.

### 6-Amino-4-(4-(diphenylamino)phenyl)-1-(4-nitrophenyl)-2-oxo-1,2-dihydropyridine-3,5-dicarbonitrile (4e)

Yellow crystals (37% yield); m.p. above 300 °C. IR (ῡ, cm^−1^): 3301, 3210 (NH_2_), 2224 (C≡N), 1686 (C = O); ^1^H NMR (DMSO-*d*_6_): *δ* (ppm): 6.95 (d, *J* = 9.00 Hz, 2H, Ar–H), 7.15–7.17 (m, 6H, Ar–H), 7.39 (t, *J* = 9.50 H, 6H, Ar–H), 7.67 (d, *J* = 8.00 Hz, 2H, Ar–H), 8.38 (d, *J* = 9.00 Hz, 2H, Ar–H); ^13^C NMR (DMSO-*d*_6_): *δ* (ppm): 116.30, 116.89, 119.25 (2C), 124.71 (2C), 125.59 (2C), 125.79 (5C), 126.36, 129.58 (2C), 129.99 (5C), 130.89 (2C), 146.21 (2C), 148.37, 149.28, 157.11, 158.24, 159.77, 160.85; Mass analysis (m/z, %): 524 (M^+^, 21.25), 499 (53.60), 447 (100.00), 439 (44.34), 413 (41.80), 315 (46.48), 222 (66.87), 212 (55.13), 167 (54.16), 106 (70.96), 86 (42.66), 66 (71.22), 40 (97.12); Analysis for C_31_H_20_N_6_O_3_ (524.16): Calculated: C, 70.98; H, 3.84; N, 16.02%. Found: C, 70.78; H, 3.92; N, 16.12%.

### Synthesis 4-(diphenylamino)benzylidene)thiazolidin-5-one derivatives 6a-d

4-(Diphenylamino)benzaldehyde (**1**) (0.55 g, 2 mmol) was dissolved in 30 mL absolute ethanol in a 50 mL RB flask. Then, each thiazolidin-4-one derivative **5a**, **5b**, **5c** or **5d** (2 mmol) [namely; 2-cyano-*N*-(4-(diethylamino)phenyl)-2-(5-oxo-3-phenylthiazolidin-2-ylidene)acetamide (0.81 g), 2-cyano-*N*-(4-(dimethylamino)-phenyl)-2-(5-oxo-3-phenylthiazolidin-2-ylidene)acetamide (0.75 g), 2-cyano-2-(5-oxo-3-phenylthiazolidin-2-ylidene)-*N*-(*p*-tolyl)acetamide (0.69 g), or 2-cyano-*N*-(*p*-anisyl)-2-(5-oxo-3-phenyl-thiazolidin-2-ylidene)acetamide (0.73 g)] and 0.1 mL piperidine were added to the reaction mixture. The mixture has been refluxed for 3 h, the solid product formed on hot was filtered off and washed with boiling ethanol to yield compounds **6a-d**.

### 2-Cyano-*N*-(4-(diethylamino)phenyl)-2-(4-(4-(diphenylamino)benzylidene)-5-oxo-3-phenylthiazolidin-2-ylidene)acetamide (6a)

Red crystals (49% yield); m.p. above 300 °C. IR (ῡ, cm^−1^): 3392 (N–H), 2191 (C≡N), 1708 (C = O), 1644 (C = O); ^1^H NMR (DMSO-d_6_): *δ* (ppm): 1.04 (t, *J* = 7.00 Hz, 6H, 2 -CH_3_), 3.26 (q, *J* = 7.00 Hz, 4H, 2 -NCH_2_-), 6.56 (d, *J* = 9.00 Hz, 2H, Ar–H), 6.97 (d, *J* = 9.00 Hz, 2H, Ar–H), 7.15–7.20 (m, 6H, Ar–H), 7.26 (d, *J* = 9.00 Hz, 2H, Ar–H), 7.39 (t, *J* = 8.00 Hz, 4H, Ar–H), 7.53–7.56 (m, 5H, Ar–H), 7.59 (d, *J* = 8.50 Hz, 2H, Ar–H), 7.66 (s, 1H, CH = C), 9.22 (s, 1H, NH); Mass analysis (m/z, %): 661 (M^+^, 10.63), 617 (33.03), 550 (26.07), 415 (19.40), 341(54.99), 312 (55.83), 300 (45.13), 219 (58.14), 196 (46.16), 168 (40.00), 77 (36.23), 53 (97.54), 43 (100.00); Analysis for C_41_H_35_N_5_O_2_S (661.25): Calculated: C, 74.41; H, 5.33; N, 10.58%. Found: C, 74.58; H, 5.27; N, 10.50%.

### 2-Cyano-*N*-(4-(dimethylamino)phenyl)-2-(4-(4-(diphenylamino)benzylidene)-5-oxo-3-phenylthiazolidin-2-ylidene)acetamide (6b)

Brown crystals (42% yield); m.p. above 300 °C. IR (ῡ, cm^−1^): 3402 (N–H), 2191 (C≡N), 1710 (C = O), 1649 (C = O); ^1^H NMR (DMSO-d_6_): *δ* (ppm): 2.83 (s, 6H, -N(CH_3_)_2_), 6.64 (d, *J* = 9.00 Hz, 2H, Ar–H), 6.97 (d, *J* = 9.00 Hz, 2H, Ar–H), 7.15–7.20 (m, 6H, Ar–H), 7.31 (d, *J* = 9.00 Hz, 2H, Ar–H), 7.39 (t, *J* = 8.00 Hz, 4H, Ar–H), 7.53–7.57 (m, 5H, Ar–H), 7.59 (d, *J* = 8.50 Hz, 2H, Ar–H), 7.67 (s, 1H, CH = C), 9.27 (s, 1H, NH); Mass analysis (m/z, %): 633 (M^+^, 8.92), 560 (28.54), 484 (19.29), 410 (16.26), 311 (10.25), 242 (100.00), 132 (36.36), 76 (98.42), 70 (36.46), 68 (44.05), 42 (80.68), 41 (45.66); Analysis for C_39_H_31_N_5_O_2_S (633.22): Calculated: C, 73.91; H, 4.93; N, 11.05%. Found: C, 73.80; H, 4.99; N, 11.14%.

### 2-Cyano-2-(4-(4-(diphenylamino)benzylidene)-5-oxo-3-phenylthiazolidin-2-ylidene)-*N*-(*p*-tolyl)acetamide (6c)

Orange crystals (40% yield); m.p. above 300 °C. IR (ῡ, cm^−1^): 3409 (N–H), 2187 (C≡N), 1721 (C = O), 1652 (C = O); ^1^H NMR (DMSO-d_6_): *δ* (ppm): 2.24 (s, 3H, CH_3_), 6.98 (d, *J* = 9.00 Hz, 2H, Ar–H), 7.08 (d, *J* = 8.50, 2H, Ar–H), 7.16–7.21 (m, 6H, Ar–H), 7.40 (t, *J* = 9.00 Hz, 6H, Ar–H), 7.54–7.56 (m, 5H, Ar–H), 7.60 (d, *J* = 8.50, 2H, Ar–H), 7.69 (s, 1H, CH = C), 9.48 (s, 1H, NH); Mass analysis (m/z, %): 604 (M^+^, 21.21), 564 (42.39), 499 (42.03), 398 (95.49), 308 (40.03), 274 (64.01), 200 (36.67), 195 (81.36), 149 (40.12), 91 (52.48), 90 (100.00), 65 (47.45), 61 (97.64); Analysis for C_38_H_28_N_4_O_2_S (604.19): Calculated: C, 75.47; H, 4.67; N, 9.26%. Found: C, 75.63; H, 4.60; N, 9.15%.

### 2-Cyano-2-(4-(4-(diphenylamino)benzylidene)-5-oxo-3-phenylthiazolidin-2-ylidene)-*N*-(4-methoxyphenyl)acetamide (6d)

Orange crystals (45% yield); m.p. above 300 °C. IR (ῡ, cm^−1^): 3408 (N–H), 2188 (C≡N), 1719 (C = O), 1648 (C = O); ^1^H NMR (DMSO-d_6_): *δ* (ppm): 3.70 (s, 3H, -OCH_3_), 6.84 (d, *J* = 8.50 Hz, 2H, Ar–H), 6.97 (d, *J* = 9.00 Hz, 2H, Ar–H), 7.15–7.20 (m, 6H, Ar–H), 7.38–7.42 (m, 7H, Ar–H), 7.54–7.56 (m, 4H, Ar–H), 7.59 (d, *J* = 8.50 Hz, 2H, Ar–H), 7.68 (s, 1H, CH = C), 9.45 (s, 1H, N–H); Mass analysis (m/z, %): 620 (M^+^, 34.41), 602 (43.43), 529 (86.94), 512 (43.51), 442 (43.21), 431 (76.80), 382 (97.21), 323 (99.83), 305 (100.00), 265 (52.71), 251 (51.59), 216 (46.39), 177 (51.42), 121 (47.77), 77 (64.00); Analysis for C_38_H_28_N_4_O_3_S (620.19): Calculated: C, 73.53; H, 4.55; N, 9.03%. Found: C, 73.66; H, 4.51; N, 9.11%.

### Synthesis of ethyl-2-(5-(4-(diphenylamino)benzylidene)-4-oxo-3-phenylthiazolidin-2-ylidene)acetate (8)

A dry 50 mL RB flask was charged with a solution of ethyl 2-(4-oxo-3-phenylthiazolidin-2-ylidene)acetate (**7a**) (0.52 g, 2 mmol) in 30 mL of absolute ethanol. To this solution, 4-(diphenylamino*)*benzaldehyde (0.54 g, 2 mmol) and 0.1 mL piperidine were added. The mixture was refluxed for 2 h and the solid so formed upon cooling was collected and recrystallized from ethanol.

Orange crystals (53% yield); m.p. = 250–252 °C. IR (ῡ, cm^−1^): 1705 (C = O), 1669 (C = O); ^1^H NMR (CDCl_3_): *δ* (ppm): 1.24 (t, *J* = 7.50 Hz, 3H, CH_3_), 4.16 (q, *J* = 7.50 Hz, 2H, -OCH_2_-), 5.22 (s, 1H, CH = C), 7.06 (d, *J* = 9.00 Hz, 2H, Ar–H), 7.11–7.17 (m, 5H, Ar–H), 7.28–7.33 (m, 7H, Ar–H), 7.48–7.57 (m, 5H, Ar–H), 7.67 (s, 1H, CH = C). ^13^C NMR (CDCl_3_): *δ* (ppm): 14.84, 60.18, 92.27, 118.45 (2C), 120.40, 121.73, 123.73, 125.01 (2C), 126.26, 126.37, 126.50, 127.45, 128.97, 129.41, 129.48, 130.13, 130.20, 130.76, 131.15, 132.41, 135.08, 135.16, 146.52, 146.60, 149.25, 149.32, 154.24 (2C), 166.77, 167.46; Mass analysis (m/z, %): 518 (M^+^, 19.01), 458 (31.32), 429 (86.34), 422 (31.15), 318 (40.00), 269 (100.00), 252 (30.06), 211 (32.51), 207 (30.11), 189 (56.37), 182 (31.13), 129 (32.31), 98 (22.23); Analysis for C_32_H_26_N_2_O_3_S (518.17): Calculated: C, 74.11; H, 5.05; N, 5.40%. Found: C, 74.01; H, 5.07; N, 5.36%.

### General synthesis of compounds 9 and 10

4-(Diphenylamino)benzaldehyde (**1**) (1.09 g, 4 mmol) was dissolved in 20 mL glacial acetic acid in a 50 mL RB flask. Then, a solution of 5-methyl-2-phenyl-2,4-dihydro-3H-pyrazol-3-one (**7b**) (0.70 g, 4 mmol) or 3-phenylisoxazol-5(4H)-one (**7c**) (0.64 g, 4 mmol) and ammonium acetate (0.62 g, 8 mmol) were added. The reaction mixture was subjected to reflux for 3 h. The mixture was cooled at room temperature and poured into ice water. The crude product was purified by recrystallization from EtOH to produce compounds **9** and **10**.

### 4-(4-(Diphenylamino)benzylidene)-5-methyl-2-phenyl-2,4-dihydro-3*H*-pyrazol-3-one (9)

Red crystals (55% yield); m.p. = 182–184 °C. IR (ῡ, cm^−1^): 1675 (C = O); ^1^H NMR (DMSO-*d*_6_): *δ* (ppm): 2.28 (s, 3H, CH_3_), 6.82 (d, *J* = 9.00 Hz, 2H, Ar–H), 7.14 (t, *J* = 7.00 Hz, 1H, Ar–H), 7.22–7.27 (m, 6H, Ar–H), 7.37–7.44 (m, 6H, Ar–H), 7.60 (s, 1H, CH = C), 7.89 (d, *J* = 7.50, 2H, Ar–H), 8.54 (d, *J* = 8.50 Hz, 2H, Ar–H); ^13^C NMR (DMSO-*d*_6_): *δ* (ppm): 13.14, 117.35 (2C), 118.19 (2C), 122.27, 124.25, 125.08, 125.90 (2C), 126.63 (4C), 128.76 (2C), 130.08 (4C), 136.47 (2C), 138.48, 145.10 (2C), 147.56, 151.69, 152.07, 161.96; Mass analysis (m/z, %): 429 (M^+^, 36.25), 411 (40.58), 379 (100.00), 347 (87.40), 306 (69.08), 237 (45.39), 161 (84.84), 104 (52.45), 67 (96.32), 53 (82.03); Analysis for C_29_H_23_N_3_O (429.18): Calculated: C, 81.09; H, 5.40; N, 9.78%. Found: C, 81.20; H, 5.37; N, 9.85%.

### 4-(4-(Diphenylamino)benzylidene)-3-phenylisoxazol-5(4*H*)-one (10)

Reddish orange crystals (68% yield); m.p. = 218–220 °C. IR (ῡ, cm^−1^): 1739 (C = O); ^1^H NMR (CDCl_3_): *δ* (ppm): 6.94 (d, *J* = 9.00 Hz, 2H, Ar–H), 7.19–7.24 (m, 6H, Ar–H), 7.37 (t, *J* = 8.00 Hz, 4H, Ar–H), 7.40 (s, 1H, CH = C), 7.53–7.59 (m, 5H, Ar–H), 8.26 (d, *J* = 8.50 Hz, 2H, Ar–H); ^13^C NMR (CDCl_3_): *δ* (ppm): 112.87, 118.20 (2C), 124.95, 126.00 (2C), 126.31, 126.71 (4C), 128.16, 128.78 (2C), 129.07 (2C), 129.84 (4C), 130.52, 136.88 (2C), 145.15, 151.30, 153.40, 164.47, 169.58; Mass analysis (m/z, %): 416 (M^+^, 26.21), 396 (33.54), 359 (35.79), 300 (81.58), 226 (52.38), 205 (39.52), 145 (46.84), 105 (100.00), 93 (55.73), 58 (57.24). Analysis for C_28_H_20_N_2_O_2_ (416.15): Calculated: C, 80.75; H, 4.84; N, 6.73%. Found: C, 80.55; H, 4.92; N, 6.84%.

## Biological activity assays

### Anticancer screening

#### Cell cultures

The lung cancer **A-549** and the breast cancer **MDA-MB-231** cell lines were obtained from the viscera Tissue Culture Unit. The cells were spread in Dulbecco’s modified Eagle’s medium (DMEM) supplemented with 10% heat-inactivated fetal bovine serum, 1% L-glutamine, HEPES buffer, and 50 µg/mL gentamycin. All cells were maintained at 37ºC in a humidified atmosphere with 5% CO_2_ and were sub-cultured two times a week.

#### Cytotoxicity evaluation using viability assay

The MTT assay [28, 29] was applied to determine the cytotoxicity of pyridine and thiazole derivatives. MTT or 3-(4,5-dimethylthiazol-2-yl)-2,5-diphenyltetrazolium bromide was purchased from Serva, Germany. The cell lines were introduced into 96-well plates at a cell concentration of 1 × 10^4^ cells per well in 100 μL of complete medium (tests were done in duplicates). These plates were incubated for 24 h, 5% CO_2_, at 37 °C for settle down and adhesion. Control cells were incubated without the test sample and with or without DMSO. After incubation, different concentrations of samples were added after the period of incubation (48 h), media were inhaled, and the MTT solution was added to all wells for full 30 min. Briefly, the media was extracted from the 96 well plates and exchanged with 100 µl of fresh culture RPMI 1640 medium without phenol red then 10 µl of the 12 mM MTT stock solution (5 mg of MTT in 1 mL of PBS) to all wells inclusive the untreated controls. The 96 well plates were incubated at 37 °C and 5% CO_2_ for 4 h. An 85 µl aliquot of the media was taken away from the wells, added 50 µl of DMSO to all wells and mixed thoroughly, and incubated at 37 °C for 10 min. The absorbance of plates was measured after shaking on a microplate reader (TECAN, Inc.), using a test wavelength of 590 nm. Measure the optical density by the microplate reader to calculate the number of viable cells and the percentage of viability according to [(ODt/ODc)] × 100% where ODt (optical density of cells treated) with the tested sample and ODc (optical density of control cells).

#### Cytotoxicity evaluation against normal cells using viability assay

The MTT assay [28, 29] was applied to determine the cytotoxicity of pyridine and thiazole derivatives against normal cell line to explore the selectivity of the tested compounds against tumor cells. The cytotoxicity was tested against human lung fibroblast noncancerous cell line (MRC-5).

### Molecular docking study

Docking is a supporting technique for explanation the interaction between a ligand and the suitable active sites of the protein. Docking studies and calculations were applied via the program Molecular Operating Environment MOE v. 2019.0102. As EGFR is considered as an epidermal growth factor receptor, the crystal structure of the protein (PDB Code-2ITO) is recommended for EGFR and specific for A-549 (lung cell line). Also, crystal structure of EGFR kinase domain G719S mutation in complex with Iressa (PDB ID: 2ITO) [30, 31]. As well as, the protein structure file (PDB ID: 2A4L) which is related to the breast cancer cell line was downloaded from a protein data bank [7].

The docking requires several processes as the following: (1) removal of water and heteroatoms from the target complex, (2) the protein builder was carried out through add missing hydrogenes and automatic connect and type through fixation the potential energy, and (3) the docking process was performed. The docking procedure was done in a grid box dimensions of 10 Å in the x, y and z directions centered on the ligand using MOE. The molecular grid of the protein (2ITO) was kept around Glu 697, Ala 698, Pro 699, Asa 700, Lys 757, Glu 758, Asp 761, Glu 762, Tyr 764, Val 765, SER 768, Asp 830, Arg 831, Arg 832, Leu 833, Val 834, Arg 836, Asp 837, Phe 856, Gly 857, Leu 858, Ala 859, Lys 860, Leu 861, Leu 862, Gly 863, Ala 864, Glu 865, Glu 866, Val 876, Pro 877, Trp 880, Met 881, Ala 882, Ser 885, His 888, Arg 889, Ile 890, Tyr 891, Thr 892, Ser 895, Asp 896, Ser 899 [32].

## Results and discussion

### Chemistry

**Scheme 1:**
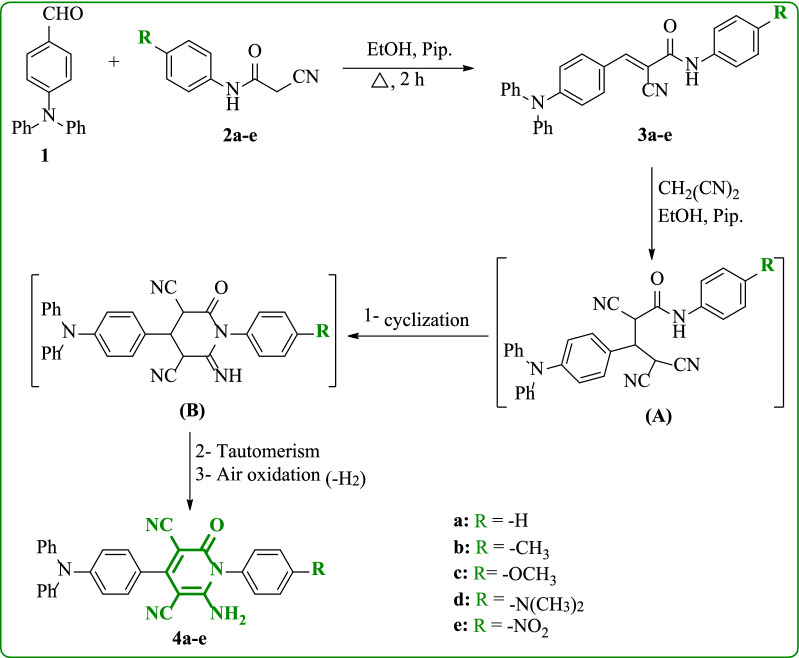
Synthesis of (4-(diphenylamino)phenyl)-2-pyridone derivatives (**4a-e**)

The synthetic pathway of 2-pyridone derivatives **4a-e** containing triphenylamine moiety was shown in Scheme 1. Firstly, synthesis of 4-(diphenylamino)benzaldehyde (**1**) in good yield by formylation of triphenylamine was achieved according to the modified conditions of Vilsmeier reaction [33]. The measured melting point and the spectral data of the 4-(diphenylamino)benzaldehyde (**1**) was in agreement with the literature [34]. Moreover, cyanoacetanilide derivatives **2a-e** were produced by refluxing different primary aromatic amines via aniline, *p*-toluidine, *p*-anisidine, *N*,*N*-dimethylbenzene-1,4-diamine, and 4-nitroaniline with 1-cyanoacetyl-3,5-dimethylpyrazole in a dioxane [35]. Thus, Knoevenagel condensation of 4-(diphenylamino)benzaldehyde (**1**) with the cyanoacetanilide derivatives **2a-e** in dry ethanol and piperidine produced the *N*-aryl-2-cyano-3-(4-(diphenylamino)phenyl)acrylamides **3a-e**. Finally, The targeted (4-(diphenylamino)phenyl)-2-pyridone analogues **4a-e** were prepared by the reaction of cyanoacrylamides **3a-e** with malononitrile in ethanol and drops of piperidine. The chemical structure of synthesized compounds **4a-e** was ensured based upon distinct spectral analyses. The structure of **4b** was established by IR data that showed absorptions of NH_2_ at 3293 and 3210 cm^−1^. The absorptions of nitrile (C≡N) and carbonyl (C = O) groups are observed at 2216 and 1663 cm^−1^. The ^1^H NMR spectrum exhibited a characteristic singlet for three protons of (CH_3_) at 2.37 ppm. The protons of an aromatic system in the range from δ 6.95 to 7.42 ppm were observed as doublet and multiplet signals. In addition, ^13^C NMR spectrum showed 20 carbon signals for thirty-two carbon atoms, the signal distinct at 20.89 ppm for methyl group. Furthermore, the mass spectrum displayed a molecular ion peak at *m/z* = 493 (15.10%) corresponding to the molecular weight of its chemical formula (C_32_H_23_N_5_O). The assignment of compound **4d** was based on its spectral analysis. The IR spectra exhibited absorption bands of amino group at 3296 and 3210 cm^−^1, nitrile group at 2216 cm^−^1, and carbonyl group at 1663 cm^−1^. Its ^1^H NMR spectrum showed a singlet at *δ* 2.96 ppm for six protons of two methyl groups.

**Scheme 2:**
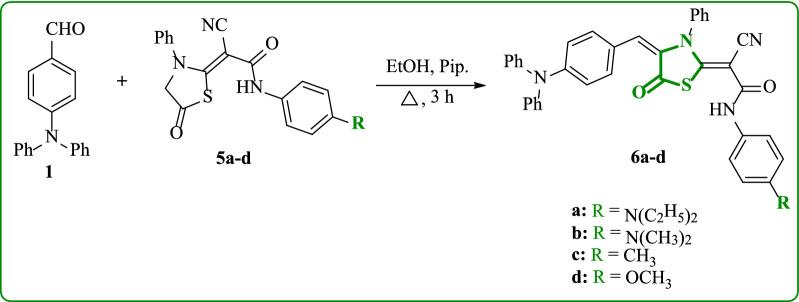
Synthesis of 4-(diphenylamino)benzylidene)thiazolidin-5-one analogues **6a-d**

The synthesis of new 4-(diphenylamino)benzylidene)-thiazolidin-5-one analogues **6a-d** was shown in Scheme 2. The synthesis of thiazolidine-5-one derivatives **5a-d**, The active methylene group of 2-((4-(2-cyanoacetamido)phenyl)(ethyl)amino)ethan-1-ylium, 2-cyano-*N*-(4-(dimethylamino)phenyl)acetamide, 2-cyano-*N*-(*p*-tolyl)acetamide, 2-cyano-*N*-(4-methoxyphenyl)acetamide was readily added to phenyl isothiocyanate in presence of alkaline medium (KOH) to form the non-isolable sulfide salt, which underwent heterocyclization upon treatment with chloroacetyl chloride to afford the corresponding thiazolidine-5-one derivatives **5a-d**, respectively [36]. The target compounds **6a-d** were synthesized using the Knoevenagel condensation reaction of 4-(diphenylamino)benzaldehyde (**1**) with thiazolidine-5-one derivatives **5a-d**, using absolute ethanol as a solvent and a catalytic amount of piperidine. The structures of these compounds **6a-d** were proven by elemental and spectroscopic data. The skeleton of compound **6a** (as an example) was confirmed by distinctive absorption bands from the IR spectrum, N–H stretching frequency at 3392 cm^−1^, nitrile (C≡N) at 2191 cm^−1^, carbonyl (C = O) of thiazolidine ring at 1708 cm^−1^, and amidic carbonyl at 1644 cm^−1^. The ^1^H NMR spectrum of **6a** offered triplet at δ 1.04 ppm for six protons of two methyl groups, quartet for four protons of (2CH_2_) at δ 3.26 ppm, singlet at δ 7.66 ppm attributed to olefinic proton, and singlet for N–H proton at 9.22 ppm, in addition, its mass spectrum displayed molecular ion peak at *m/z* = 661 (10.63%), corresponding to a molecular formula C_41_H_35_N_5_O_2_S.

**Scheme 3:**
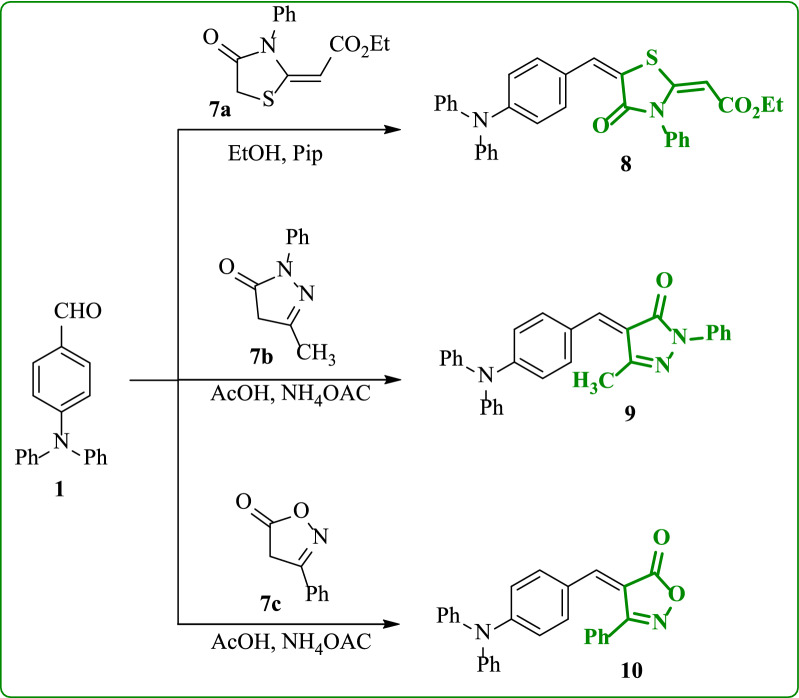
Synthesis of (4-(diphenylamino)benzylidene)-thiazolidinone / -pyrazolone / -isoxazolone analogues **8**, **9**, and **10**

The synthesis of thiazoldin-4-one compound **7a** was achieved through the addition of phenyl isothiocyanate to the active methylene of ethyl acetoacetate in sodium ethoxide followed by cyclization of the produced thiocarbamoyl derivative with ethyl bromoacetate as described in the literature [37]. 4-(Diphenylamino)benzaldehyde (**1**) was condensed with ethyl-2-(4-oxo-3-phenylthiazolidin-2-ylidene)acetate (**7a**) in absolute ethanol and piperidine as catalytically base to produce the corresponding 4-(diphenylamino)benzylidene)thiazolidin-4-one derivative **8** (Scheme 3). 3-Phenylisoxazol-5-one (**7c**) was obtained by condensation of ethylbenzoylacetate with hydroxylamine according to literature [38]. Moreover, condensation of 4-(diphenylamino)benzaldehyde (**1**) with 5-methyl-2-phenyl-2,4-dihydro-3*H*-pyrazol-3-one (**7b**) and/or 3-phenylisoxazol-5(4*H*)-one (**7c**) was carried out in refluxing acetic acid in the presence ammonium acetate to produce the corresponding (4-(diphenylamino)benzylidene)pyrazolone **9** and (4-(diphenylamino)benzylidene)-isoxazolone **10** analogues (Scheme 3). The structure of compound **8** (as an example) was elucidated by IR spectrum, which showed two distinctive absorption bands for carbonyl groups at 1705 and 1669 cm^−1^. The ^1^H NMR spectrum displayed the characteristic triplet and quartet at δ 1.24 ppm and 4.16 ppm for the protons of ethoxy group. Two different vinylic protons are appeared as singlet signals at δ 5.22 ppm and 7.67 ppm. The ^13^C NMR spectrum exhibited distinct signals at δ 166.77, 167.46 ppm for the carbonyl groups of thiazolidine ring and ester function, respectively. Further, the IR spectra of compound **10** showed an absorption band of carbonyl group at 1739 cm^−1.^ The ^1^H NMR spectrum showed a singlet signal at 7.40 ppm is attributed to olefinic proton. Its mass spectrum showed a molecular ion peak at *m/z* = 416, corresponding to a molecular formula (C_28_H_20_N_2_O_2_).

## Biological activity

### Cytotoxicity activity

**Fig. 2:**
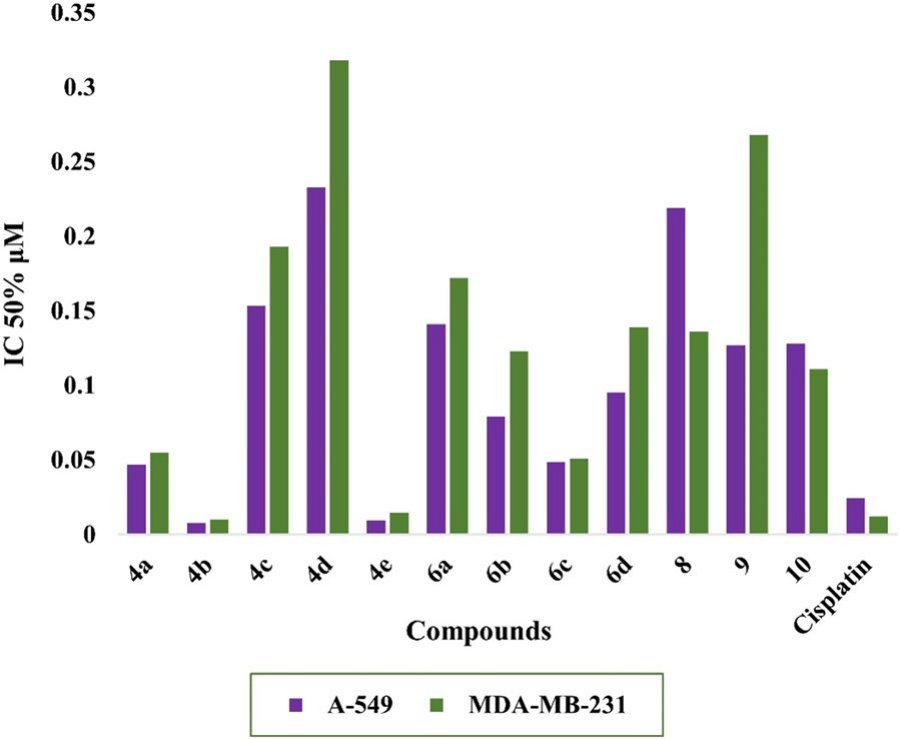
IC_50_ of the cytotoxic activity of the examined compounds against tumorigenic MDA-MB-231 and A-549 and non- tumorigenic MRC-5 cell lines

In the *vitro* cytotoxic activity of triphenylamine-based pyridine, thiazole, pyrazole and/or isoxazole derivatives was estimated against two cell lines as lung cancer (A-549) [39, 40] and mammary gland breast cancer cell line (MDA-MB-231) [41, 42] by using MTT assay according to reported method [28, 29]. Cisplatin is one of the common alkylation agents was selected to be the reference drug. From graphic plots of the dose response curve determine the 50% inhibitory concentration (IC_50_) (Fig. 2). The cytotoxicity of all the synthetized compounds was displayed in Table 1. It is found that the examined compounds exhibited different degrees of inhibitory effects on tested tumor cells. The resulting data from Table 1 showed these compounds containing pyridine moiety are better than containing thiazole moiety against two cell lines. Most pyridine derivatives showed perfect activity especially the pyridines **4b** and **4e** displayed the highest values of IC_50_ (0.00803 and 0.0095 μM) and (0.0103 and 0.0147 μM), respectively, against lung and breast cell lines. As well, the pyridine and thiazole derivatives **4a** and **6c** gave good IC_50_ values toward lung and breast cell lines (0.04685 and 0.0485 μM) and (0.055 and 0.051 μM), respectively. Furthermore, the thiazoles **6b** and **6d** recorded moderate IC_50_ values (0.0792 and 0.0954 μM) and (0.123 and 0.139 μM), respectively against tested cell line lung and breast. On other hand, the compounds **4c**, **4d**, **6a**, **8**, and **9** showed weak IC_50_ values (0.1536, 0.2328, 0.141, 0.219, and 0.127 μM) and (0.193, 318, 0.172, 0.136, and 0.268 μM), respectively against two cell line lung and breast.

**Table 1:** Cytotoxicity of synthesized compounds against **A-549**, **MDA-MB-231**, and **MRC-5** cell lines

Cpd. No	IC_50_ (μM)		CC_50_ (μM)
	**A-549 cell line**	**MDA-MB-231 cell line**	**MRC-5 cell line**
4a	0.04685	0.055	0.1372
4b	0.00803	0.0103	0.0971
4c	0.1536	0.193	0.864
4d	0.2328	0.318	1.0956
4e	0.0095	0.0147	0.0823
6a	0.141	0.172	0.9852
6b	0.0792	0.123	0.768
6c	0.0485	0.051	0.1432
6d	0.0954	0.139	0.6829
8	0.219	0.136	1.1046
9	0.127	0.268	1.295
10	0.128	0.111	0.973
Cisplatin	0.0244	0.012	0.0876

### Structure activity relationships (SAR)

On study the results, we obtained valuable data on the structure–activity relationship.

Pyridine derivatives **4b** and **4e** with substituted methyl and nitro groups at position 4 of phenyl ring exhibited a strong inhibitory effect against tested A-549 cell line with IC_50_ values (0.00803 and 0.0095 μM) and tested MDA-MB-231 cell line with IC_50_ values 0.0103 and 0.0147 μM, respectively. The pyridine analog **4a** without any substituent on the phenyl group displayed less activity (0.04685 μM) against the lung cell line. The effect of exchanging methyl and nitro groups for compounds **4b** and **4e** with methoxy and *N*,*N*-dimethyl groups for compounds **4c** and **4d** resulted in a remarkable decrease in IC_50_ values (0.1536 and 0.2328 μM) and (0.193 and 0.318 μM), respectively, toward examined cell lines.

Also, introducing a methyl group in position 4 of the phenyl ring in thiazole analog **6c** led to an increase IC_50_ 0.0485 and 0.051 μM for lung and breast cell lines, respectively. the replacing of the methyl group at position 4 for **6c** with *N*,*N*-diethyl, *N*,*N*-dimethyl, and methoxy groups for derivatives **6a**, **6b**, and **6d** led to decrease activity (IC_50_ 0.141, 0.0792, and 0.0954 μM) and (0.172, 0.123, and 0.139) against two test cells. Moreover, pyrazole recorded good potency with IC_50_ 0.127 and 0.268 μM, respectively. Finally, triphenylamine-pyridine derivatives **4b** and **4e** showed better results than drug reference against (A-549 cell line) due to containing phenyl moiety on substituted methyl and nitro group which increases activity. The nitro group (NO_2_) is reduced and forming a reactive intermediate that can damage DNA [43]. Pyridine and thiazole with phenyl ring which was substituted with a methyl group at position four revealed the highest activity against lung and breast cell lines.

### In silico physicochemical parameters (ADME) prediction

This prediction became very popular in the drug discovery and designing process, and most drugs fail because of their poor physicochemical properties [1]. The physicochemical properties of the most potent compounds **4b**, **4c**, and **6c** were represented in Table 2 by Swiss ADME online software (https://www.swiss adme.ch). The calculated results indicated these compounds **4b**, **4c**, and **6c** obeyed Lipinski’s rule of five parameters. For a compound to qualify as a ligand it should have MW ≤ 500, high lipophilicity i.e. value of Log P being ≤ 5, HB donor ≤ 5, HB acceptor ≤ 10, in addition to TPSA value was favorable with range ≤ 140 [5]. Consequently, any compound show 2 or more violations must be excluded from further study [3].

**Table 2:** Important computed physicochemical properties of the most potent compounds **4b**, **4c**, and **6c**

compounds	MW	log P	HBD	HBA	TPSA (A^2^)	n-RB	Lipinskiviolation
4b	493.56	3.83	1	3	98.84	5	0
4c	509.56	3.30	1	4	108.07	6	1
6c	604.72	3.30	1	3	106.37	8	1
Lipinski/Veber’s Rules	≤ 500	≤ 5	≤ 5	≤ 10	≤ 140	≤ 10	≤ 1

*MW* Molecular weight, *LogP* Logarithm of partition coefficient between n-octanol and water, *HBD* Hydrogen-bonded donor, *HBA* Hydrogen-bonded acceptor, *TPSA* Topological polar surface area, *n-RB* Number of rotatable bonds

### Docking analysis

The docking scores, bond distances, and interactions of the target ligands with specific receptors were presented in Table 3. It was found from tabulated results that, the synthesized compounds under study showed perfect fitting inside the active sites of the proteins surface. The protein (2ITO) exhibited score of binding free energy in the range from − 5.0889 to − 8.1868 kcal/mol and the binding scores of compounds decrease in the order **6c** > **6a** > **6b** > **6d** > **4a** > **4d** > **4b** > **4c** > **8** > **4e** > **9** > **10**. But, the protein (2A4L) showed the range from − 6.44278 to − 9.3507 kcal/mol and scores of derivatives decrease in the order **6a** > **6c** > **6b** > **4c** > **4d** > **4b** > **6d** > **4e** > **4a** > **9** > **10** > **8**.

**Table 3:** Predictive docking scores and particular interactions of the ligands and the target proteins

(PDB ID: 2ITO)							(PDB ID: 2A4L)					
Cpd. No	S (Binding energy score) kcal/mol	RMSD	Distance (Å)	Binding interactions			S (Binding energy score) kcal/mol	RMSD	Distance (Å)	Binding interactions		
				Ligand	Receptor	Interaction type				Ligand	Receptor	Interaction type
4a	− 6.9765	1.4908	9.75	N-nitrile group	Gly 863	H-acceptor	− 7.9080	0.5212	2.82 3.02 3.96	N-amino group N-nitrile group Pyridone-ring	Thr 14 Lys 33 Gly 13	H-donor H-acceptor π-H
4b	− 6.8536	1.1121	3.17	N-nitrile group	Arg 836	H-acceptor	− 8.3395	1.2150	2.82 3.01 3.98	N-amino group N- nitrile group Pyridone-ring	Thr 14 Lys 33 Gly 13	H-donor H-acceptor π-H
4c	− 6.6658	1.1121	3.33 3.28 4.58 4.42 4.16	O-carbon-yl group N-nitrile group Triphe-nylamine- ring Pyrido-ne-ring Pyrido-ne-ring	Gly 863 Arg 836 Arg 889 Ile 890 Tyr 891	H-acceptor H‐acceptor π-H π-H π-H	− 8.5695	1.2341	2.83 3.56 3.01 3.84	N-amino group N-amino group N- nitrile group Pyridone-ring	Thr 14 Asp 145 Lys 33 Gly 13	H-onor H-donor H-acceptor π-H
4d	− 6.9206	1.2560	3.63 3.06	N- nitrile group N- nitrile group	Leu 862 Gly 863	H-acceptor H-acceptor	− 8.5127	1.3549	2.86 3.62 3.00 3.90	N-amino group N-amino group N- nitrile group Pyridone-ring	Thr 14 Asp 145 Lys 33 Gly 13	H-donor H-donor H-acceptor π-H
4e	− 6.4935	1.3124	3.10	N- nitrile group	Arg 836	H-acceptor	− 8.0316	1.4260	3.59 3.53 3.41 3.48 4.50 4.17	N- nitrile group N- nitrile group N-nitrile group O-nitro group benzene-ring Phenyl-ring	Lys 129 Asn 132 Gly 11 Leu 83 Val 18 Glu 162	H-acceptor H-acceptor H-acceptor H-acceptor π-H π-H
6a	− 7.8775	1.1983	3.60	N-nitrile group	Arg 889	H-acceptor	− 9.3507	1.3088	2.92 4.16	O-carbon-yl groyp Triphenyla-mine-unit	Lys 89 Gln 131	H-acceptor π-H
6b	− 7.3815	1.6009	3.31 4.54 4.09	N- nitrile group Triphe-nylamine- ring Triphenyl-amine- ring	Lys 860 Lys 757 Lys 757	H-acceptor π-H π-cation	− 8.8483	1.1133	3.18 2.84 3.90 4.14	S-thiazole O-carbon-yl of thiazole Benzene ring Triphenyla-mine-unit	Asp 86 Lys 89 Gly 13 Lys 89	H-donor H-acceptor π-H π-cation
6c	− 8.1868	1.4149	2.96 2.97 4.04	O-carbonyl of thiazole O- carbonyl of thiazole Triphe-nylamine- ring	Arg 831 Arg 831 Ala 698	H-acceptor H-acceptor π-H	− 8.9306	1.4433	2.89 4.15	O-amidic group Triphenyla-mine-unit	Lys 89 Gln 131	H-acceptor π-H
6d	− 7.1931	1.2501	3.46 3.62	N-nitrile group Triphe-nylamine- ring	Lys 860 Arg 836	H-acceptor π-cation	− 8.1565	1.1511	3.82 3.32 3.06 3.75	S-thiazole O- carbon-yl of thiazol-e O-methoxy group Triphenyla-mine-unit	Asp 86 Lys 89 Lys 129 Lys 89	H-donor H-acceptor H-acceptor π-cation
8	− 6.5819	1.1682	3.06	O- carbonyl of thiazole	Glu 866	H-acceptor	− 7.3087	0.9759	3.64 3.82 4.46 4.28	S-thiazole S-thiazole Triphenyla-mine-unit Triphenyla-mine-unit	Asp 86 Gln 131 His 84 Lys 89	H-donor H-donor π-H π-cation
9	− 6.4760	1.0055	4.71 4.61 4.20 4.22 4.39	Triphe-nylamine- ring Triphe-nylamine- ring Benzen-e-ring Benzen-e-ring Pyrazol-one-ring	Glu 866 Arg 889 Ile 890 Tyr 891 Tyr 891	π-H π-H π-H π-H π-H	− 7.8219	1.3173	4.37 4.72	Triphenyla-mine-unit Benzene-ring	Val 18 Thr 165	π-H π-H
10	− 5.0889	1.3670	3.24 2.92	C- Benzene-ring O-carbonyl of isoxazo-le	Glu 866 Lys 754	H‐donor H-acceptor	− 7.3885	1.4170	3.52 3.96	N-isoxazole ring Triphenyla-mine-unit	Leu 83 Val 18	H-acceptor π-H
cisplatin	− 3.0191	0.4224	2.96 3.16 2.91 2.89 2.96 3.16 2.89	N-amino group N-amino group N-amino group N- amino group N- amino group N- amino group N- amino group	Glu 697 Glu 697 Ala 698 Glu 697 Glu 697 Glu 697 Glu 697	H‐donor H‐donor H‐donor H‐donor Ionic Ionic Ionic	− 2.7187	1.4175	3.33 3.33	N-amino group N-amino group	ASP 145 ASP 145	H-donor Ionic

### The docking scores, bond distances, and interactions of the target ligands with protein (2ITO)

Pyridine derivatives **4a**, **4b**, and **4e** showed binding scores (S =  − 6.9765, − 6.8536, and − 6.4935 kcal/mol), respectively over a significant H-acceptor bond between N-atom of nitrile group with amino acid Gly 863 and Arg 836 through an intermolecular distance 9.75, 3.17, and 3.10 Å, respectively (Additional file 1: Figs. S65, S66 and S67). While pyridine derivative **4c** containing methoxy group presented a binding score (S = − 6.6658 kcal/mol). The interested active sites of the receptor showed five intermolecular attractions, two H-acceptor bonds between O-atom of pyridone ring with Gly 863 and N-atom of nitrile group with Arg 836 through intermolecular distances (3.33 and 3.28 Å), respectively. Three π-H bonds, one between triphenylamine ring with Arg 889 and the others between pyridone ring with Ile 890 and Tyr 891 (4.58, 4.42, and 4.16 Å), respectively (Additional file 1: Fig. S68).

**Fig. 3:**
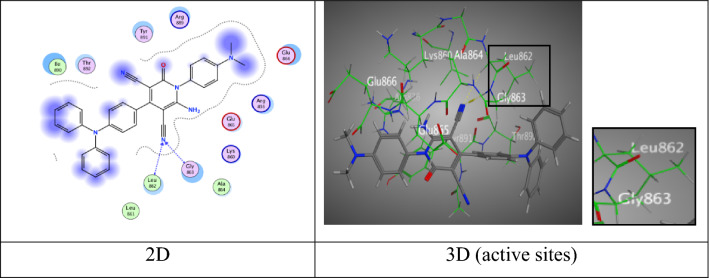
Interactions of **4d** with the residues of (PDB ID: 2ITO) (blue:  H-acceptor bond)

Where, pyridine derivative **4d** displayed two H-acceptor bonds between N-atom of (C≡N) group with Leu 862 and Gly 863 (3.63 and 3.06 Å) through energy score equal − 6.9206 kcal/mol (Fig. 3).

**Fig. 4:**
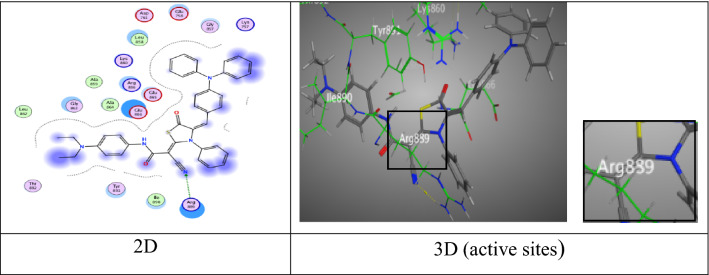
Interactions of **6a** with the residues of (PDB ID: 2ITO) (green:  H-acceptor bond).

Moreover, thiazolidine-5-one derivative **6a** exhibited a good binding free energy equal − 7.8775 kcal/mol came from one H-acceptor bond among N-atom of nitrile group (C≡N) with Arg 889 (3.60 Å) (Fig. 4).

**Fig. 5:**
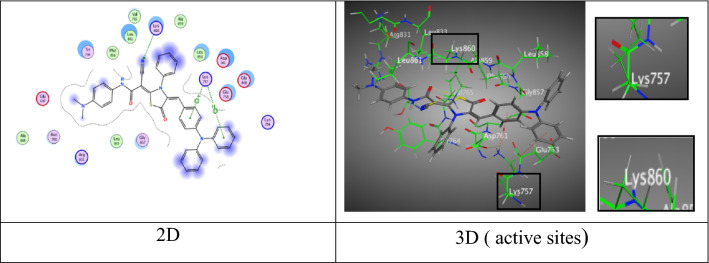
Interactions of **6b** with the residues of (PDB ID: 2ITO) (green:  H-acceptor bond, green .: π-H, green : π-cation)

Further, thiazolidine-5-one derivative **6b** exhibited three attractions. One hydrogen bond between N-atom of nitrile group with Lys 860 (3.31 Å), one π-H bond between triphenylamine ring with Lys 757 (4.54 Å), and one π-cation bond among another ring of triphenylamine with Lys 757 (4.09 Å) through respectable docking score equal (S = − 7.3815 kcal/mol) (Fig. 5).

**Fig. 6:**
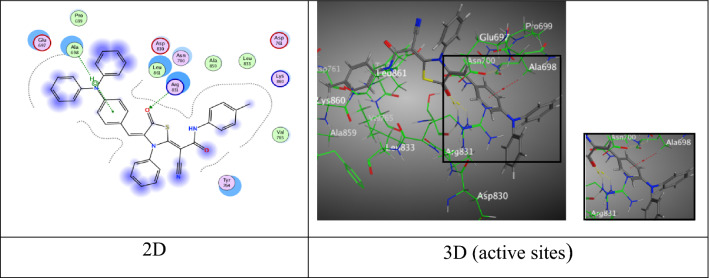
Interactions of **6c** with the residues of (PDB ID: 2ITO) (green:  H-acceptor bond, green : π-H).

Moreover, thiazolidine-5-one derivative **6c** gave an eminent binding free energy equal − 8.1868 kcal/mol, and showed two H-acceptor bonds amidst O-atom of carbonyl group of thiazolidine-5-one moiety with two different active sides of Arg 831 (2.96 and 2.97 Å), besides forming one π-H interaction with triphenylamine ring and Ala 698 (4.04 Å) (Fig. 6).

**Fig. 7:**
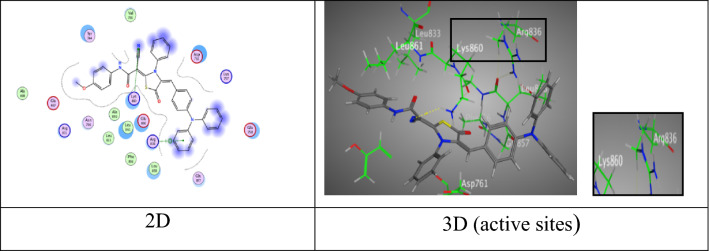
Interactions of **6d** with the residues of (PDB ID: 2ITO) (green:  H- acceptor bond green : π-cation).

Through the docking process, thiazolidine-5-one derivative that containing methoxy group **6d** revealed one H-acceptor bond between N-atom of (C≡N) group with Lys 860 at an intermolecular distance (3.46 Å), π-cation interaction between triphenylamine ring with Arg 836 (3.62 Å) by proper binding score (S = − 7.1931 kcal/mol) (Fig. 7).

Furthermore, thiazolidine-4-one derivative **8** showed binding energy (S =  − 6.5819 kcal/mol) (Additional file 1: Fig. S69) and formed one H-acceptor among O-atom of carbonyl group of thiazolidine-4-one moiety with amino acid Glu 866 (3.06 Å). On the other hand, pyrazolone derivative **9** with energy score (S = − 6.4760 kcal/mol) formed five π-H interactions, two amongst triphenylamine ring with Glu 866 and Arg 889, two between phenyl ring with ILE 890 and Tyr 891, and the last between pyrazolone ring with Tyr 891 over intermolecular distances (4.71, 4.61, 4.20, 4.22, and 4.39 Å), respectively (Additional file 1: Fig. S70). Finally, isoxazolone derivative **10** displayed two attractions, the first is H‐donor bond between C-atom of phenyl ring with Glu 866 (3.24 Å) and the second is H-acceptor bond among carbonyl group with Lys 754 (2.92 Å) through energy score equal ‐5.0889 kcal/mol (Additional file 1: Fig. S71).

### The docking scores, bond distances, and interactions of the target ligands with protein (2A4L)

Pyridine derivatives **4a** and **4b** displayed three intermolecular attractions (Additional file 1: Fig. S72 and S73). One hydrogen-donor bond between N-atom of the amino group with Thr 14 (2.82 Å), one hydrogen-acceptor bond amidst N-atom of nitrile group with Lys 33 (3.02 and 3.01 Å), respectively, and the last interaction was a π-H interaction between pyridone ring with Gly 13 (3.96 and 3.98 Å), respectively. While, pyridine derivatives **4c** and **4d** showed proper binding scores (S =  − 8.5695 and − 8.5127 kcal/mol) over significant attractions, three hydrogen bonds with Thr 14, Asp 145, and Lys 33 (3.01 and 3.00 Å), and one π-H bond with Gly 13 (3.84, 3.90 Å) (Additional file 1: Fig. S74 and Fig. S75). Otherwise, the molecular docking of 2-pyridone substituted with nitro group **4e** with active site of protein; offered six interactions. Four hydrogen bonds between two N-atoms of the two (C≡N) groups, and O-atom of nitro group with the active site of Lys 129, Asn 132, Gly 11, and N-atom of Leu 83, respectively. Two π-H interactions, one between phenyl ring containing nitro group with Val 18, and another among phenyl ring with Glu 162 through binding energy score (S =  − 8.0316 kcal/mol) (Additional file 1: Fig. S76).

**Fig. 8:**
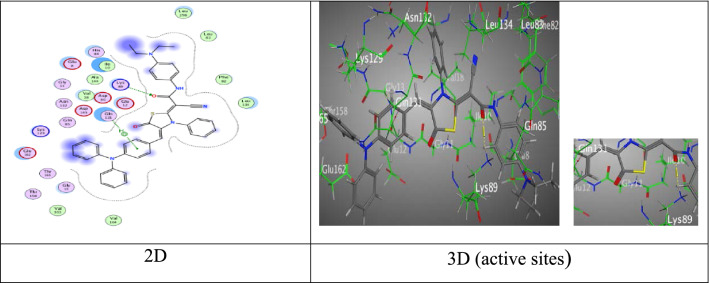
Interactions of **6a** with the residues of (PDB ID: 2A4L) (green: H-acceptor bond, green …..: π-H).

Meanwhile, thiazolidine-5-one derivative **6a** gave eminent binding energy (− 9.3507 kcal/mol) came from two intermolecular attractions, the first hydrogen acceptor-bond between O-atom of amide group with Lys 89 (2.92 Å), the second π-H interaction appeared through a distance 4.16 Å between phenyl ring of triphenylamine unit with Gln 131 (Fig. 8).

**Fig. 9:**
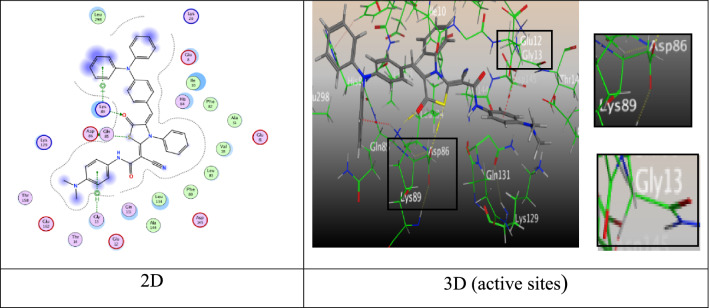
Interactions of **6b** with the residues of (PDB ID: 2A4L) (green: H-acceptor bond, green : π-H, green : π-cation).

Further, tiazolidine-5-one derivative **6b** exhibited four attractions. Two hydrogen bonds between sulfur-atom and oxygen-atom of carbonyl group of thiazolidine ring with Asp 86 and Lys 89 at distance (3.18 Å and 2.84 Å), respectively, one π-H bond between *N,N*-dimethylamine ring with Gly 13 at distance (3.90 Å) (Fig. 9), in addition to π-cation bond among triphenylamine unit with Lys 89 at distance (4.14 Å) through respectable docking score equal (S =  − 8.8483 kcal/mol).

**Fig. 10:**
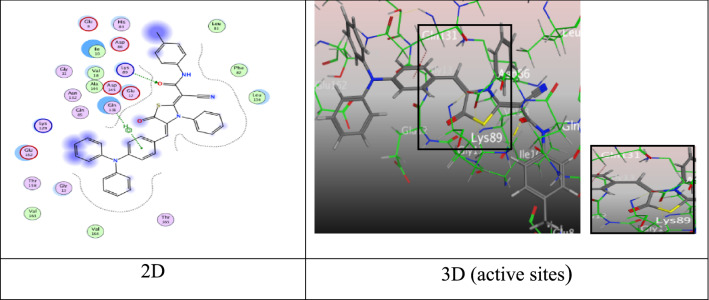
Interactions of **6c** with the residues of (PDB ID: 2A4L). (green: H-acceptor bond, green : π-H).

Moreover, tiazolidine-5-one derivative **6c** good binding free energy equal − 8.9306 kcal/mol, and showed H-acceptor bond amidst oxygen-atom of carbonyl group of amide moiety with Lys 89 (2.89 Å), besides forming one π-H interaction with triphenylamine ring and Gln 131 (4.15 Å) (Fig. 10).

Also, the compounds **6d**, **8**, **9**, and **10** displayed proper binding scores (S =  − 8.1565, − 7.3087, − 7.8219, and − 7.3885 kcal/mol), respectively, and formed hydrogen bonds and π-H interactions with different residues such as Asp 86, Lys 89, Lys 129, Lys 89, Gln 131, His 84, Val 18, Thr165, Leu 83, and Val 18 (Additional file 1: Fig. S77, S78, S79, and S80).

Standard compound for the docking is cisplatin. It was docked with the two proteins 2ITO and 2A4L and displayed different interactions. The interactions of cisplatin with protein (2ITO) showed as following: four H-donor bonds and three ionic bonds. Three H-bonds between N-atom of amino group with Glu 697, Glu 697, and Ala 698 through intermolecular distances (2.96, 3.16, 2.91 Å), respectively, the last H-donor between N-atom of another amino group with Glu 697 at 2.89 Å. The two ionic bonds between N-atom with Glu 697 at (2.96, 3.16 Å), and another ionic bond between N-atom with Glu 697 at 2.89 Å. (Additional file 1: Fig. S81). Moreover, cisplatin with protein (2A4L) revealed H-donor and ionic bond between N-atom with Asp 145 at the same distance (3.33 Å) (Additional file 1: Fig. S82).

The active sites in docking studies were determined from residues which had high alignment. According to protein (2ITO): The pyridine derivatives **4a**, **4c**, and **4d** formed H-acceptor bond with the amino acid Gly 863, in addition to these compounds **4b**, **4c**, **4e**, and **6d** showed H-acceptor bond with the same residue Arg 836, further the derivatives **4c**, **6a**, and **9** displayed with Arg 889 and Ile 890 pi and hydrogen interactions, finally compounds **8**, **9**, and **10** displayed H-acceptor, pi, H-donor bonds with Glu 866, respectively. But, the most active sites of the protein (2A4L) are Thr 14, Lys 33, and Gly 13 residues which formed hydrogen bonds and pi interactions with compounds **4a**, **4b**, **4c**, and **4d**. Moreover, the thiazoles **6a-6d** offered hydrogen bonds with Lys 89.

### Structure activity relationships of compounds in comparison with docking and biological activity

The pyridine analog with substituent methyl group **4b** demonstrated eminent activity with IC_50_ 0.00803 and 0.0103 μM toward lung and breast cell lines, respectively, and showed good binding scores (S =  − 6.8536 and − 8.3395 kcal/mol) with proteins 2ITO and 2A4L through H-donor and acceptor hydrogen bonds and π-H interaction. On the other hand, the pyridine derivative **4e** recorded high half-maximal inhibitory concentrations at 0.0095 and 0.0147 μM, respectively, and proper energy scores with selected proteins equal − 6.4935 and − 8.0316 kcal/mol came from H-acceptor bonds and π-H interactions. Moreover, the thiazole derivative **6a** revealed IC_50_ 0.141 μM for lung cell line, when docked with protein 2A4L gave eminent binding energy (S =  − 9.3507 kcal/mol) that achieved through H-acceptor bond between O-atom of the carbonyl group with Lys 89 and π-H interaction between phenyl ring with Gln 131. Over and above, compound **6c** showed a proper IC_50_ value 0.0485 μM for the lung cell line and formed two H-acceptor and π-H bonds with Arg 831 and Ala 698 through a good energy score equal − 8.1868 kcal/mol. We found from previous data the compounds which showed higher activity against tested cell lines also recorded a perfect energy score.

## Conclusion

In this summary, we synthesized four new classes of 2-pyridones **4a-e**, thiazolidin-5-ones **6a-d**, isoxazolone, and pyrazolone analogous. The starting compound, 4-(diphenylamino)benzaldehyde (**1**) was obtained through the application of the Vilsmeier-Haack reaction. These novel classes were synthesized in good yield with simple conditions of Knoevenagel condensation reactions. The activity of these compounds was investigated against two cancer cell lines, lung (A-549) and breast cell lines (MDA-MB-231). It was found the results of lung cancer cell line more effective than breast cancer cell line. Among all derivatives, 2-pyridone derivatives exhibited remarkably half-maximal inhibitory concentrations (IC_50_). Pyridine derivative containing methyl substituent on the phenyl ring **4b** demonstrated distinct activity against both the tested lung and breast cell lines with IC_50_ values 0.00803 μM and 0.0103 μM, respectively. Molecular docking has been used to predict the binding of molecules with active sides of the proteins (2ITO and 2A4L) and confirm the biological efficiency of synthesized compounds. The docking results showed the synthesized compounds displayed good binding scores with the residues of the proteins. Meanwhile, thiazolidin-5-one derivatives recorded remarkable scores specially, thiazole analogue with substituted methyl group **6c** offered an eminent docking score (S =  − 8.1868 kcal/mol) with the protein 2ITO and the thiazole with *N*,*N*-di-ethylamine substituent **6a** gave distinguished energy score (− 9.3507 kcal/mol) with 2A4L.

## Supplementary Information

**Additional file 1: Figures S1–S82.**Additional figures S1–S2.

## Data Availability

The datasets used and/or analyzed during the current study available from the corresponding author on reasonable request.

## Abbreviations

MDA-MB-231: Mammary carcinomas
A-549: Lung cancer
DMSO-*d*6: Dimethyl Sulfoxide-deuterated
CDCl_3_: Chloroform deuterated
HEPES: (4-(2-Hydroxyethyl)-1-piperazineethanesulfonic acid)
DMEM: Dulbecco’s modified Eagle’s medium
MTT: 3-(4,5-Dimethylthiazol-2-yl)-2,5-diphenyltetrazolium bromide
ODt: Optical density of cells treated
ODc: Optical density of control cells
IC_50_: The concentration of drug which exhibited 50% cell viability for cell
