# Supplementary material for: Synthesis, biological evaluation and molecular docking of new triphenylamine-linked pyridine, thiazole and pyrazole analogues as anticancer agents
Source: BMC Chem. 2022 Nov 7;16(1):88. doi: 10.1186/s13065-022-00879-x (PMC9641905; doi:10.1186/s13065-022-00879-x)
Supplement: Supplementary file 1 — Additional file 1: Figures S1–S82.Additional figures S1–S2. [file 13065_2022_879_MOESM1_ESM.docx]

**Synthesis, biological evaluation and molecular docking of new triphenylamine-linked pyridine, thiazole and pyrazole analogues as anticancer agents**

Mohamed R. Elmorsy^1*^, Samar E. Mahmoud^1^, Ahmed A. Fadda^1^, Ehab Abdel-Latif^1^, Miral A. Abdelmoaz^2^

^1^Department of Chemistry, Faculty of Science, Mansoura University, 35516 Mansoura, Egypt.

^2^Department of Pharmaceutical Chemistry, Faculty of Pharmacy, Sinai University, Kantra, Egypt.

^*^Correspondence: m.r.elmorsy@gmail.com

**1. Spectral analysis:**

**Figure (S1): IR spectrum of compound 3a**

**
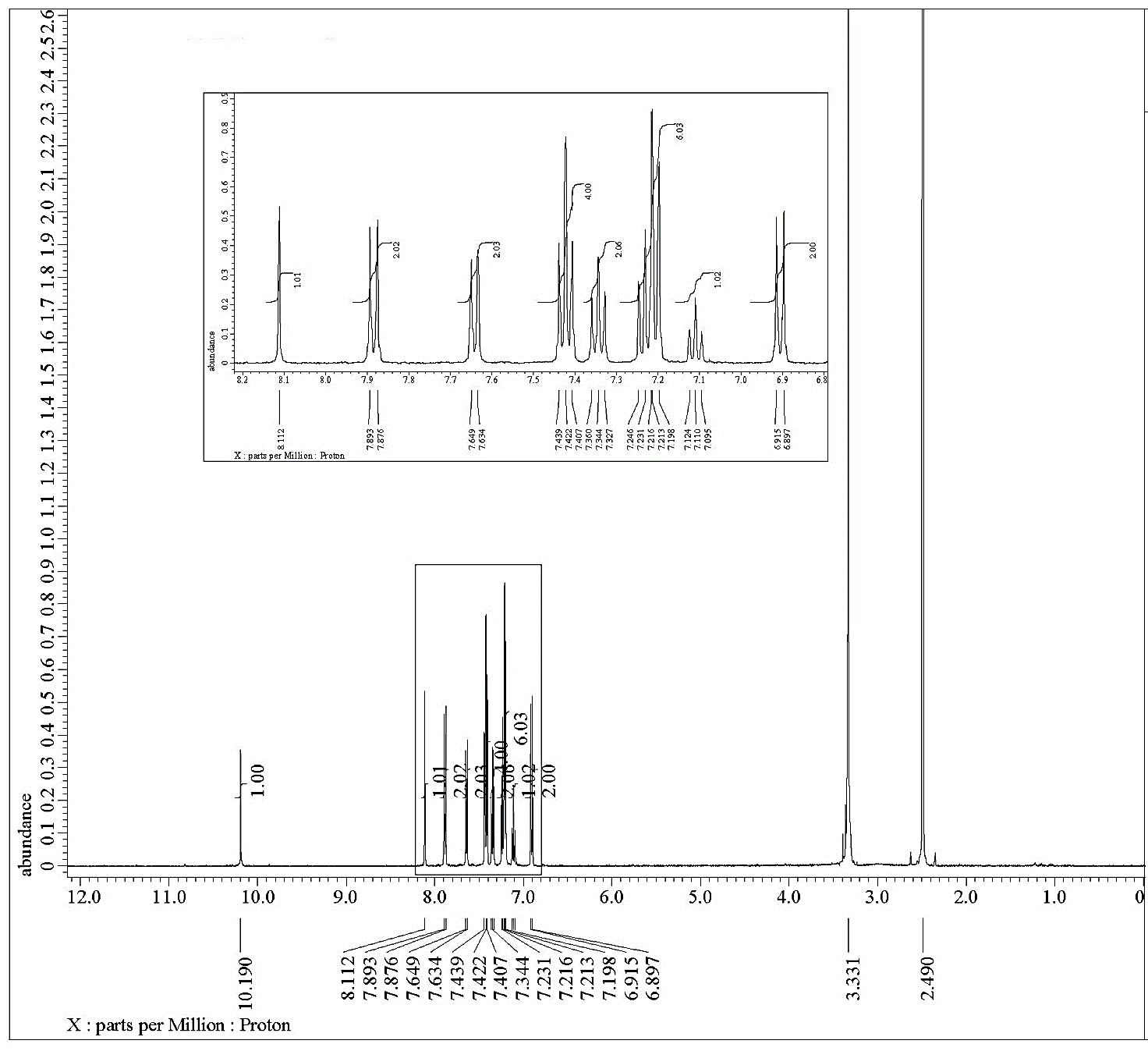
**

**Figure (S2): ^1^H NMR spectrum of compound 3a**

**
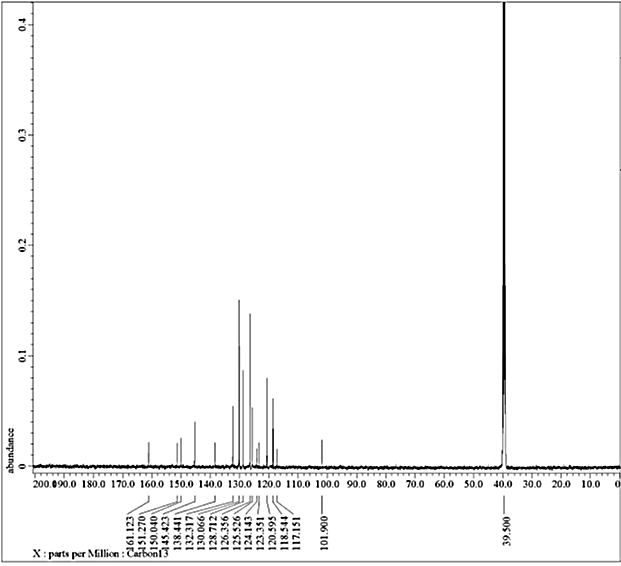
**

**Figure (S3): ^13^C NMR spectrum of compound 3a**

**Figure (S4): Mass spectrum of compound 3a**

**
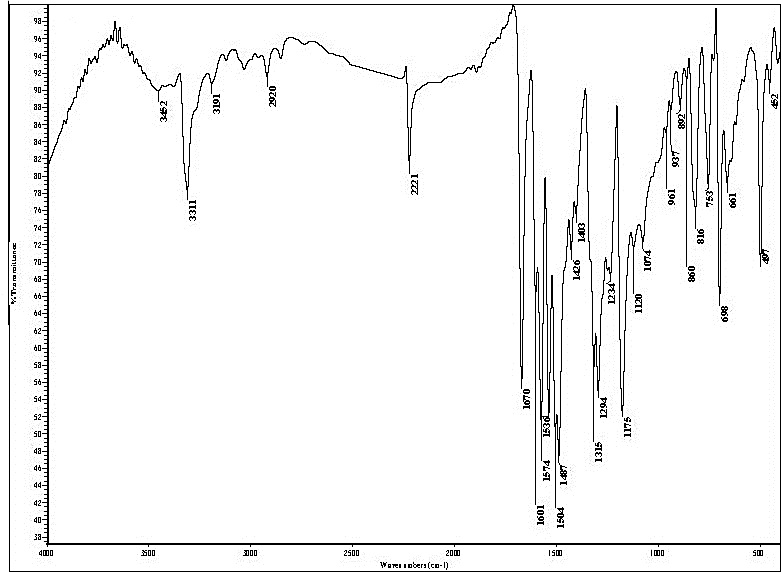
**

**Figure (S5): IR spectrum of compound 3b**

**
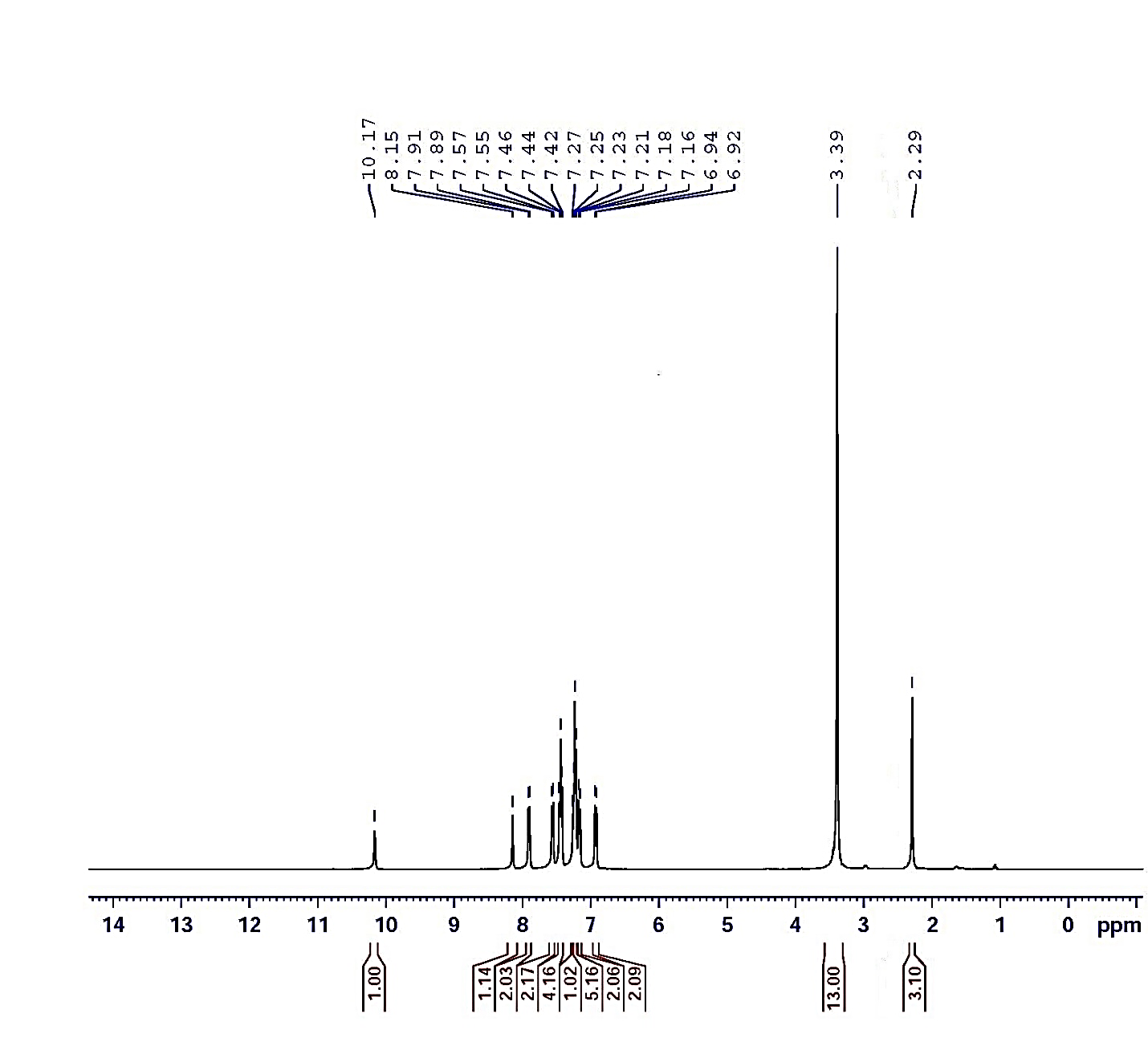
**

**Figure (S6): ^1^H NMR spectrum of compound 3b**

**
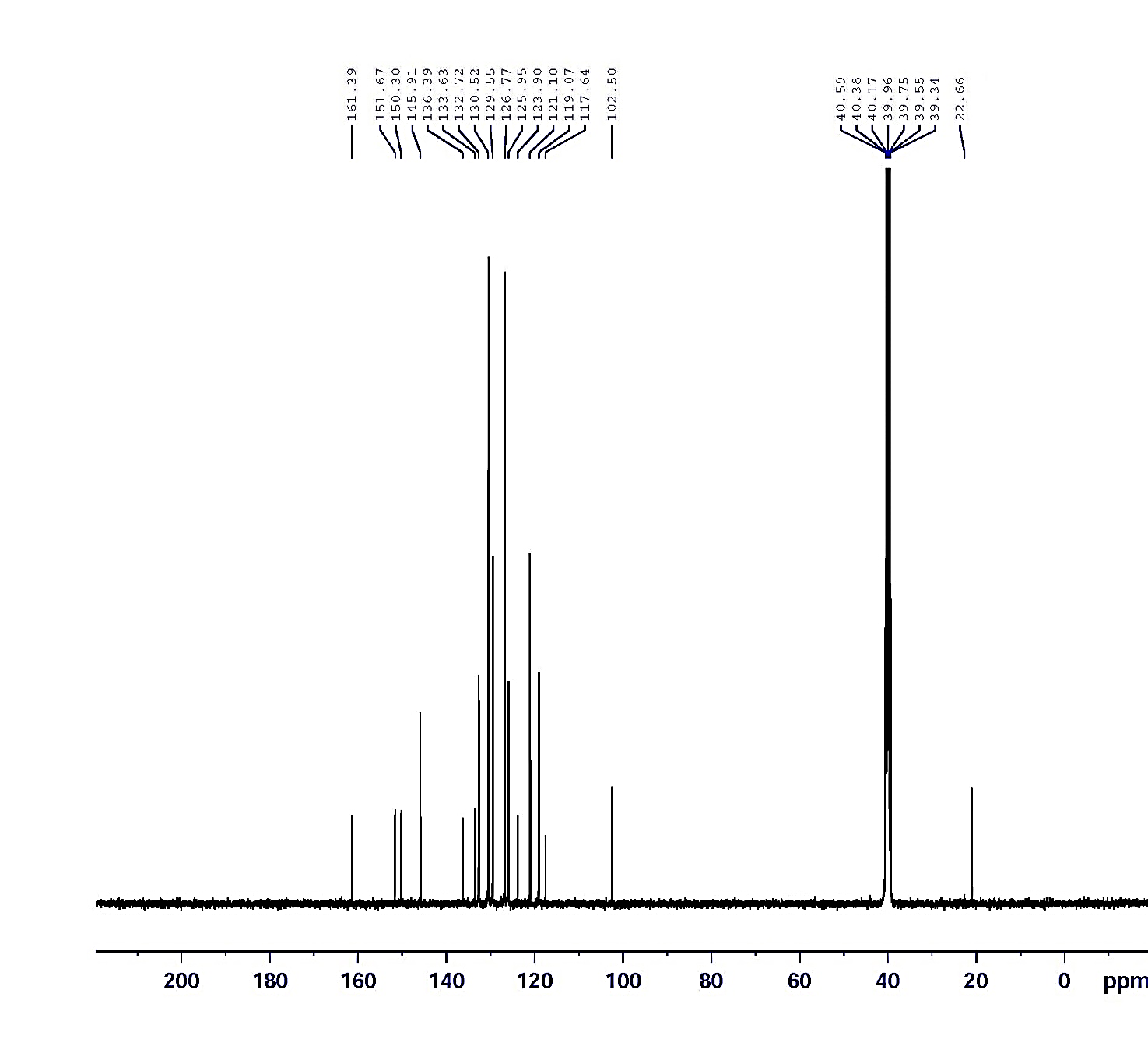
**

**Figure (S7): ^13^C NMR spectrum of compound 3b**

**Figure (S8): Mass spectrum of compound 3b**

**
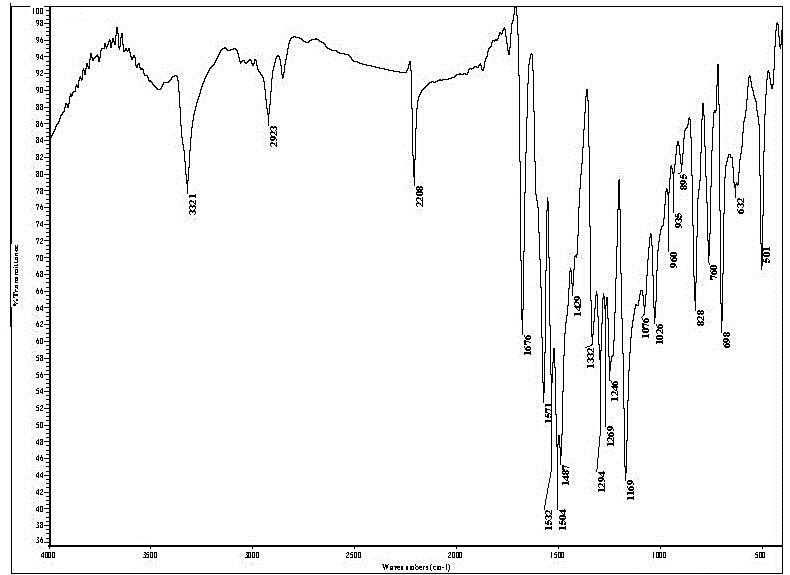
**

**Figure (S9): IR spectrum of compound 3c**

**
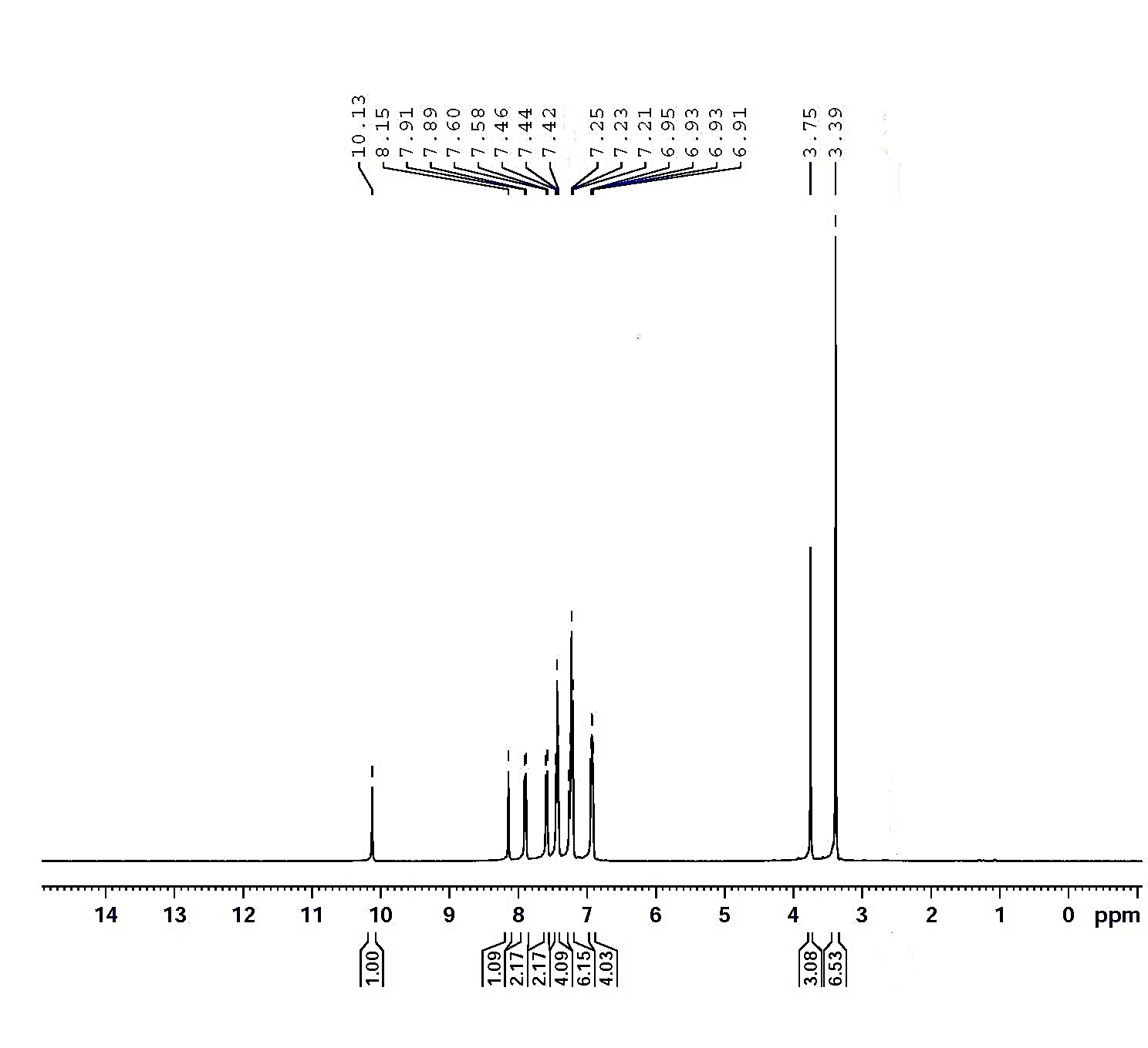
**

**Figure (S10): ^1^H NMR spectrum of compound 3c**

**
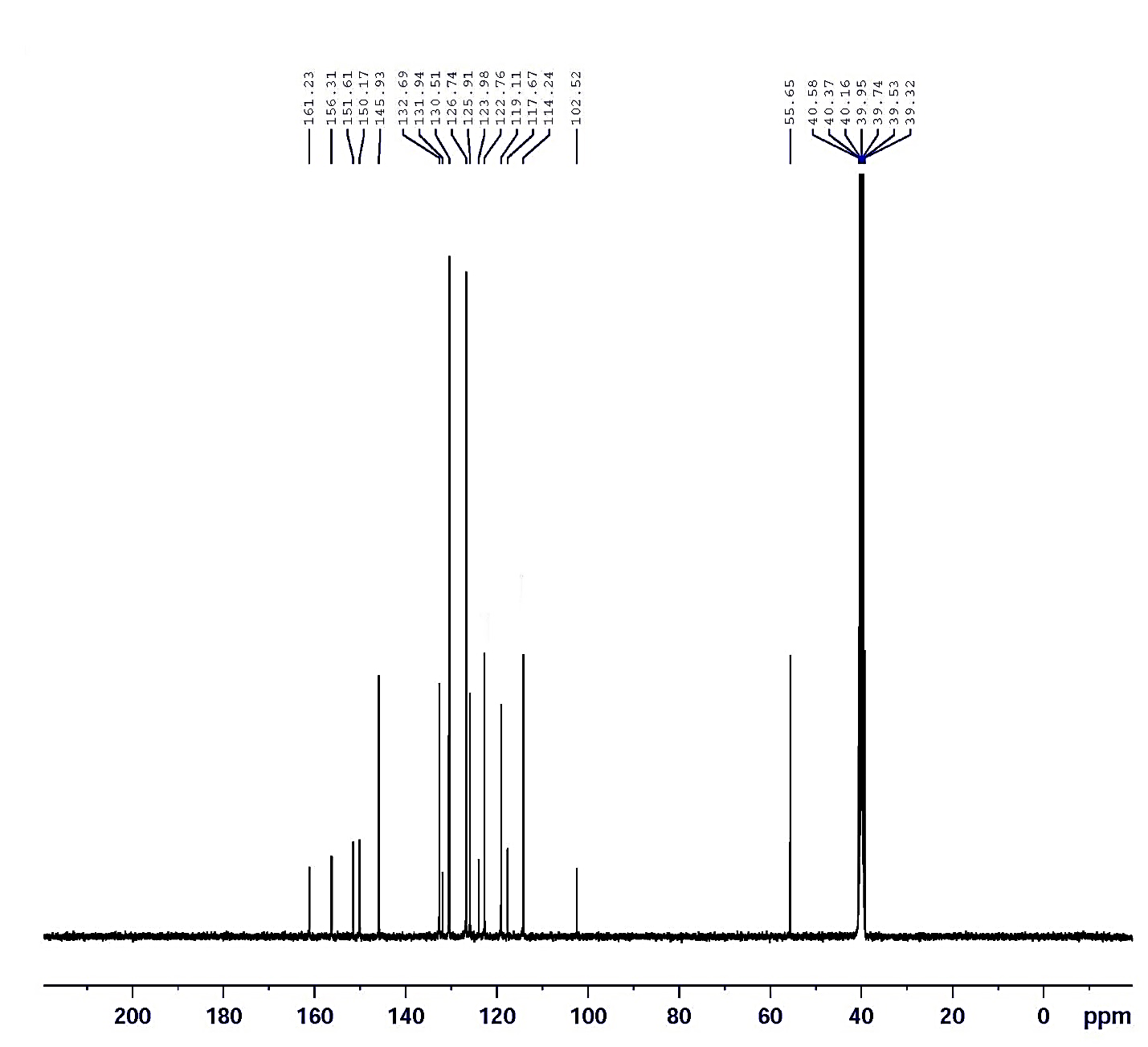
**

**Figure (S11): ^13^C NMR spectrum of compound 3c**

**Figure (S12): Mass spectrum of compound 3c**

**
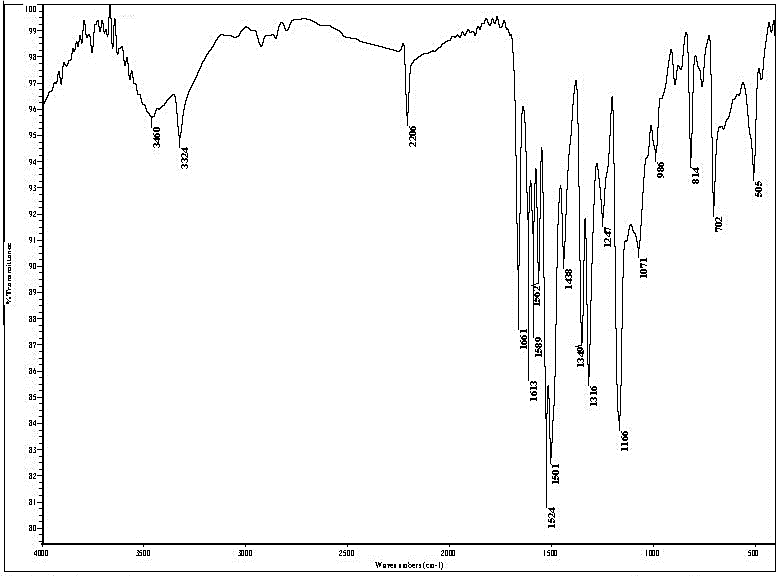
**

**Figure (S13): IR spectrum of compound 3d**

**
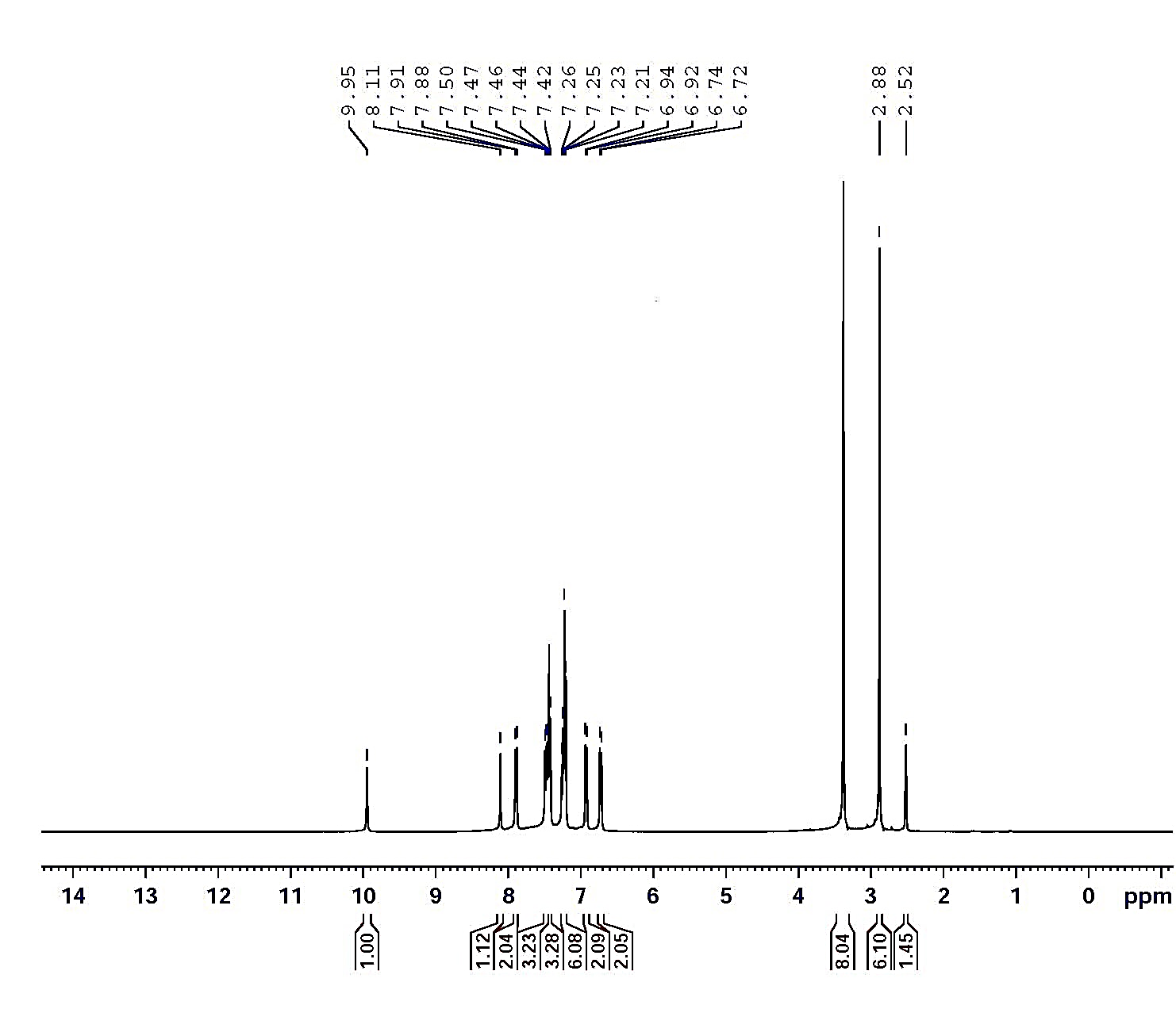
**

**Figure (S14): ^1^H NMR spectrum of compound 3d**

**
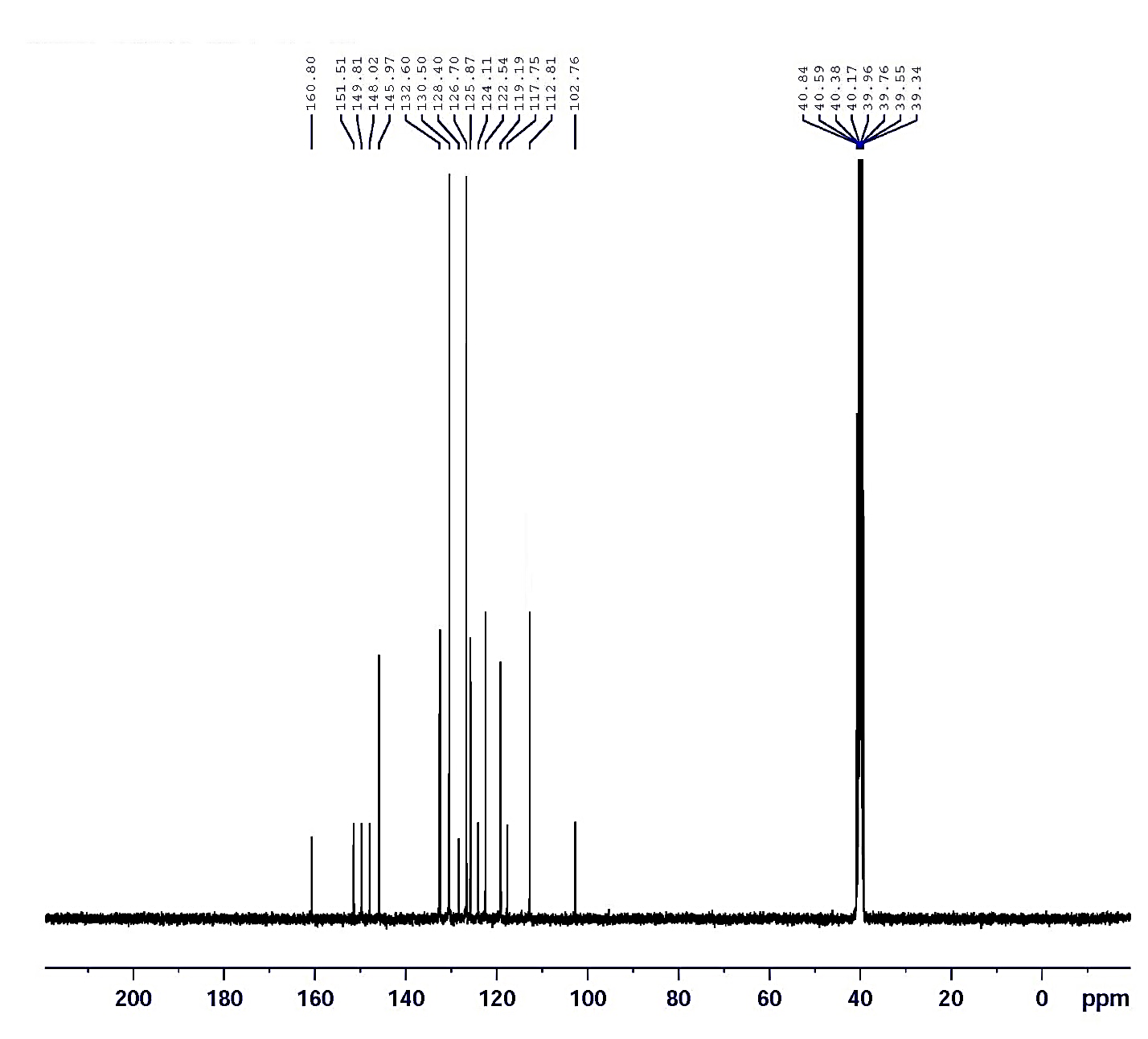
**

**Figure (S15): ^13^C NMR spectrum of compound 3d**

**Figure (S16): Mass spectrum of compound 3d**

**Figure (S17): IR spectrum of compound 3e**

**Figure (S18): ^1^H NMR spectrum of compound 3e**

**Figure (S19): ^13^C NMR spectrum of compound 3e**

**Figure (S20): Mass spectrum of compound 3e**

**
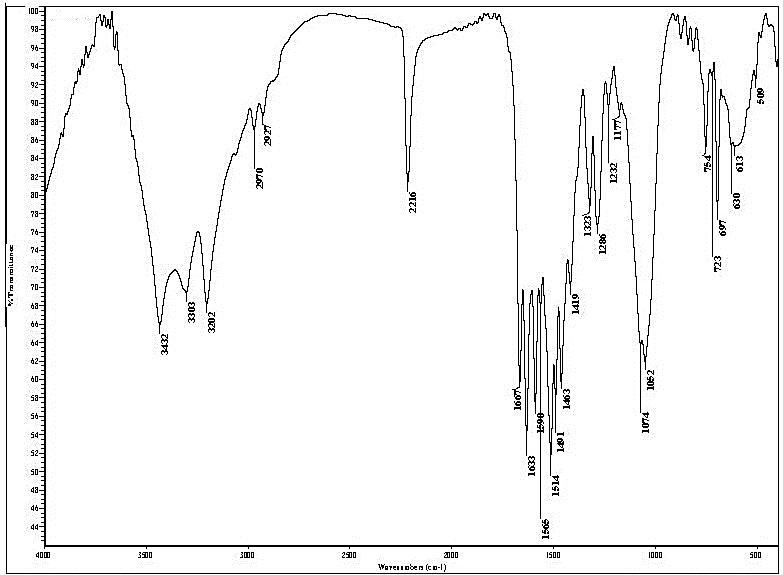
**

**Figure (S21): IR spectrum of compound 4a**

**
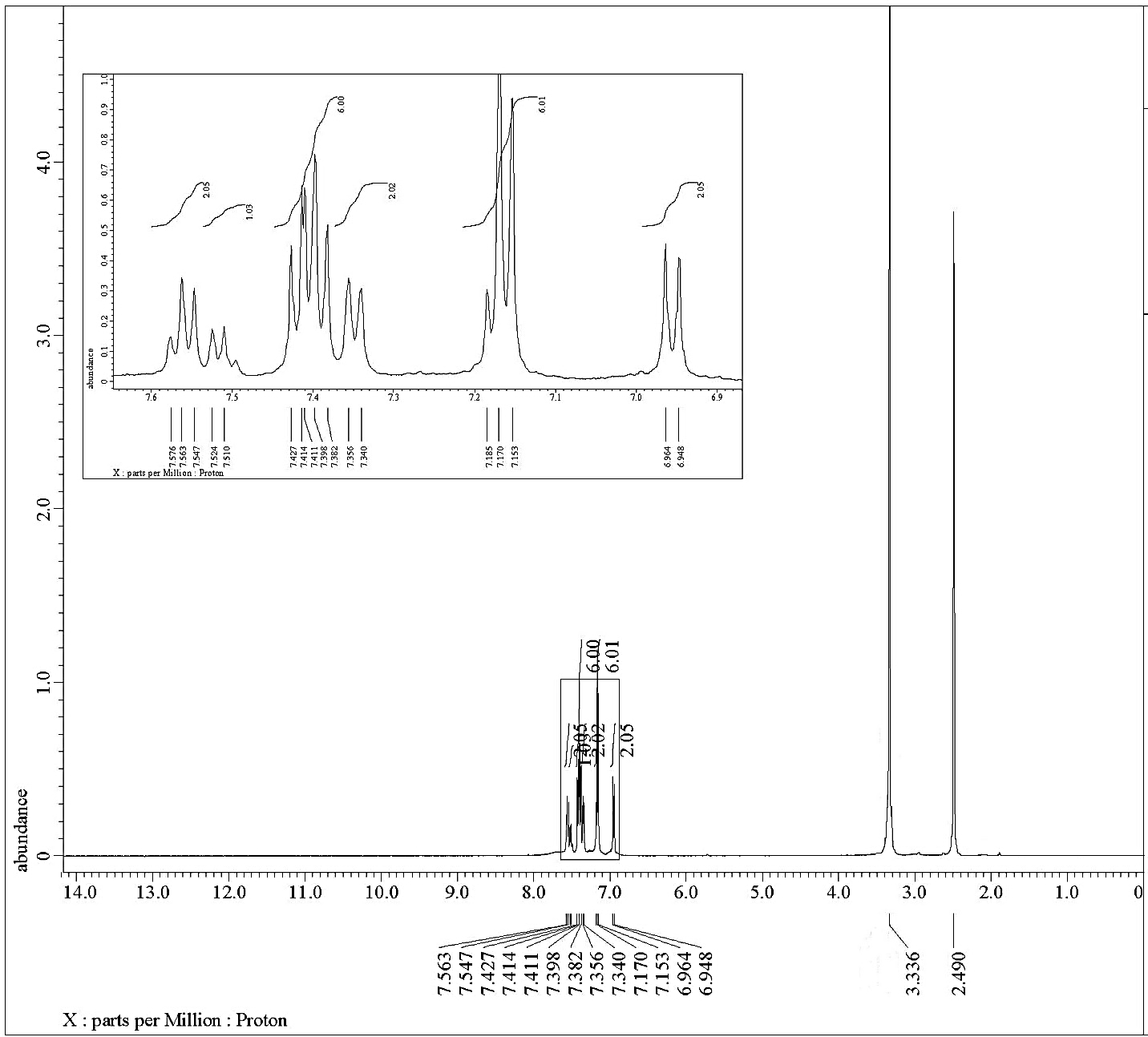
**

**Figure (S22): ^1^H NMR spectrum of compound 4a**

**
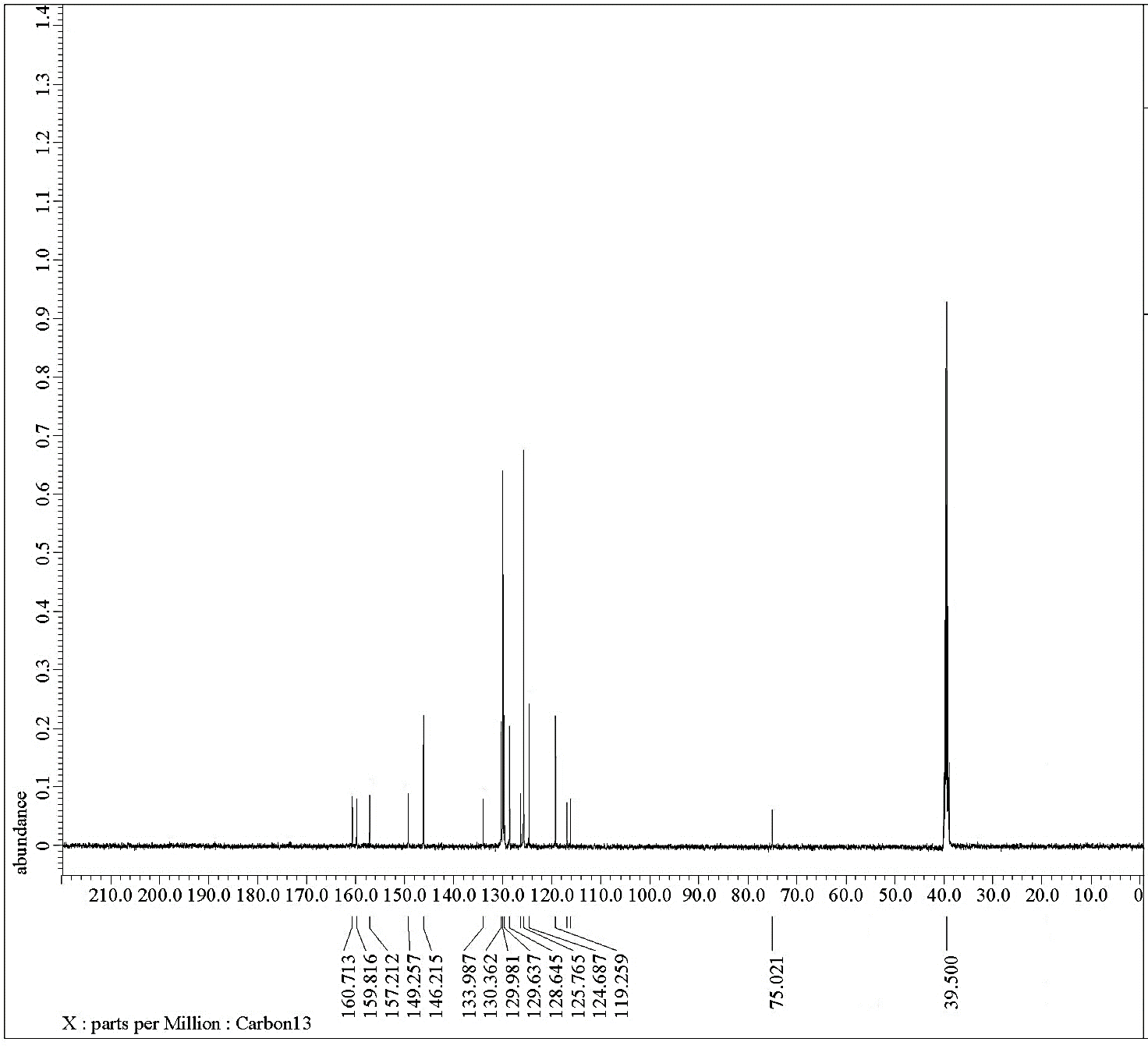
**

**Figure (S23): ^13^C NMR spectrum of compound 4a**

**Figure (S24): Mass spectrum of compound 4a**

**
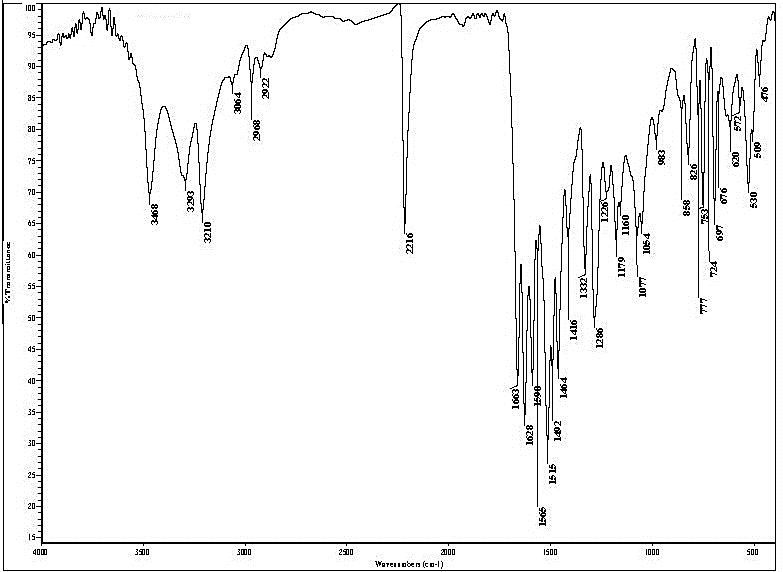
**

**Figure (S25): IR spectrum of compound 4b**

**
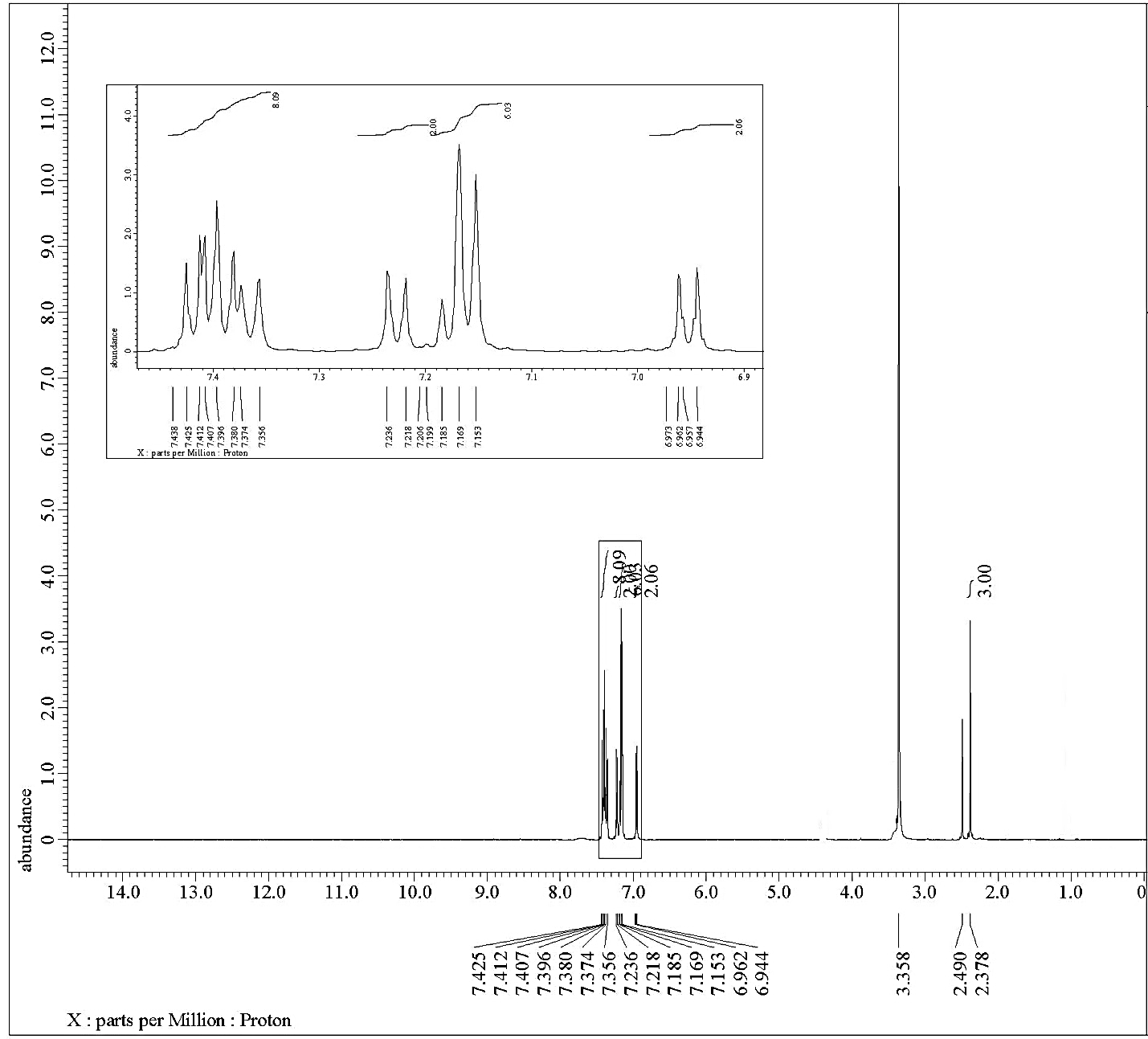
**

**Figure (S26): ^1^H NMR spectrum of compound 4b**

**
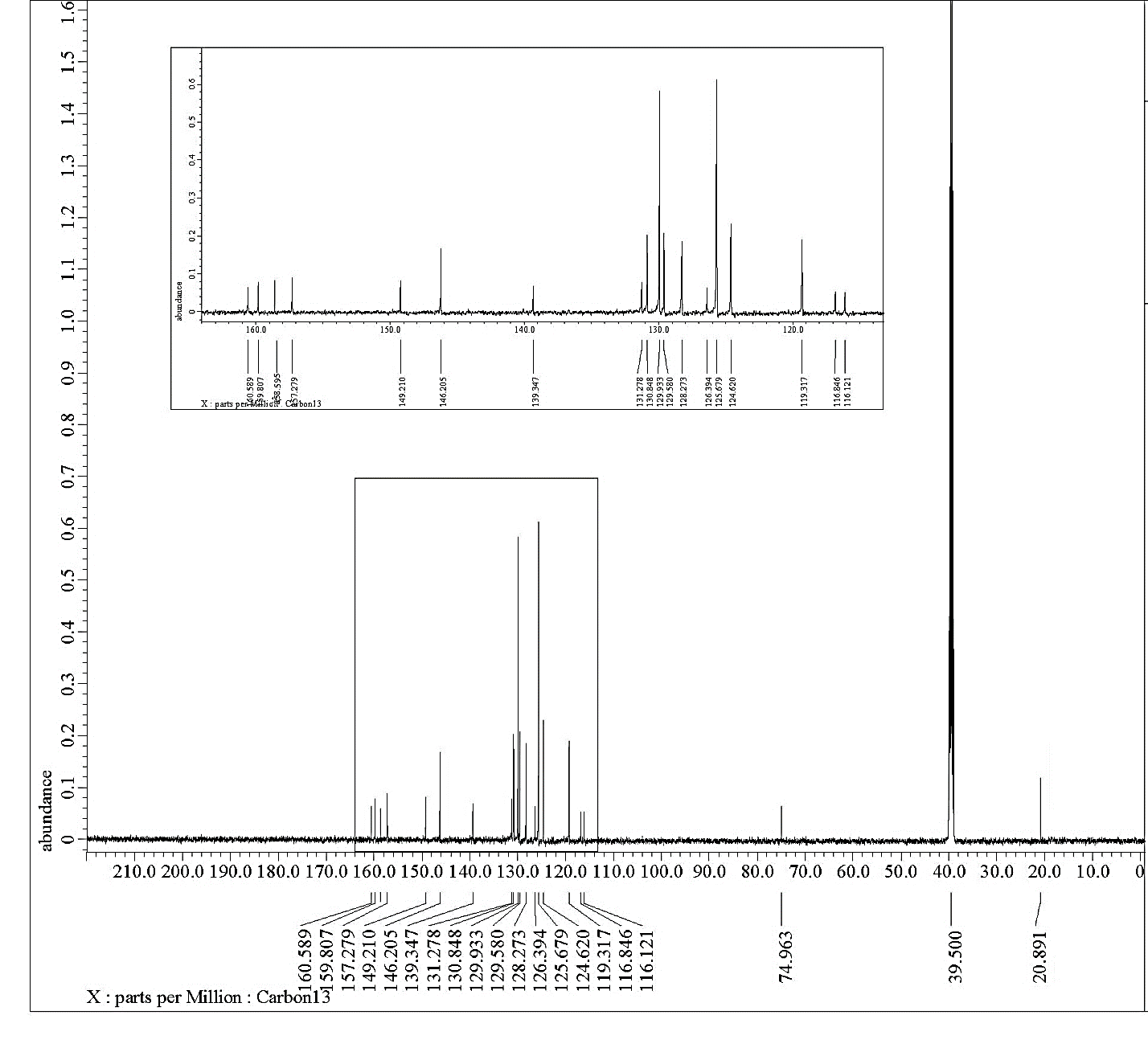
**

**Figure (S27): ^13^C NMR spectrum of compound 4b**

**Figure (S28): Mass spectrum of compound 4b**

**
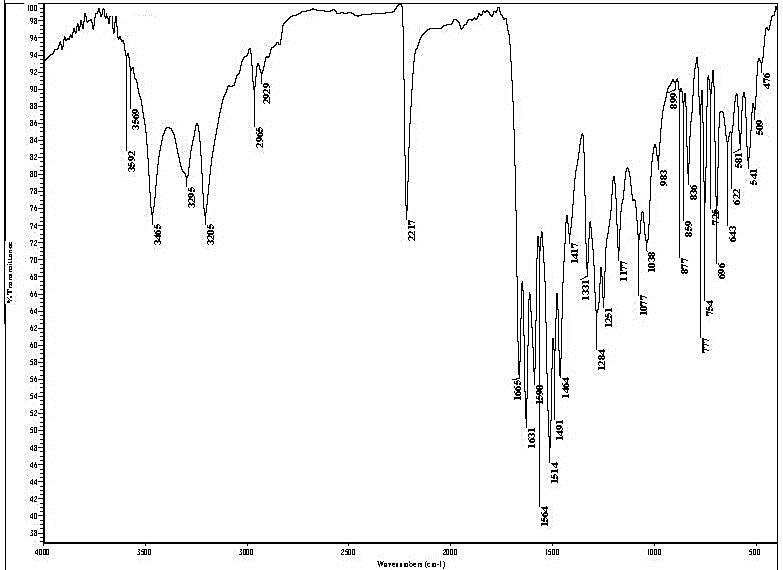
**

**Figure (S29): IR spectrum of compound 4c**

**
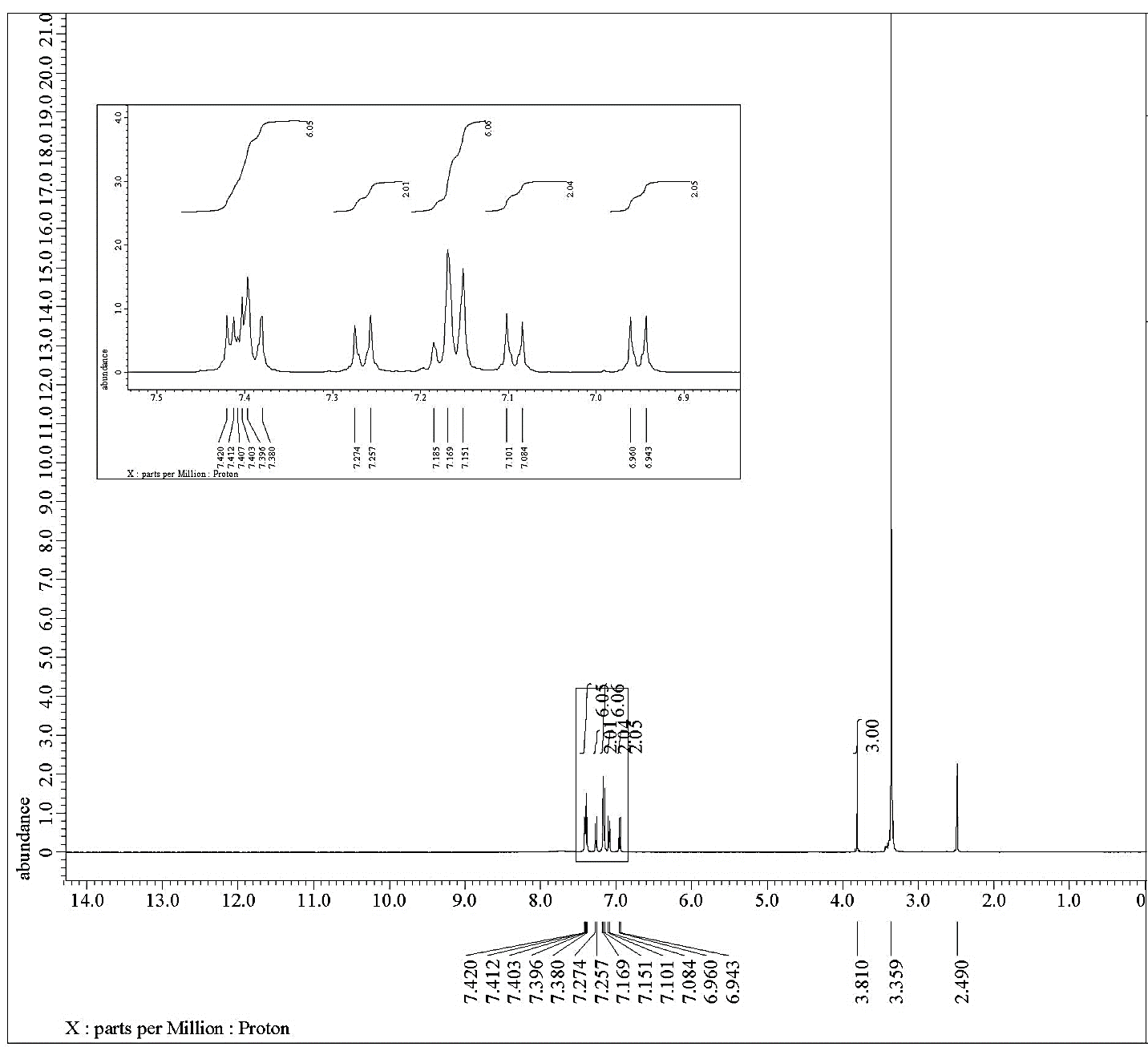
**

**Figure (S30): ^1^H NMR spectrum of compound 4c**

**
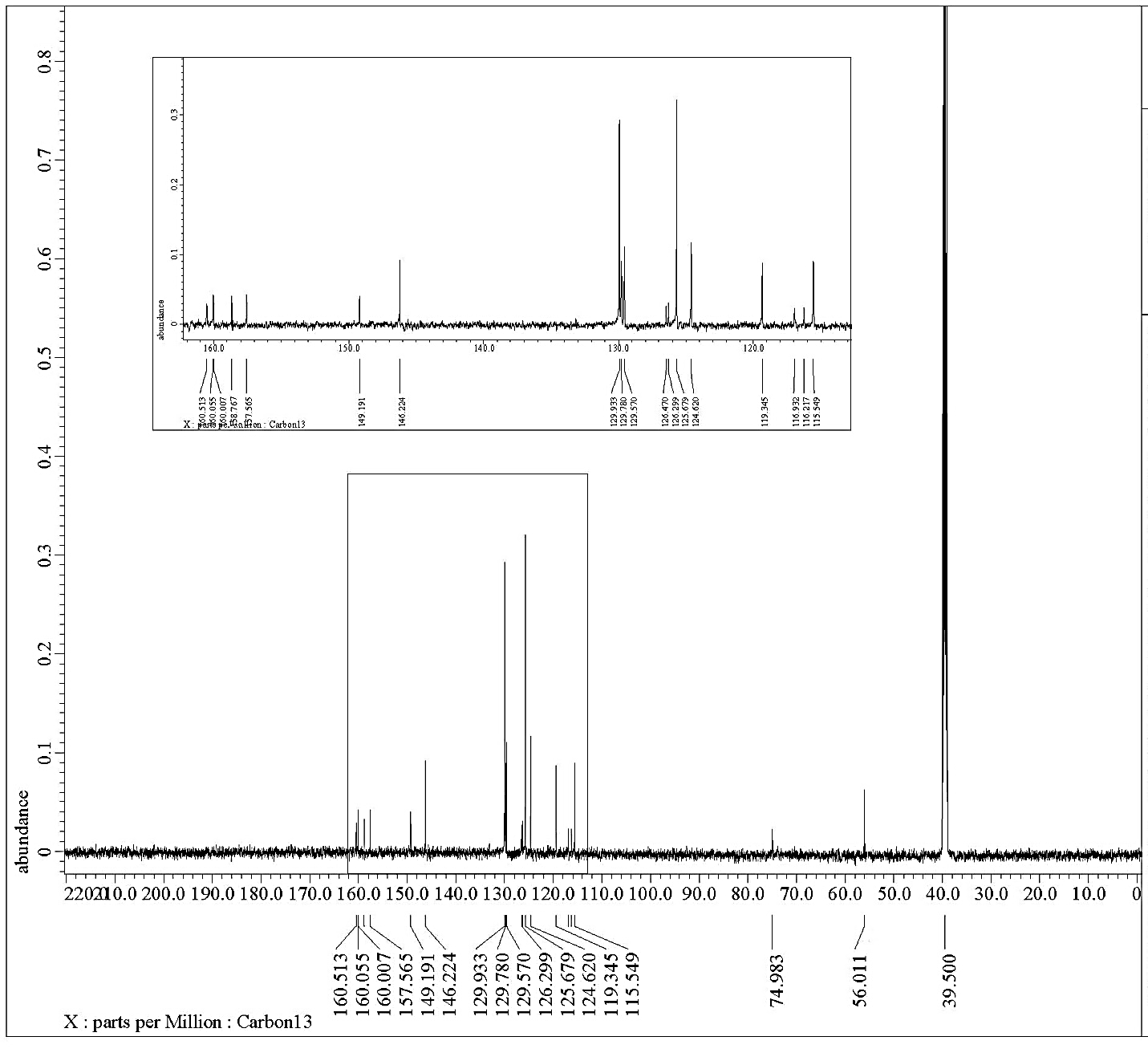
**

**Figure (S31): ^13^C NMR spectrum of compound 4c**

**Figure (S32): Mass spectrum of compound 4c**

**
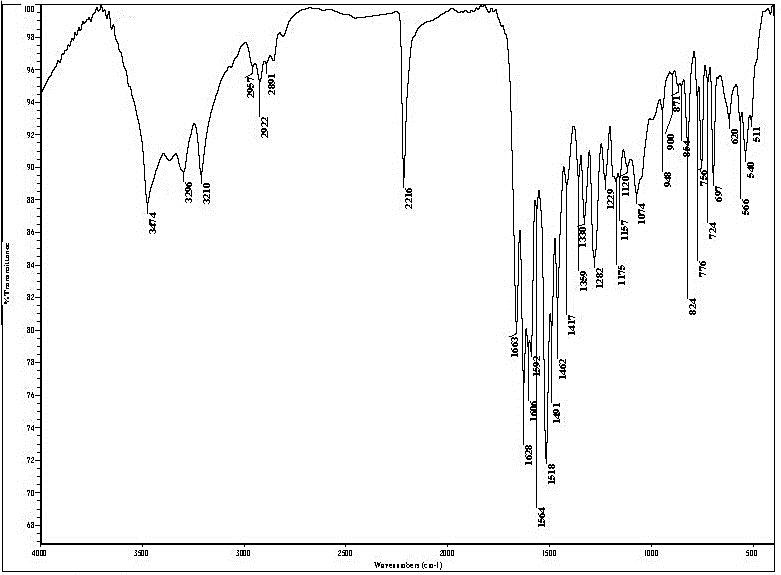
**

**Figure (S33): IR spectrum of compound 4d**

**
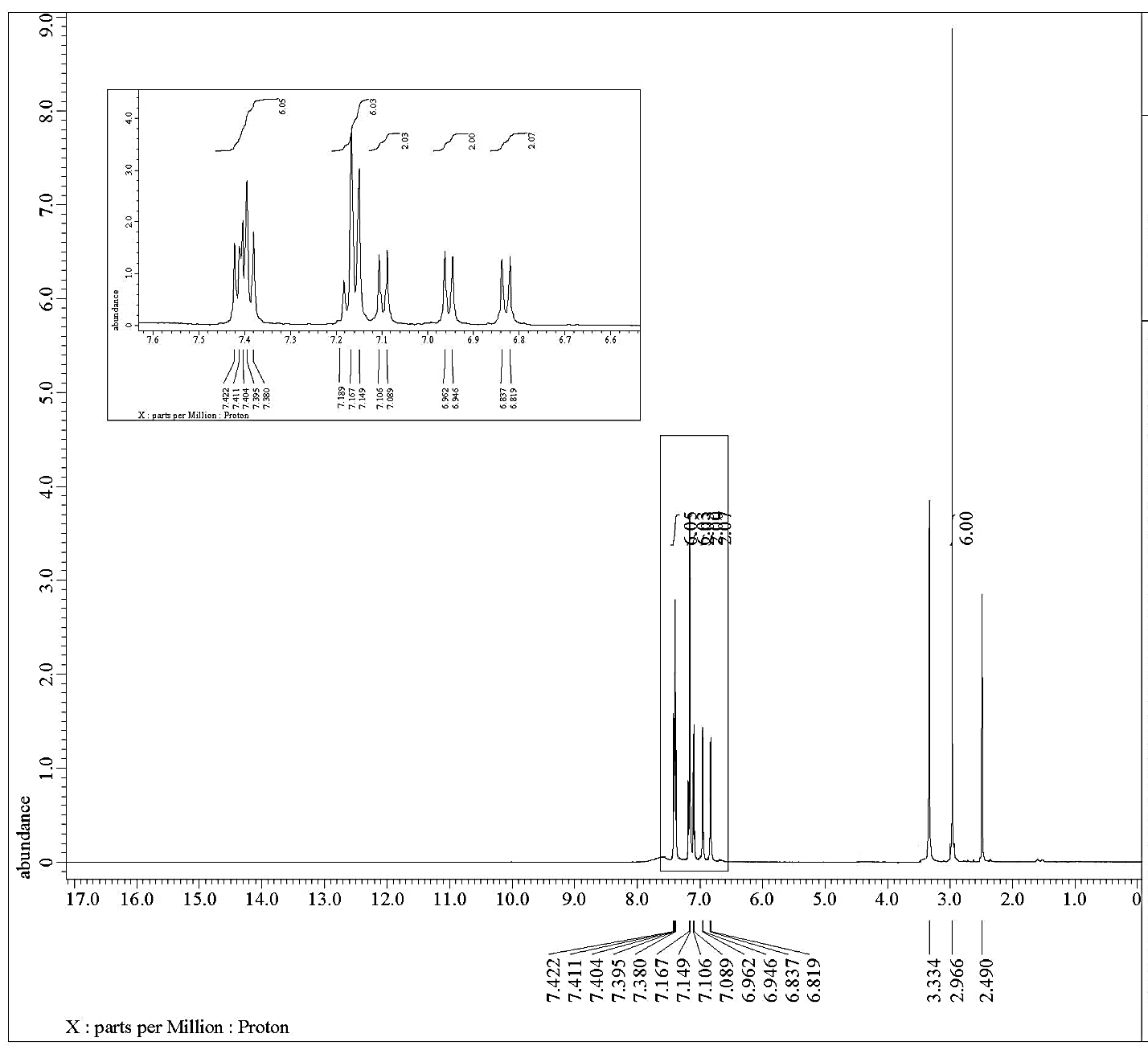
**

**Figure (S34): ^1^H NMR spectrum of compound 4d**

**
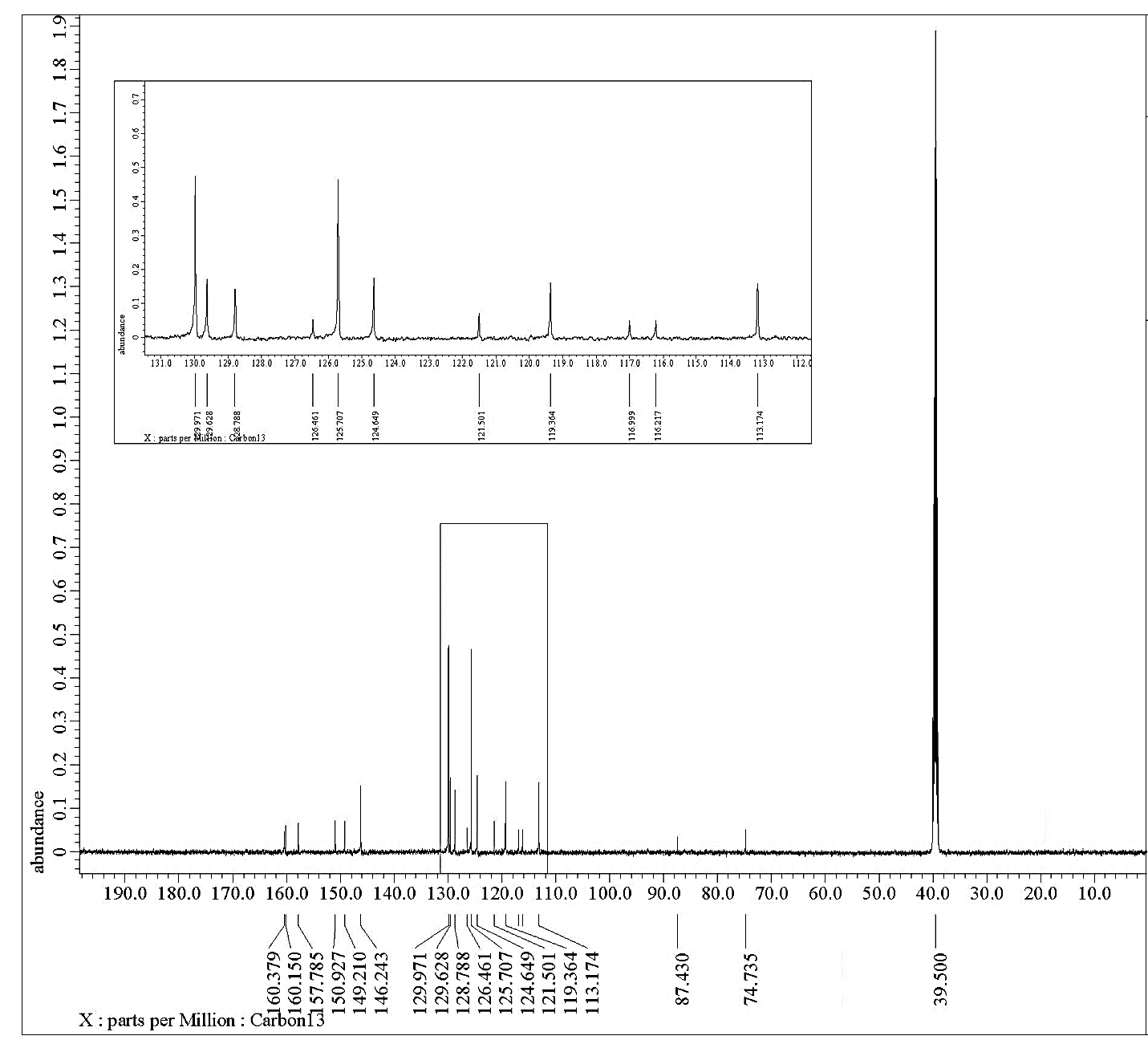
**

**Figure (S35): ^13^C NMR spectrum of compound 4d**

**Figure (S36): Mass spectrum of compound 4d**

**
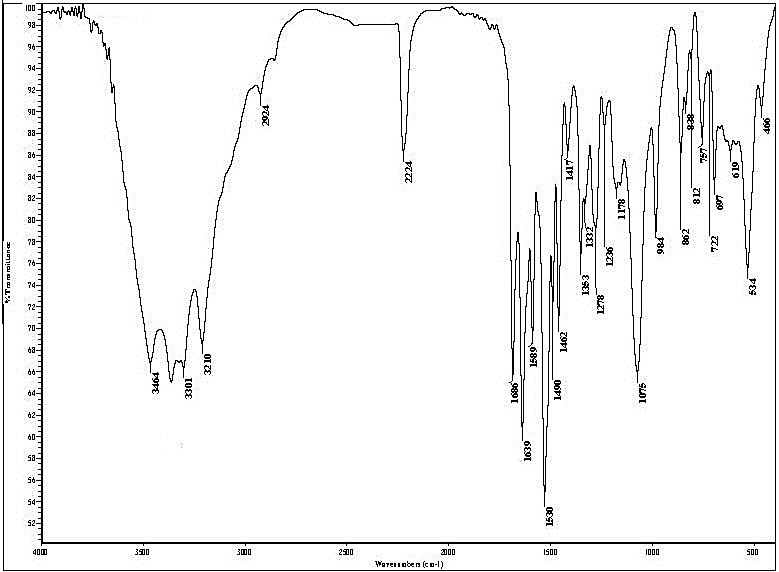
**

**Figure (S37): IR spectrum of compound 4e**

**
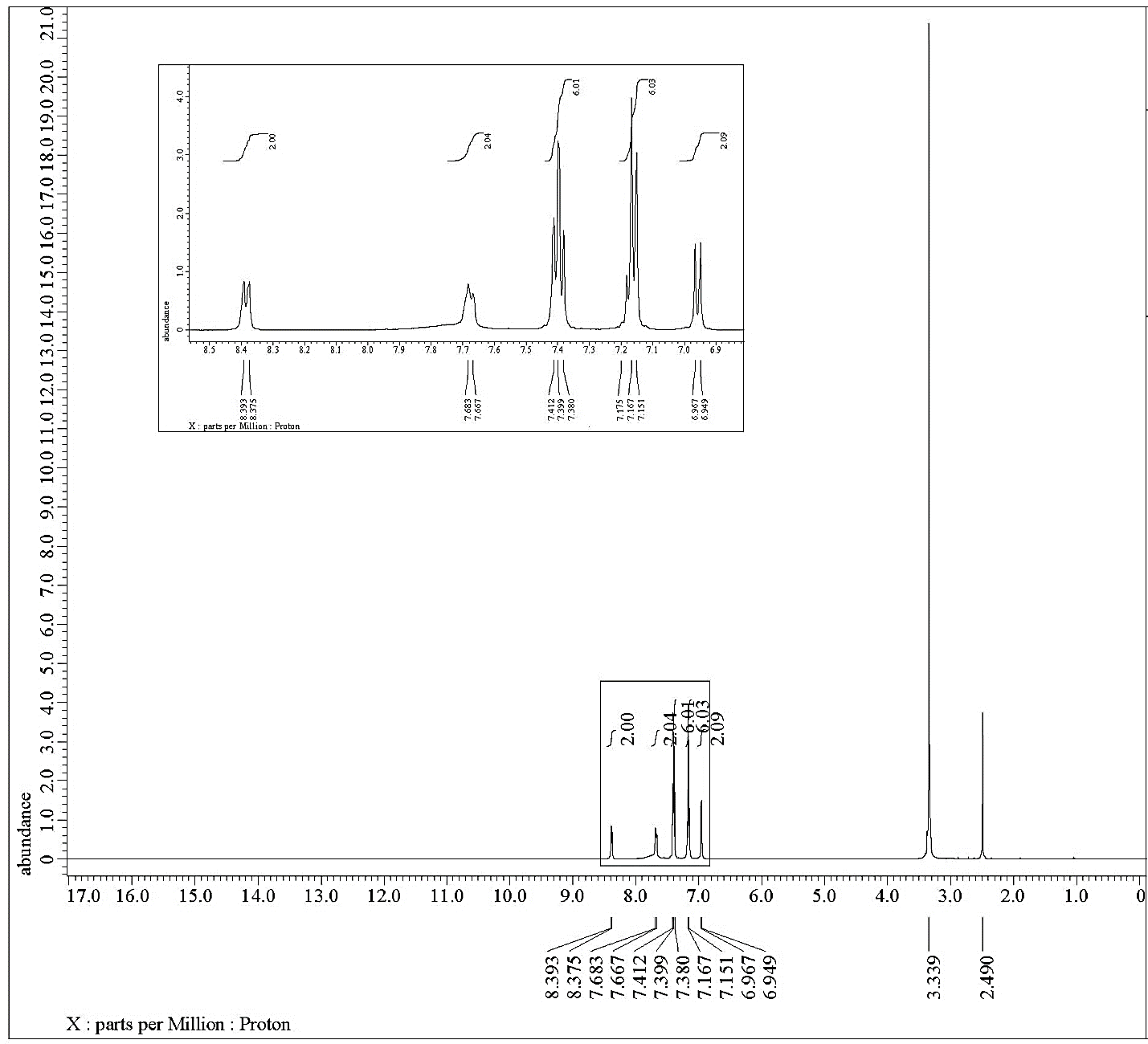
**

**Figure (S38): ^1^H NMR spectrum of compound 4e**

**
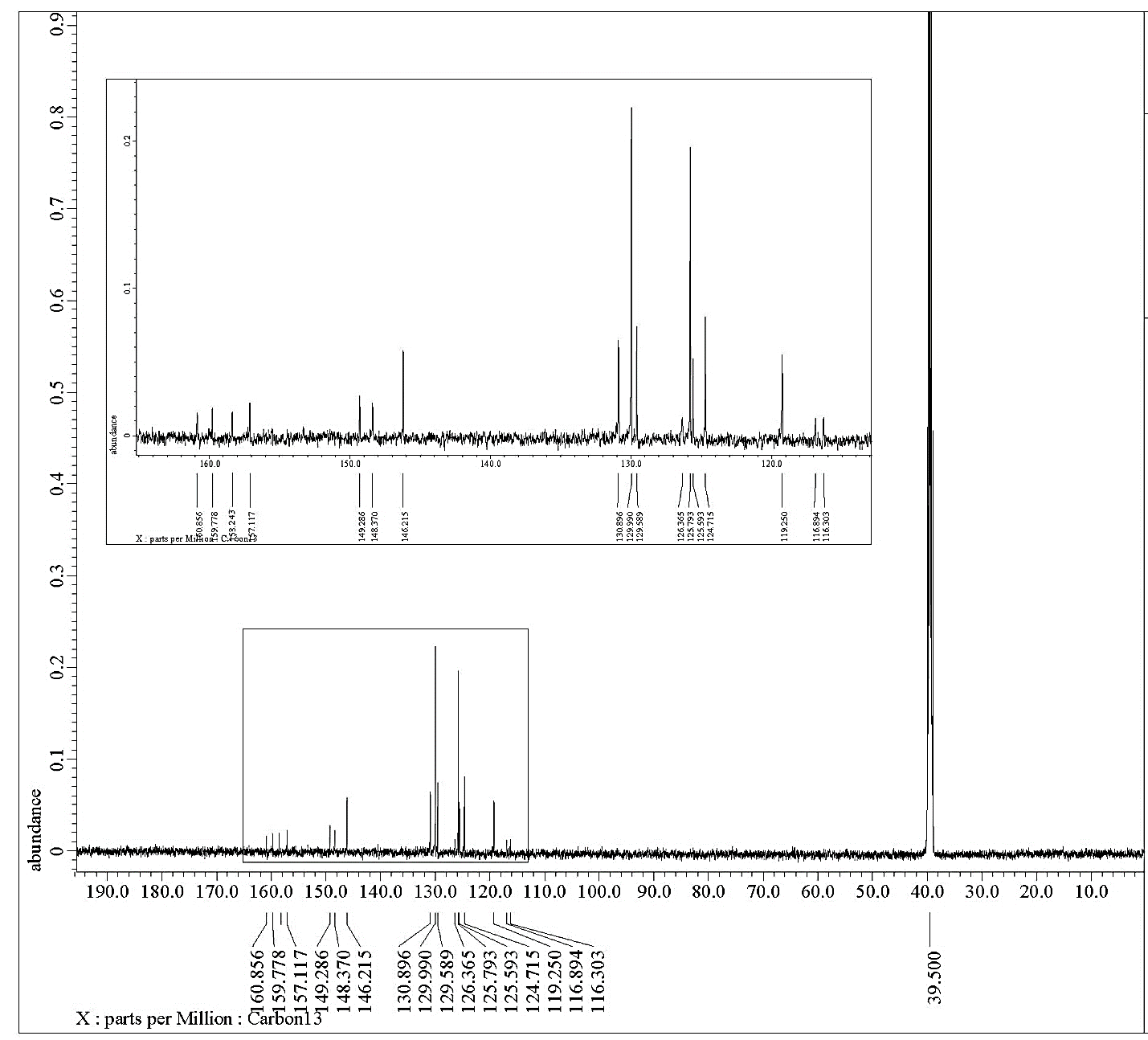
**

**Figure (S39): ^13^C NMR spectrum of compound 4e**

**Figure (S40): Mass spectrum of compound 4e**

**
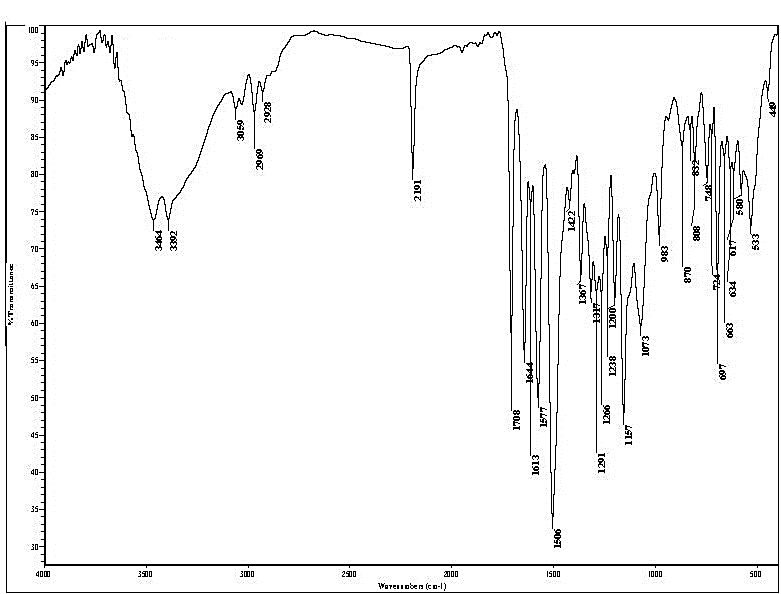
**

**Figure (S41): IR spectrum of compound 9a**

**
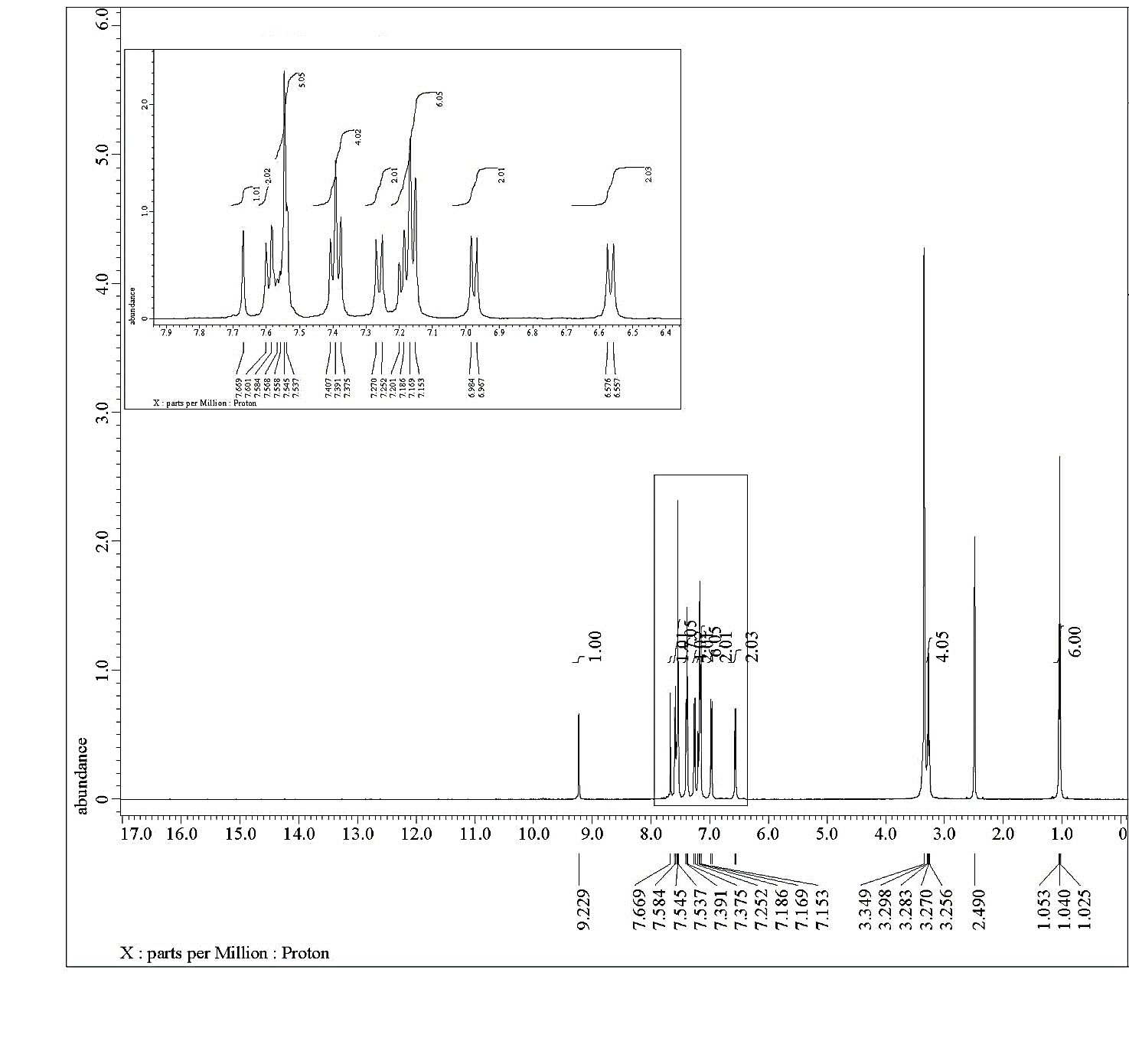
**

**Figure (S42): ^1^H NMR spectrum of compound 6a**

**Figure (S43): Mass spectrum of compound 6a**

**
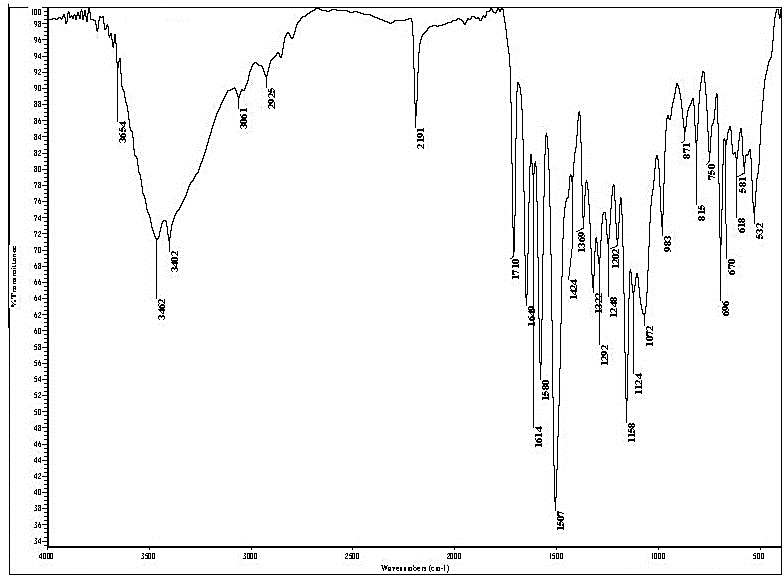
**

**Figure (S44): IR spectrum of compound 6b**

**
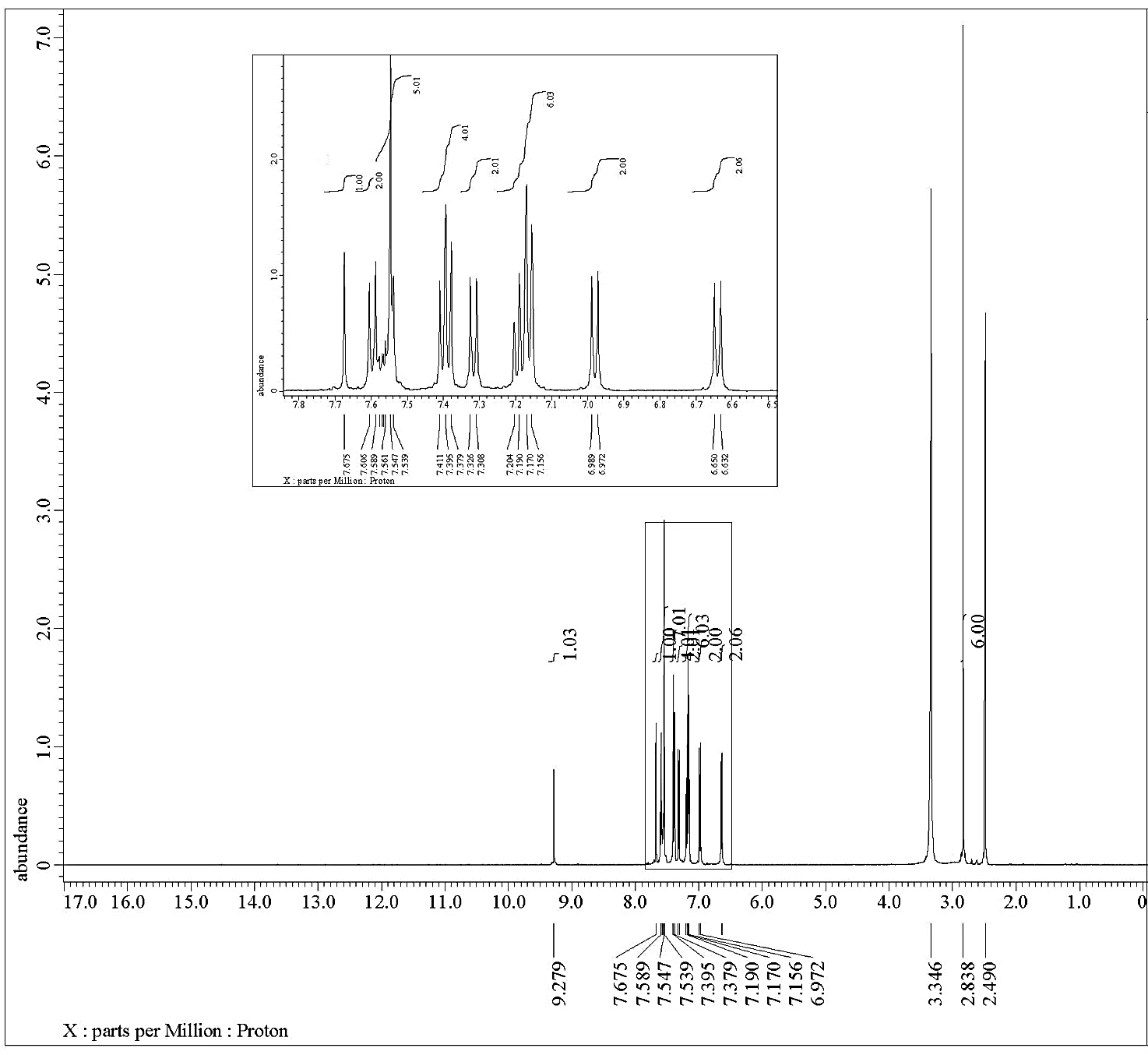
**

**Figure (S45): ^1^H NMR spectrum of compound 6b**

**Figure (S46): Mass spectrum of compound 6b**

**
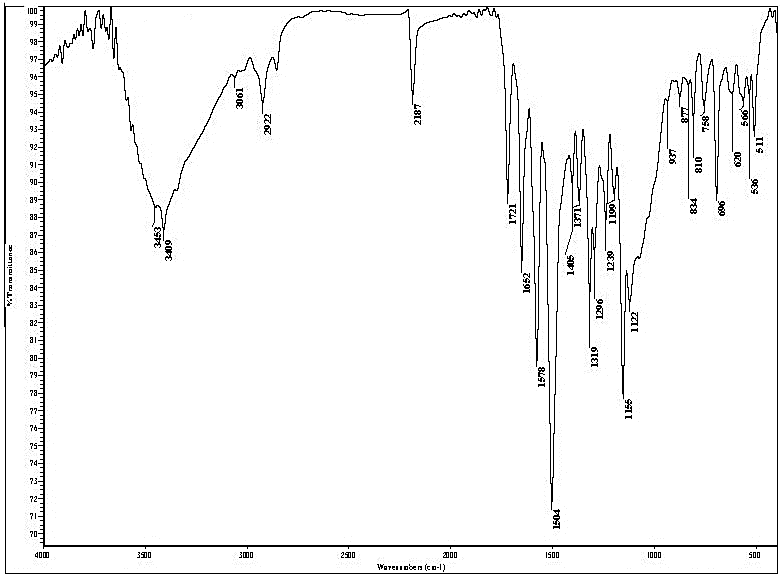
**

**Figure (S47): IR spectrum of compound 6c**

**
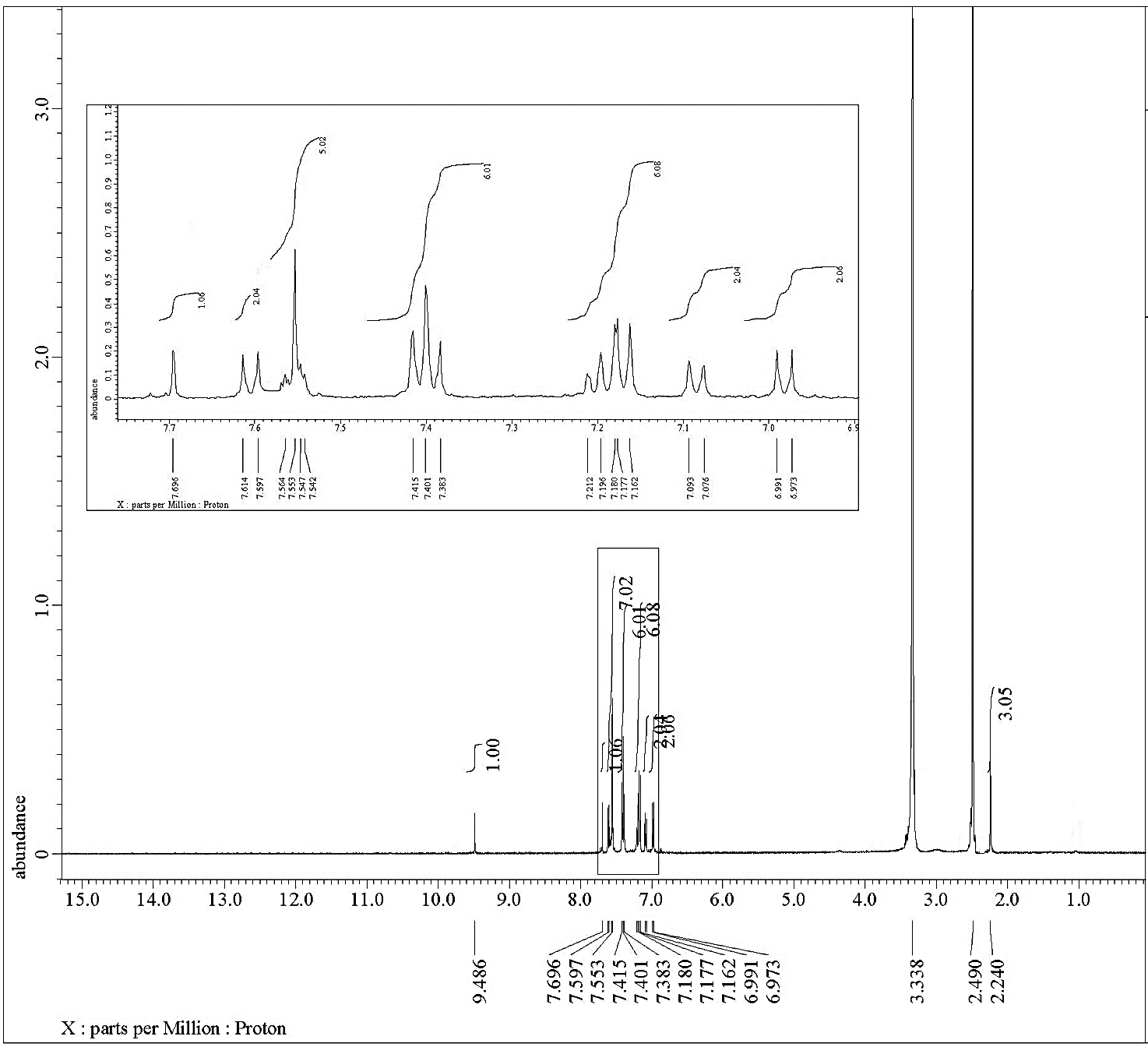
**

**Figure (S48): ^1^H NMR spectrum of compound 6c**

**
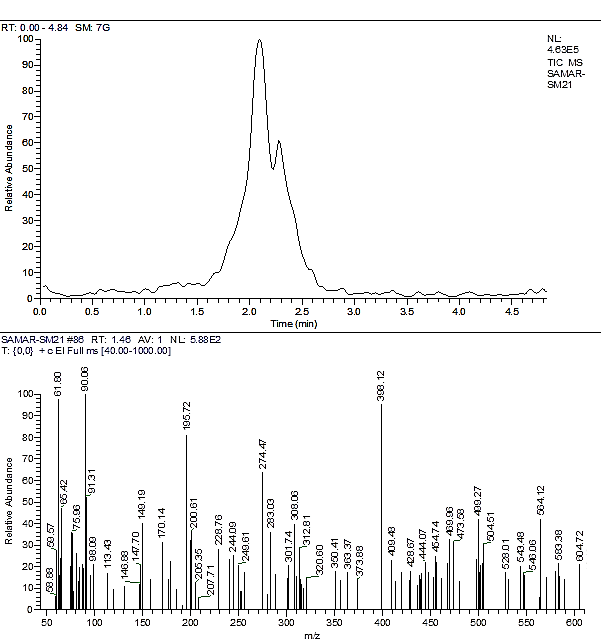
**

**Figure (S49): Mass spectrum of compound 6c**

**
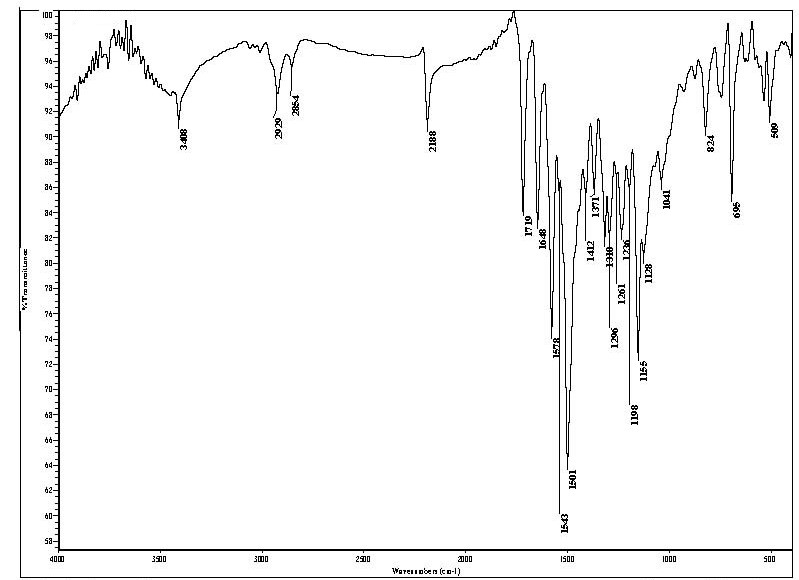
**

**Figure (S50): IR spectrum of compound 6d**

**
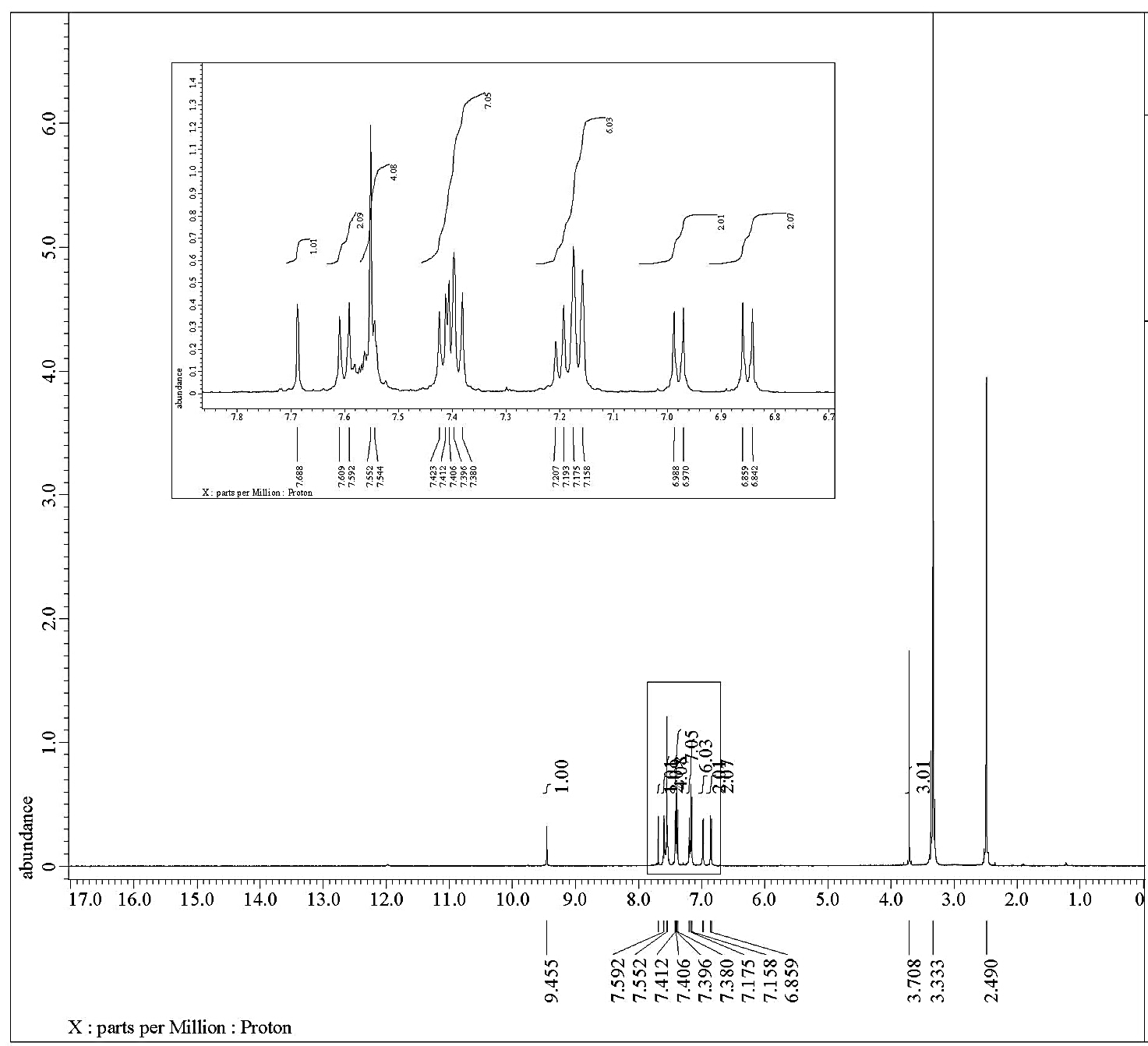
**

**Figure (S51): ^1^H NMR spectrum of compound 6d**

**Figure (S52): Mass spectrum of compound 6d**

**
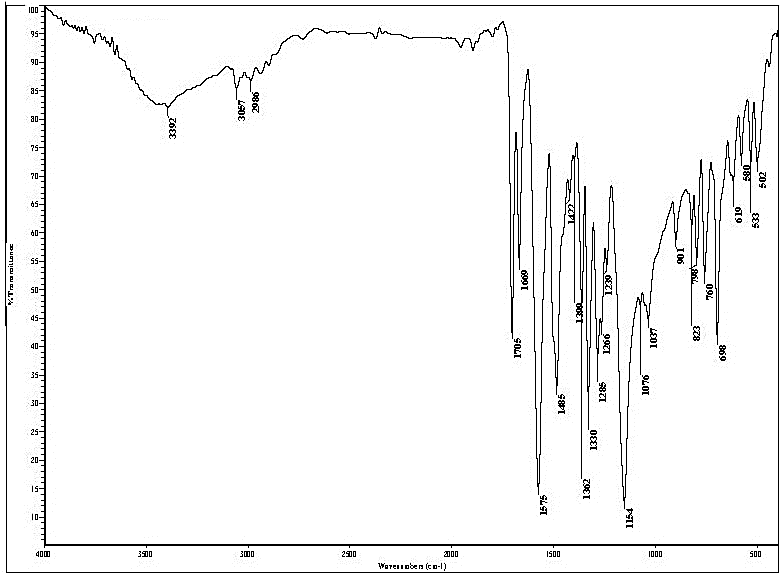
**

**Figure (S53): IR spectrum of compound 8**

**
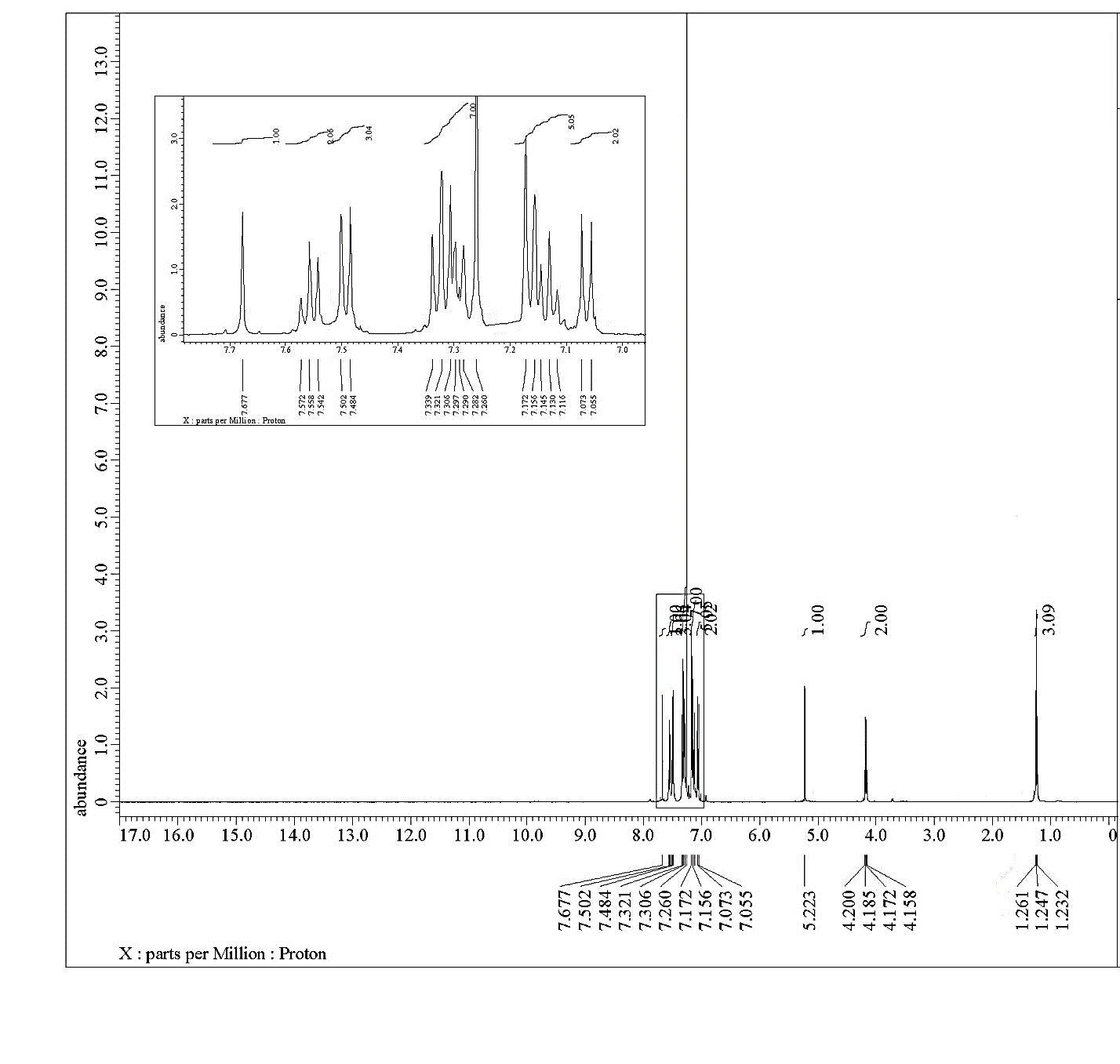
**

**Figure (S54): ^1^H NMR spectrum of compound 8**

**
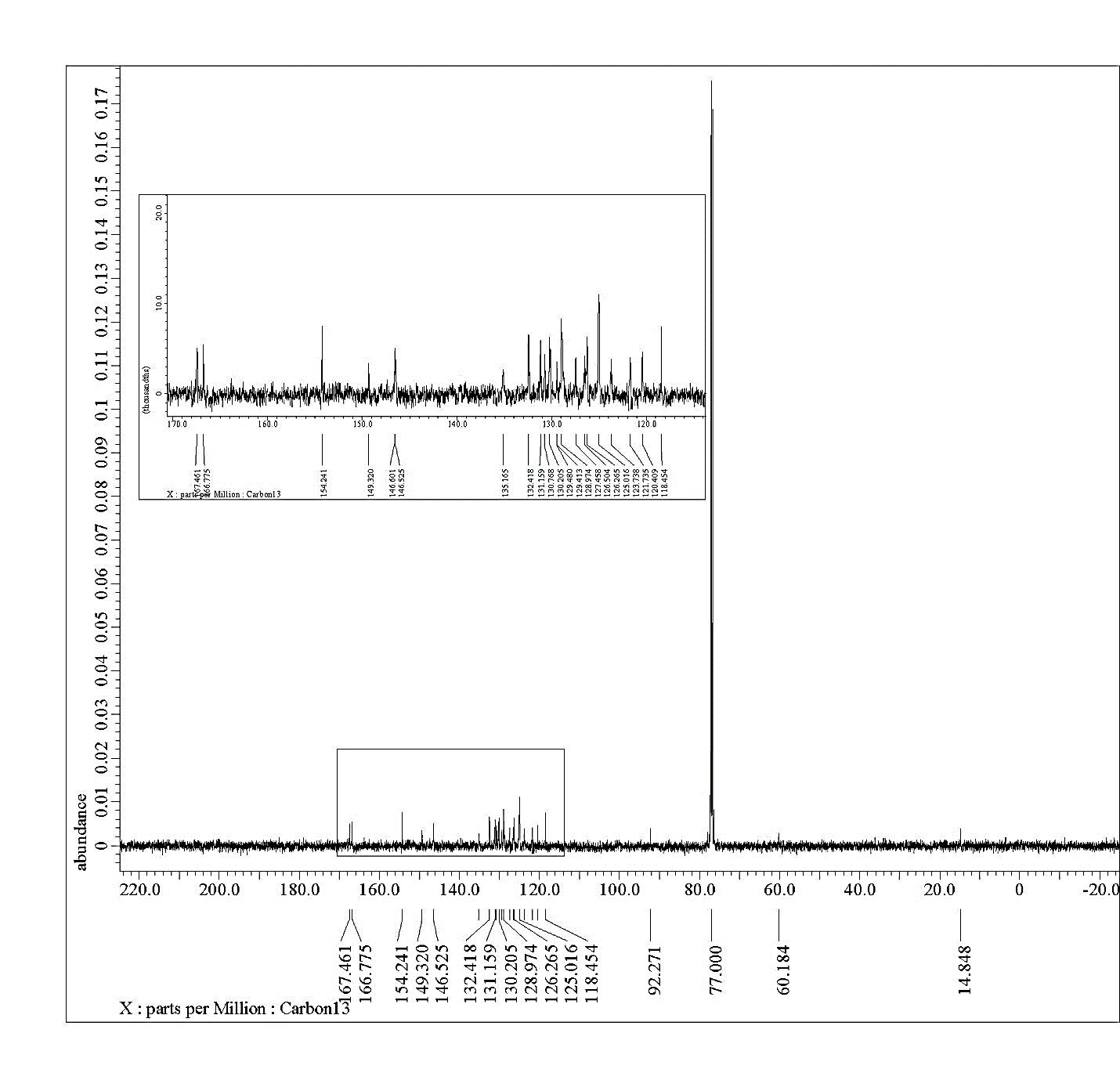
**

**Figure (S55): ^13^C NMR spectrum of compound 8**

**Figure (S56): Mass spectrum of compound 8**

**
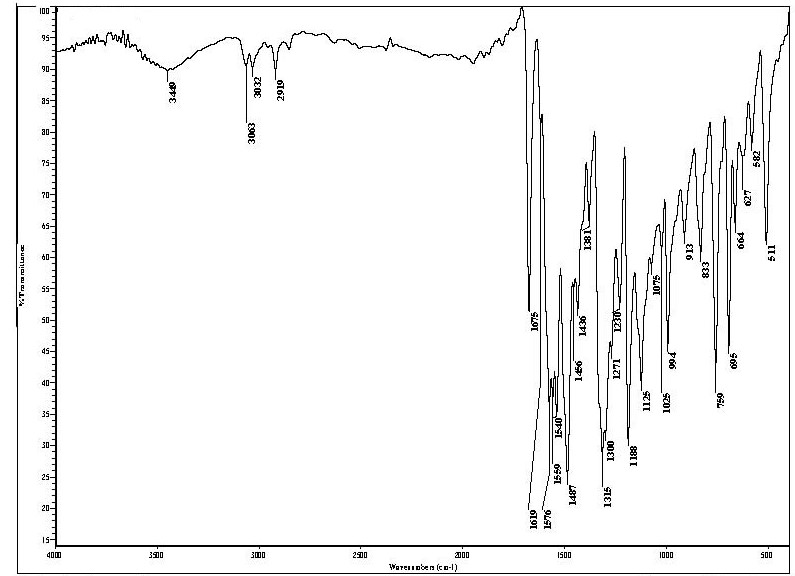
**

**Figure (S57): IR spectrum of compound 9**

**
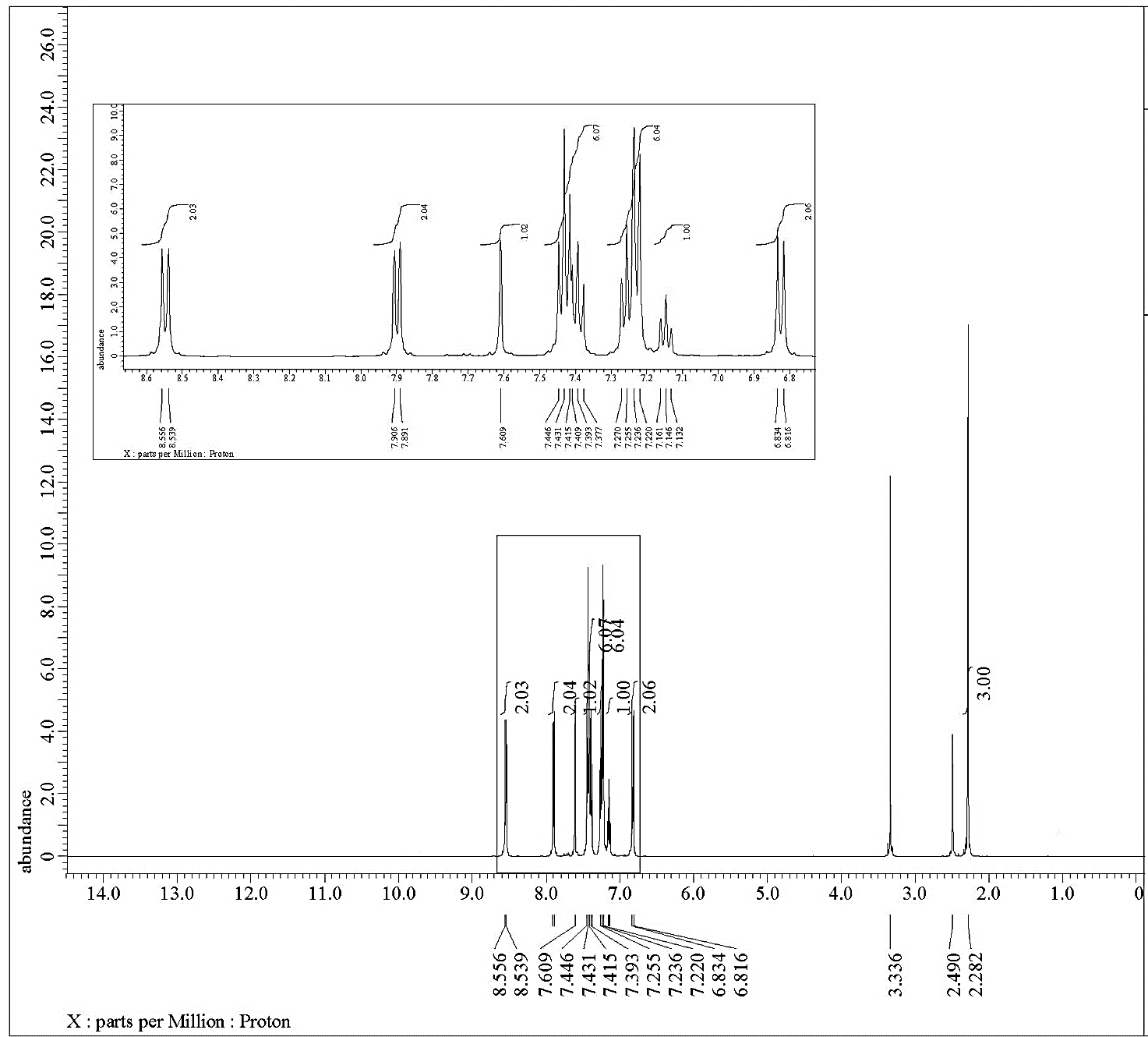
**

**Figure (S58): ^1^H NMR spectrum of compound 9**

**
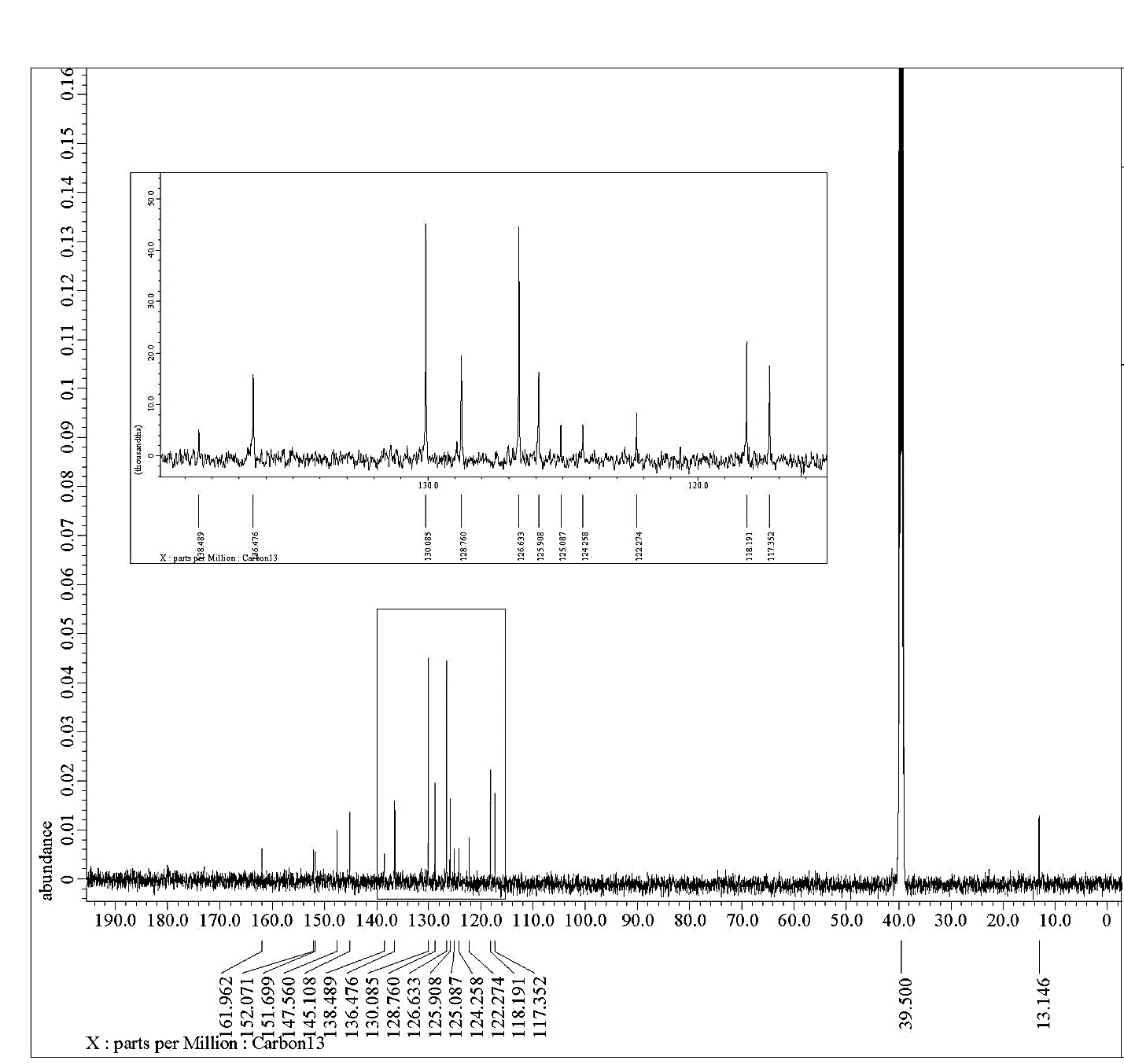
**

**Figure (S59): ^13^C NMR spectrum of compound 9**

**Figure (S60): Mass spectrum of compound 9**

**
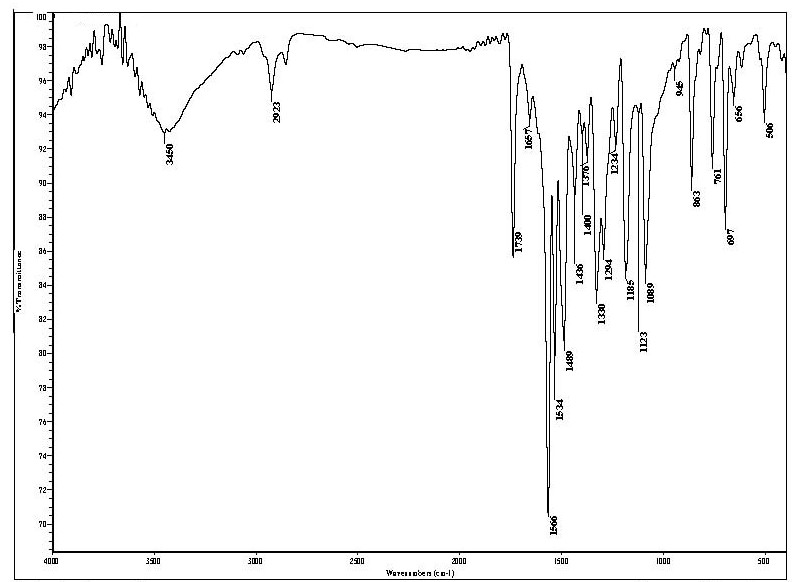
**

**Figure (S61): IR spectrum of compound 10**

**
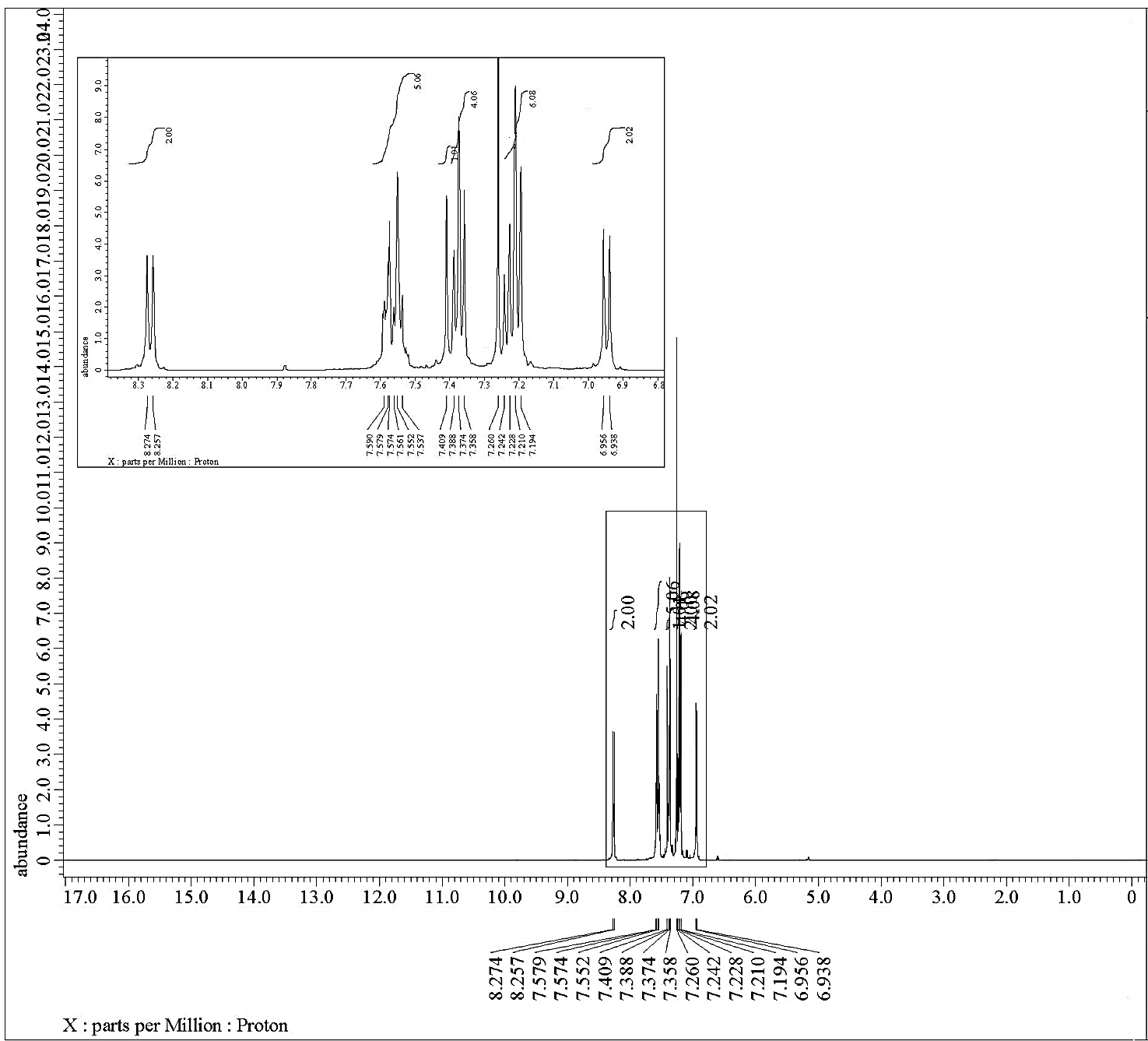
**

**Figure (S62): ^1^H NMR spectrum of compound 10**

**
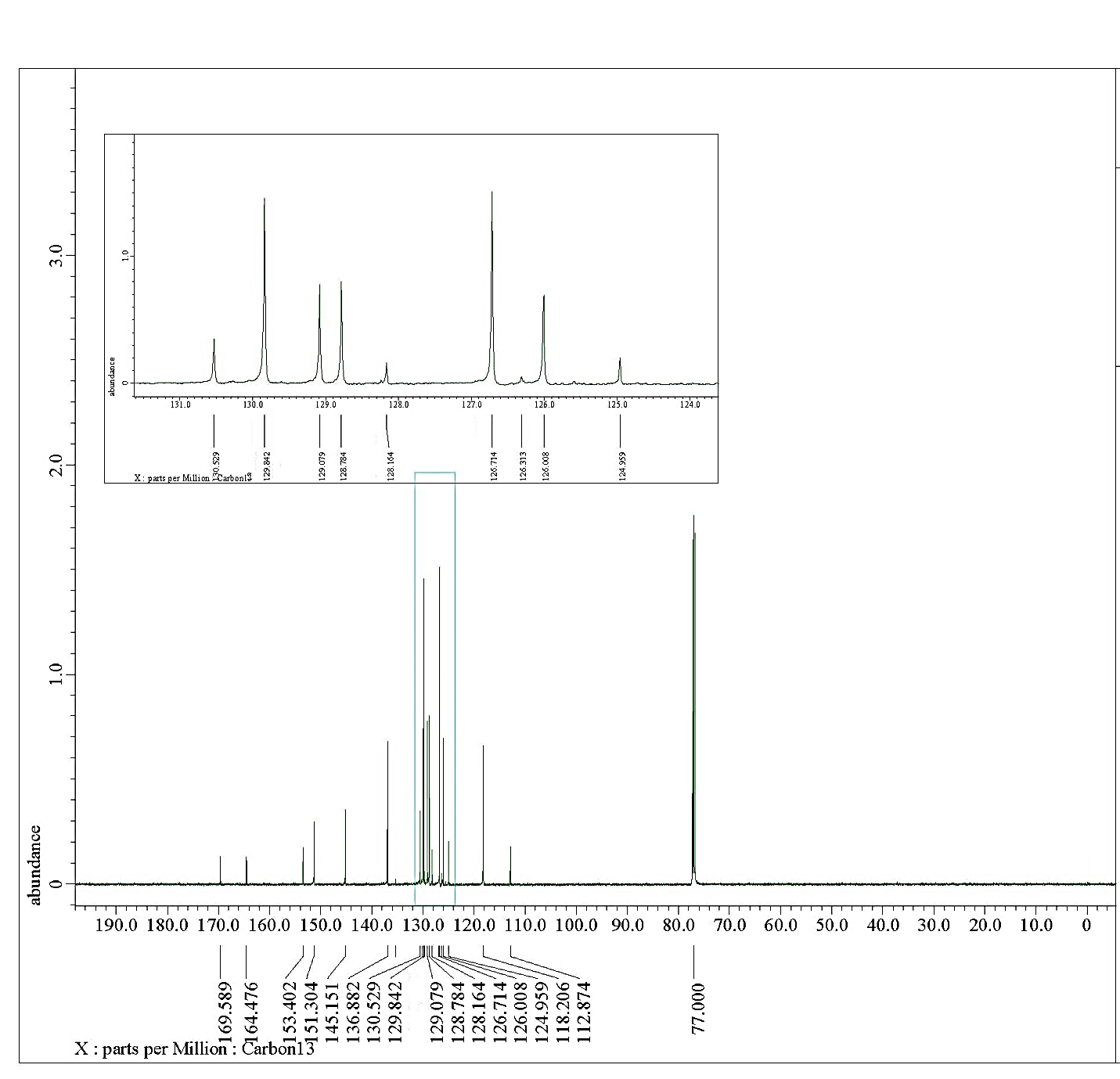
**

**Figure (S63): ^13^C NMR spectrum of compound 10**

**Figure (S64): Mass spectrum of compound 10**

**2. Docking analysis:**

| **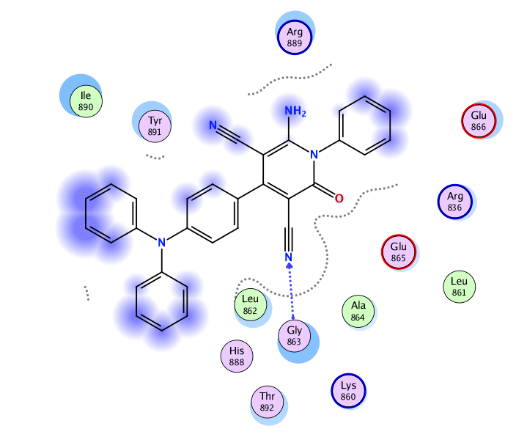** | **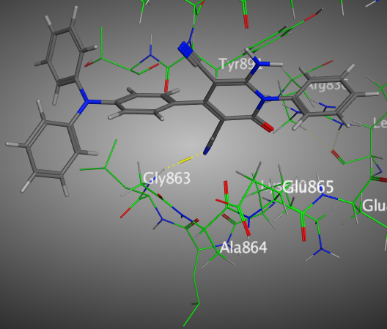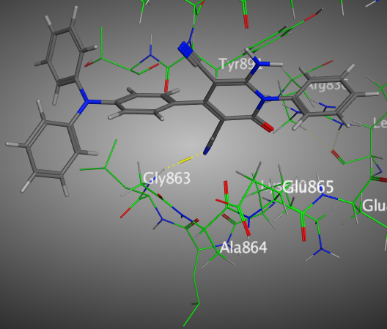** |
| --- | --- |
| 2D | 3D (active sites) |

**Figure (S65):** Interactions of **4a** with the residues of (PDB ID: 2ITO) (blue: H-

acceptor bond).

| 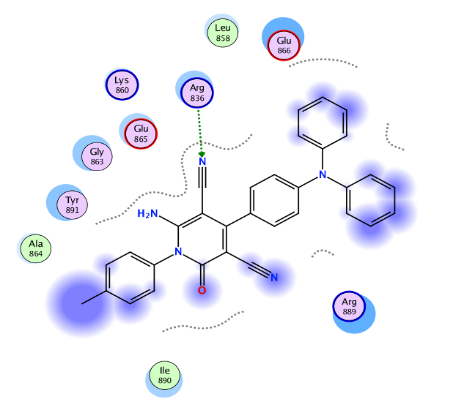 | 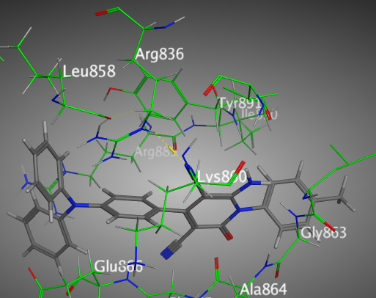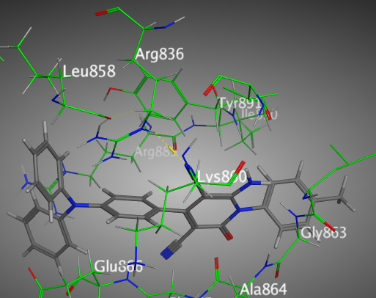 |
| --- | --- |
| 2D | 3D (active sites) |

**Figure (S66):** Interactions of **4b** with the residues of (PDB ID: 2ITO) (green: H-acceptor bond).

| 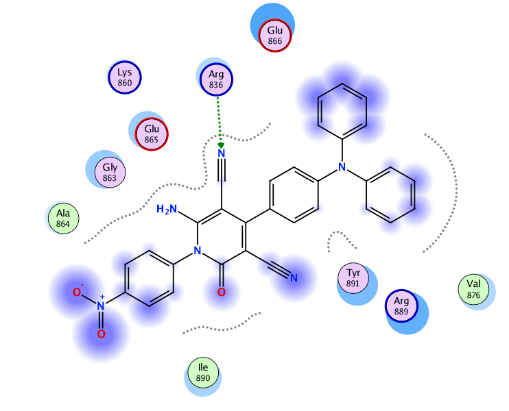 | 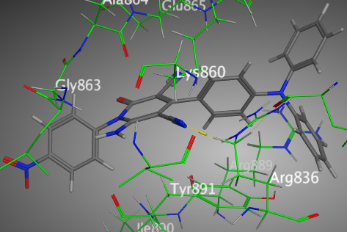 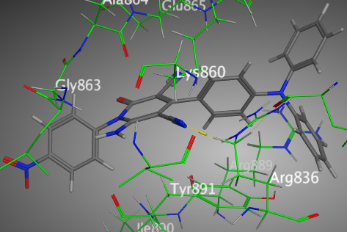 |
| --- | --- |
| 2D | 3D (active sites) |

**Figure (S67):** Interactions of **4e** with the residues of (PDB ID: 2ITO) (green: H-acceptor

bond).

| 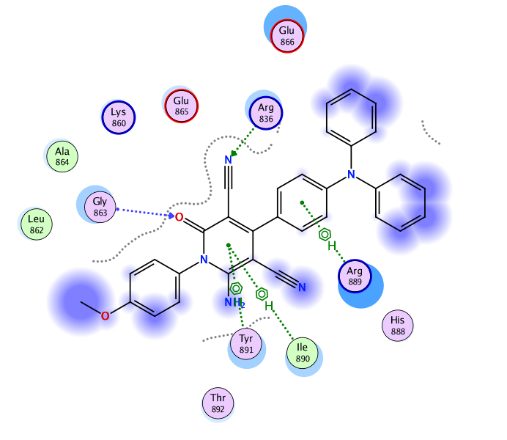 | 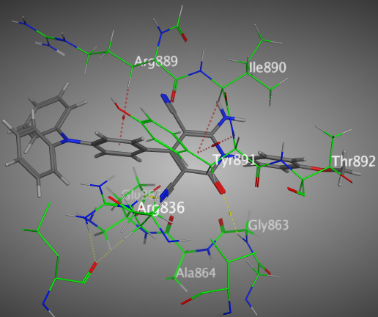 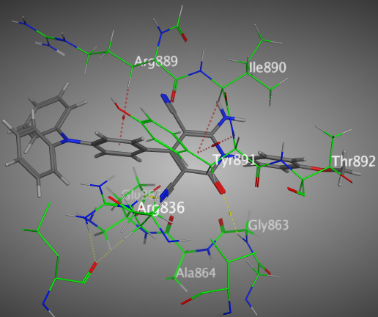 |
| --- | --- |
| 2D | 3D (active sites) |

**Figure (S68):** Interactions of **4c** with the residues of (PDB ID: 2ITO) (blue: H-acceptor bond,

green: H-acceptor bond, green ….….: π-H, green ….….: π-π).

| 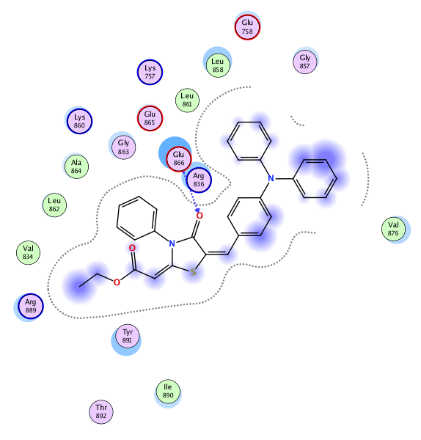 | 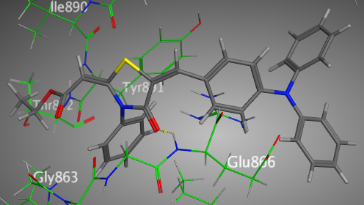 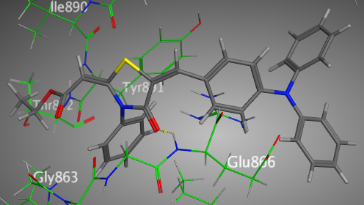 |
| --- | --- |
| 2D | 3D (active sites) |

**Figure (S69):** Interactions of **8** with the residues of (PDB ID: 2ITO) (blue: H-acceptor bond).

| 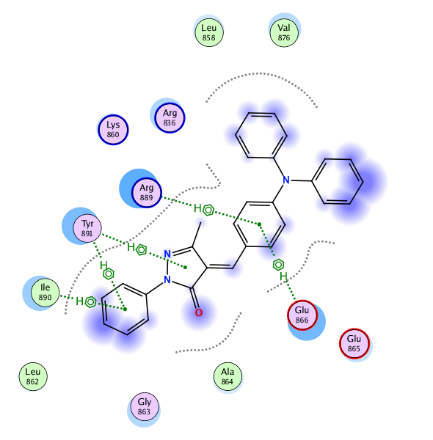 | 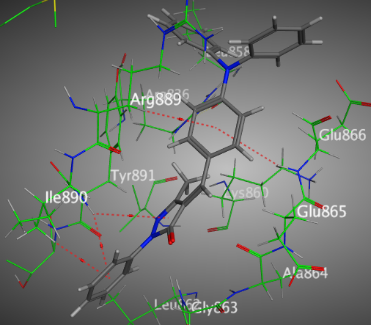 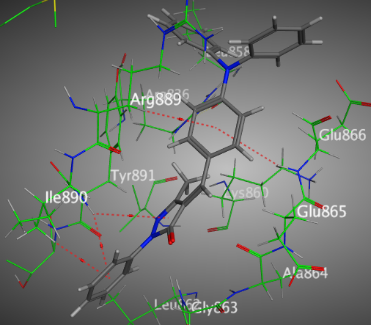 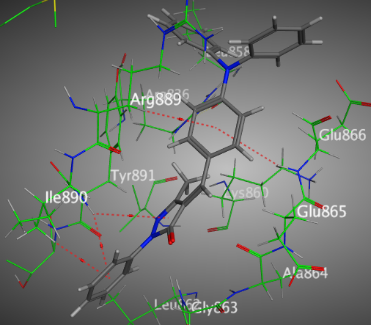 |
| --- | --- |
| 2D | 3D (active sites) |

**Figure (S70):** Interactions of **9** with the residues of (PDB ID: 2ITO) (green ….….: π-H)

| 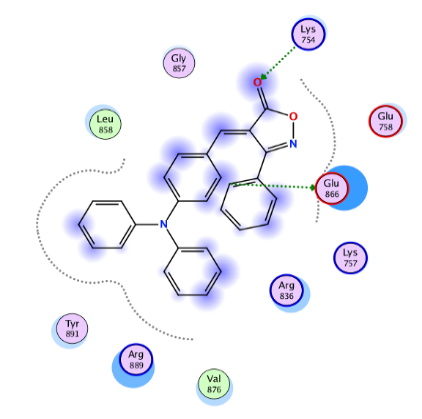 | 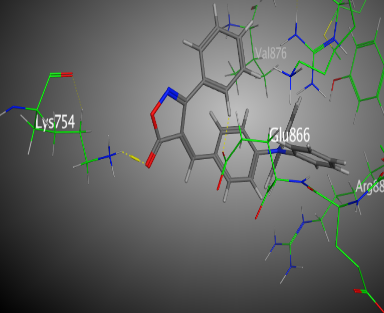 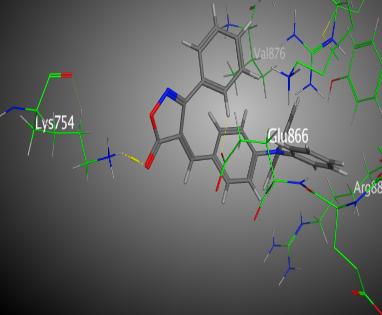 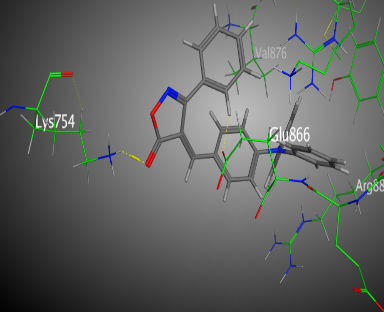 |
| --- | --- |
| 2D | 3D (active sites) |

**Figure (S71):** Interactions of **10** with the residues of (PDB ID: 2ITO) (green: H-donor bond,

green: H-acceptor bond).

| 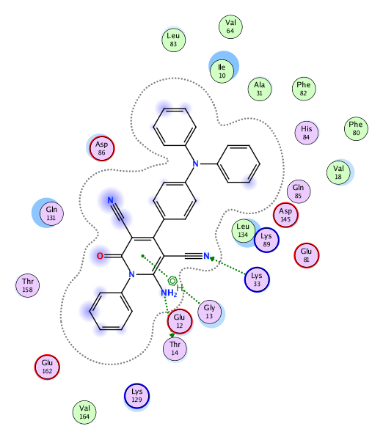 | 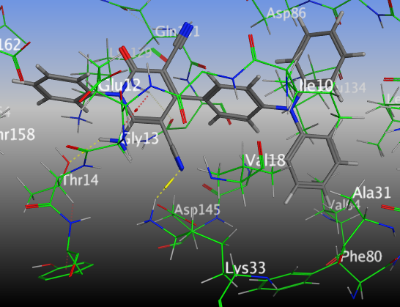 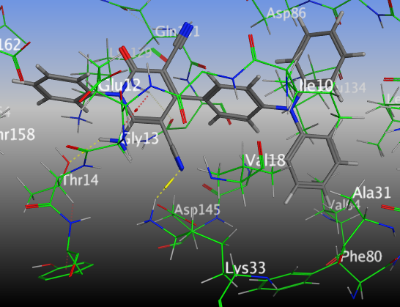 |
| --- | --- |
| 2D | 3D (active sites) |

**Figure (S72):** Interactions of **4a** with the residues of (PDB ID: 2A4L) (green: H-donor bond,

green: H-acceptor bond, green …….: π-H).

| 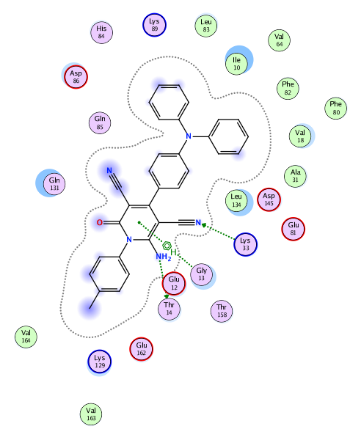 | 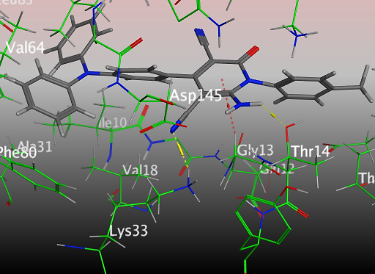 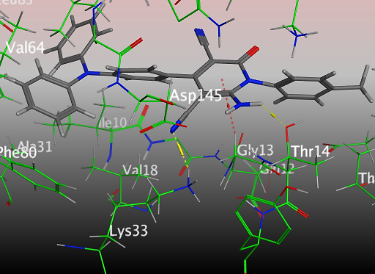 |
| --- | --- |
| 2D | 3D (active sites) |

**Figure (S73):** Interactions of **4b** with the residues of (PDB ID: 2A4L) (green: H-donor bond,

green: H-acceptor bond, green ….….: π-H).

| 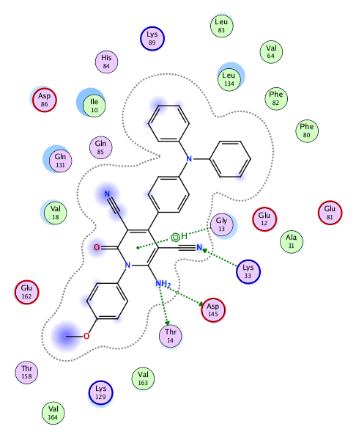 | 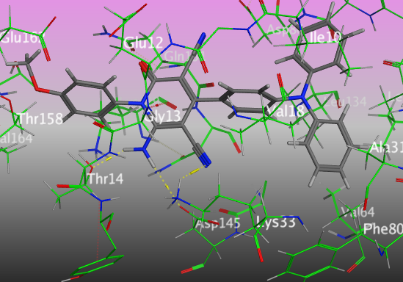 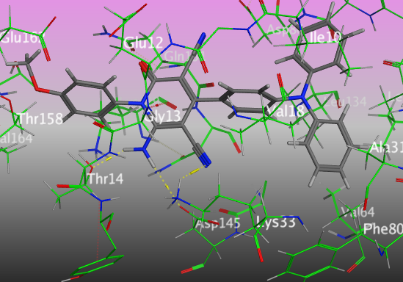 |
| --- | --- |
| 2D | 3D (active sites) |

**Figure (S74):** Interactions of **4c** with the residues of (PDB ID: 2A4L) (green: H-donor bond,

green: H-acceptor bond, green ….….: π-H).

| 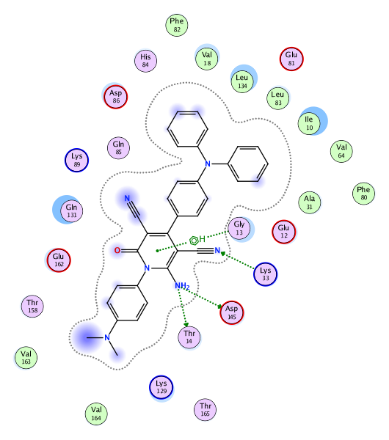 | 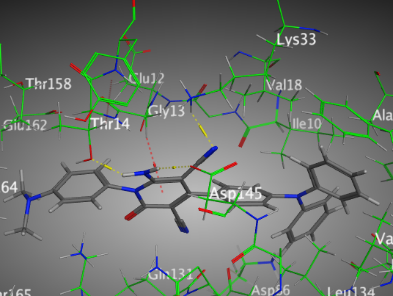 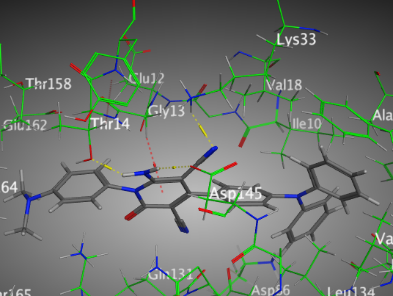 |
| --- | --- |
| 2D | 3D (active sites) |

**Figure (S75):** Interactions of **4d** with the residues of (PDB ID: 2A4L) (green: H-donor bond,

green: H-acceptor bond, green ….….: π-H).

| 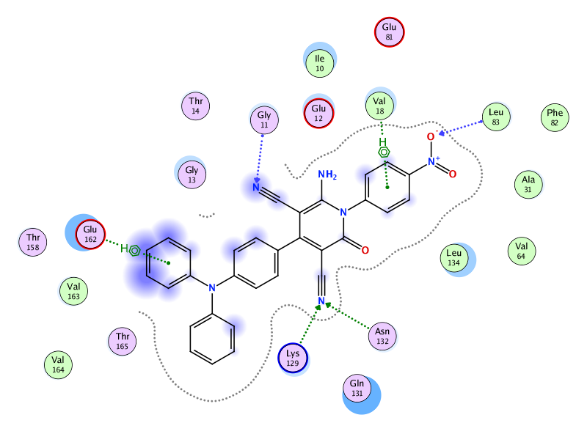 | 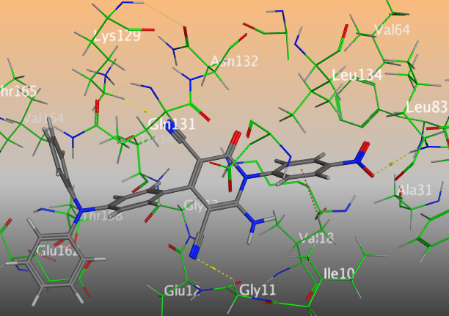 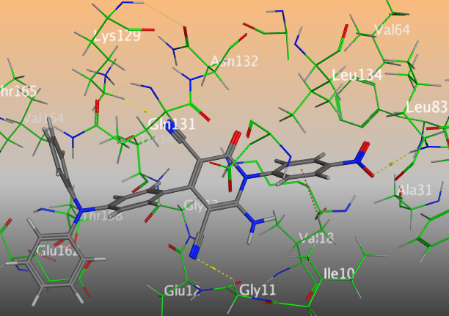  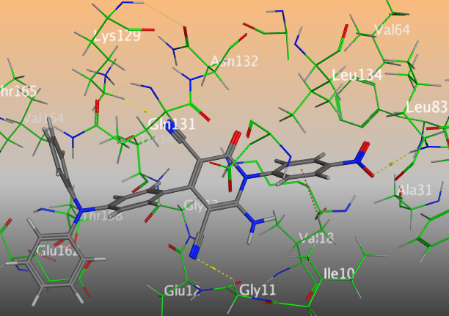 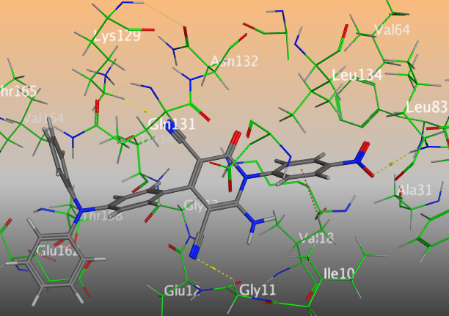 |
| --- | --- |
| 2D | 3D (active sites) |

**Figure (S76):** Interactions of **4e** with the residues of (PDB ID: 2A4L) (green: H-acceptor

bond, blue: H-acceptor bond, green ….….: π-H).

| 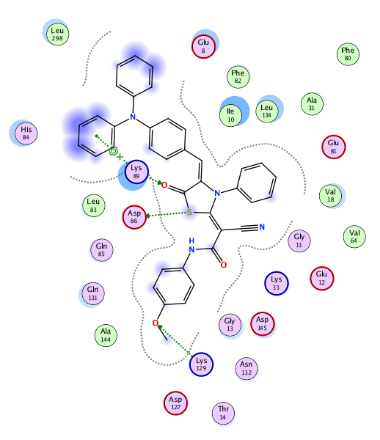 | 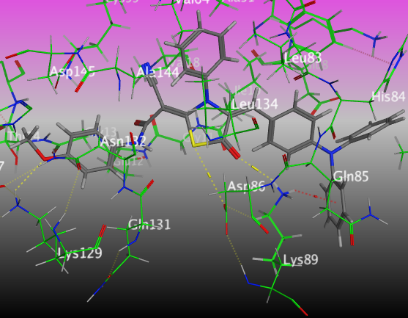 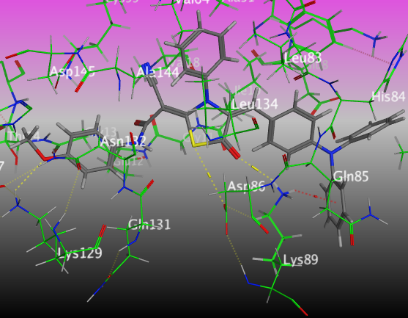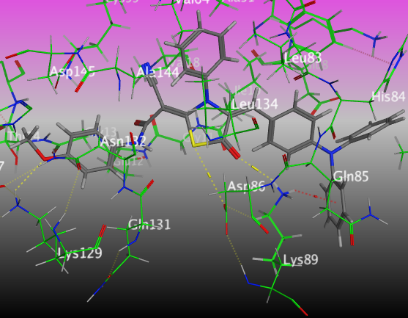 |
| --- | --- |
| 2D | 3D (active sites) |

**Figure (S77):** Interactions of **6d** with the residues of (PDB ID: 2A4L) (green: H-donor

bond, green: H-acceptor bond, green ….….: π-H).

| 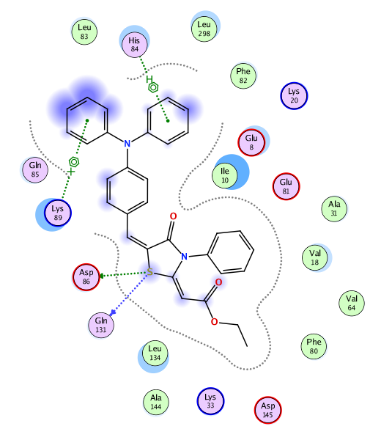 | 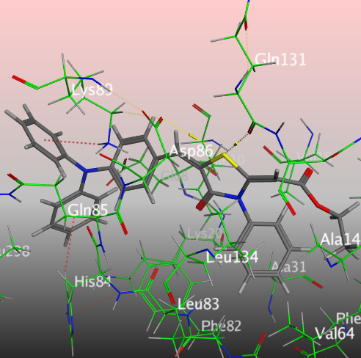 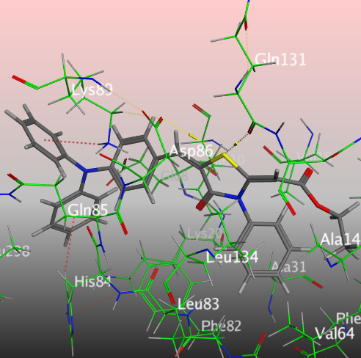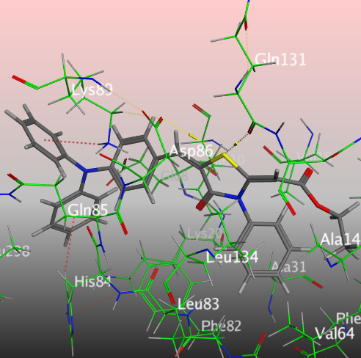 |
| --- | --- |
| 2D | 3D (active sites) |

**Figure (S78):** Interactions of **8** with the residues of (PDB ID: 2A4L) (green: H-donor bond,

blue: H-donor bond, green ….….: π-H, green …..…….. : π-cation).

| 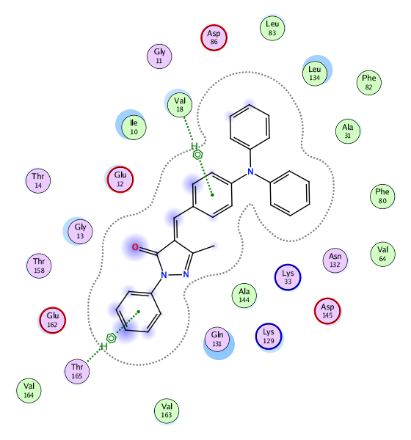 | 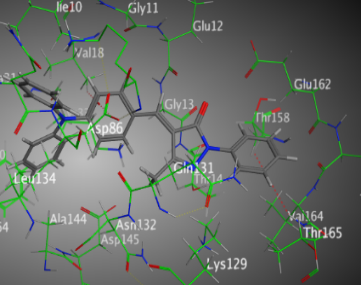 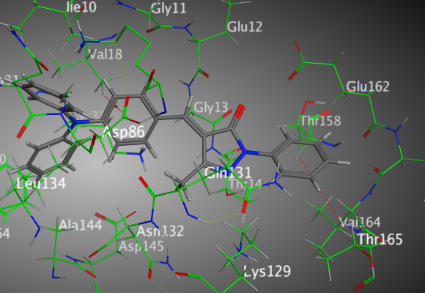  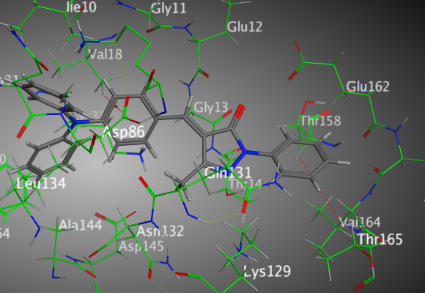 |
| --- | --- |
| 2D | 3D (active sites) |

**Figure (S79):** Interactions of **9** with the residues of (PDB ID: 2A4L) (green ….….: π-H).

| 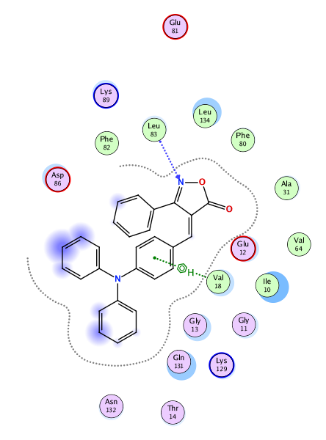 | 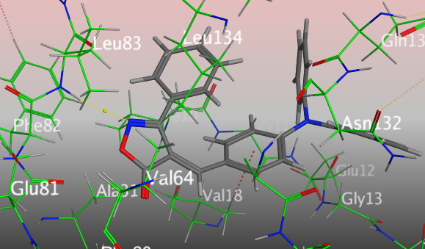 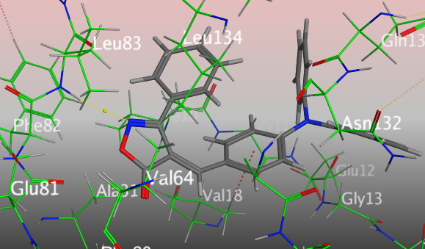  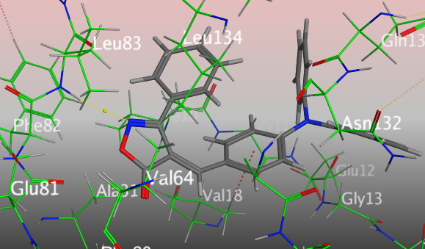 |
| --- | --- |
| 2D | 3D (active sites) |

**Figure (S80):** Interactions of **10** with the residues of (PDB ID: 2A4L) (blue: H-acceptor

bond, green ….….: π-H

|  |  |
| --- | --- |
| 2D | 3D (active sites) |

**Figure (S81):** Interactions of **cisplatin** with the residues of (PDB ID: 2ITO) (blue: H-donor

bond, green: H-donor bond).

|  |  |
| --- | --- |
| 2D | 3D (active sites) |

**Figure (S82):** Interactions of **cisplatin** with the residues of (PDB ID: 2A4L) (pink: ionic bond)
